# The Beaver’s Phylogenetic Lineage *Illumina*ted by Retroposon Reads

**DOI:** 10.1038/srep43562

**Published:** 2017-03-03

**Authors:** Liliya Doronina, Andreas Matzke, Gennady Churakov, Monika Stoll, Andreas Huge, Jürgen Schmitz

**Affiliations:** 1 Institute of Experimental Pathology (ZMBE), University of Münster, Münster, Germany; 2 Institute for Evolution and Biodiversity, University of Münster, Münster, Germany; 3 Core Facility Genomics, Medical Faculty, University of Münster, Münster, Germany

## Abstract

Solving problematic phylogenetic relationships often requires high quality genome data. However, for many organisms such data are still not available. Among rodents, the phylogenetic position of the beaver has always attracted special interest. The arrangement of the beaver’s masseter (jaw-closer) muscle once suggested a strong affinity to some sciurid rodents (e.g., squirrels), placing them in the Sciuromorpha suborder. Modern molecular data, however, suggested a closer relationship of beaver to the representatives of the mouse-related clade, but significant data from virtually homoplasy-free markers (for example retroposon insertions) for the exact position of the beaver have not been available. We derived a gross genome assembly from deposited genomic Illumina paired-end reads and extracted thousands of potential phylogenetically informative retroposon markers using the new bioinformatics coordinate extractor fastCOEX, enabling us to evaluate different hypotheses for the phylogenetic position of the beaver. Comparative results provided significant support for a clear relationship between beavers (Castoridae) and kangaroo rat-related species (Geomyoidea) (p < 0.0015, six markers, no conflicting data) within a significantly supported mouse-related clade (including Myodonta, Anomaluromorpha, and Castorimorpha) (p < 0.0015, six markers, no conflicting data).

Most of an organism’s phylogenetic history is fossilized in their heritable genomic material. Using data from genome sequencing projects, particularly informative regions of this material can be extracted in sufficient numbers to resolve the deepest history of speciation. Such regions contain many incidences of rare genomic changes, their most well-known representatives being retroposons, jumping elements that were reverse transcribed from RNAs and integrated almost randomly into other genomic locations over evolutionary periods of time. If the same retroposons are found in exactly the same places within the genomes of two species but not in others, we know the element was integrated in their common ancestor, indicating that they are closely related. Conflicts within these marker patterns (e.g., shared diagnostic insertions in distantly related species), are rare and, if present, were mostly caused by persistence of ancestral insertion polymorphism during speciation, which is why congruence among several different diagnostic loci is needed. Because rare genomic changes are frequent but phylogenetically informative ones rare, only large-scale screenings can provide statistically significant numbers of diagnostic markers. This is checked by customized statistical tests that evaluate whether the number of extracted diagnostic markers provides significant evidence of a given relationship and also whether potential conflicts exist among these markers^1^,^2^. Many relationships within the complex evolution of rodents were resolved by retroposon presence/absence screenings based mainly on the three available genomes of mouse, guinea pig, and ground squirrel, and subsequent PCR verification of potential diagnostic loci in other rodents^3^,^4^,^5^. However, in these studies the phylogenetic position of one of the most iconic rodents, the beaver (Castoridae), could not be resolved. While molecular sequence support for placing beaver together with kangaroo rat within the mouse-related clade, a group comprising Myodonta (e.g., mice, blind mole-rats, jerboas), Anomaluromorpha (scaly-tailed squirrels and springhares), and Castorimorpha (beavers, pocket gophers, kangaroo rats/mice), has accumulated^6^,^7^, so far only a single retroposon marker supported for placing the beaver inside this clade^5^.

Castoridae first appeared in North America at the end of the Eocene. They dispersed to Asia and Europe in the early Oligocene^8^,^9^, a time marked by a great faunal turnover, the *Grande Coupure* (about 33.5 Ma). In the early Miocene (about 24 Ma), beavers evolved the ability to swim and to cut trees, likely enabling them to adapt to increasingly harsh winters in Arctic latitudes^10^. The extant genus *Castor* probably originated in Eurasia^11^,^12^ with subsequent migration of the ancestors of *C. canadensis* to North America via the Bering Land Bridge in the late Myocene (~7.6–8 Ma)^13^.

Previous attempts to reconstruct the evolutionary history of the beaver based on retroposon presence/absence patterns were unsuccessful due to insufficient genomic information, motivating us to assemble a rough Eurasian beaver (*C. fiber*) genome from freely-accessible, short, paired-end Illumina reads^14^. These provided long enough sequences to analyze phylogenetically informative SINE retroposons (short interspersed elements – the most frequent retroposons active in rodents) embedded within them.

## Results

### fastCOEX screening of the assembled beaver genome

The contribution of sequence-independent presence/absence markers to the reconstruction of beaver phylogeny was previously restricted by the lack of enough beaver genomic information to find such rare genomic changes. Phylogenetically informative retroposon signals for rodents occupy ~200 to 300 nt of insertion loci. However, only genome-wide restriction site-associated DNA paired-end data (PE-RAD; both ends of the DNA-fragments are sequenced) were available for beaver. These were originally derived for population genetic studies of the Eurasian beaver^14^. Unfortunately, such sequence traces (~100 nt) are unsuitable for detecting phylogenetically informative retroposon insertion loci, most of which are more than 200 nt. Because the closest reference genomes of mouse, jerboa, or kangaroo rat are too distant or incomplete, we used a *de novo* assembly strategy for PE-RAD to derive 133,796 contigs of varying lengths (minimum 200 nt; average 545 nt; maximum 11,369 nt) from the available beaver trace sequences (8 samples; a total of 421 million reads; ~100 nt each). In so doing, we were able to reconstruct ~3% of the beaver genome.

**Figure 1:**
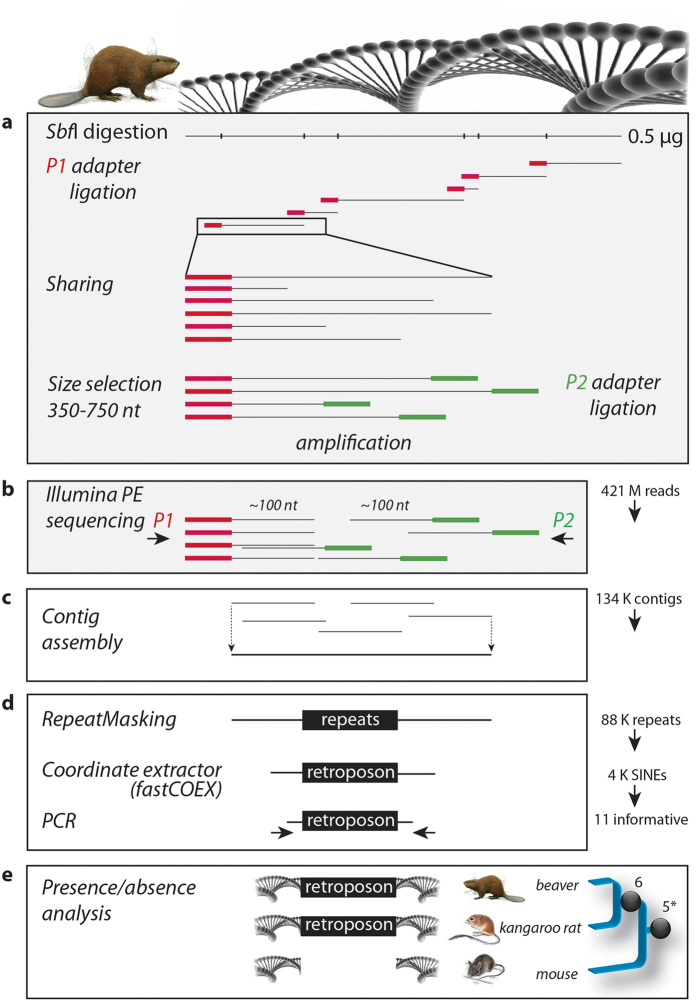
Snapshot of retroposon presence/absence analysis from Illumina paired-end reads (PE-RAD). (**a)** Library construction: genomic *Sbf*I digestion, P1 adapter ligation, random sharing (further shown for a selected single read), and size selection of 350–750 nt followed by the P2 adapter ligation and amplification steps. (**b)** Paired-end Illumina sequencing: the P1 and P2 sequencing primers generate shifted and partially overlapped ~100-nt reads. (**c**) The described PE-RAD sequences were taken from Senn *et al*.^14^ to build a gross genome assembly including ~134,000 fasta contigs >200 nt. (**d)** RepeatMasking revealed ~88,000 repeats and ~4,000 rodent-specific SINE loci. These loci were extracted from the fasta assembly using the coordinate extractor tool fastCOEX described in Materials and Methods. For control, the 11 phylogenetically informative loci were PCR-amplified/sequenced in beaver and comparatively sequenced in additional rodents. (**e)** Informative SINEs (black balls) are shared by beaver and kangaroo rat (6) or all species of the mouse-related clade (5 plus 1 additional previously published marker^5^ is denoted by *), indicating their close relationship. The in this study performed analyses are shown in white boxes. The rodent paintings were provided by Jón Baldur Hlíðberg.

Using a local version of the RepeatMasker, we extracted potential phylogenetically informative SINE markers from these 133,796 long contigs (Fig. 1). The assembled data and RepeatMasker table are available at http://retrogenomics.uni-muenster.de/tools/fastCOEX/download. A new web-based tool called fastCOEX was generated that uses the fasta long contig file to extract and trim selected potential phylogenetically informative retroposons and their flanking regions, based on their RepeatMasker-derived coordinate table. These extracted loci were then used for inter-species comparisons and PCR amplification for missing species to gain information about the phylogenetic placement of the beaver.

**Figure 2:**
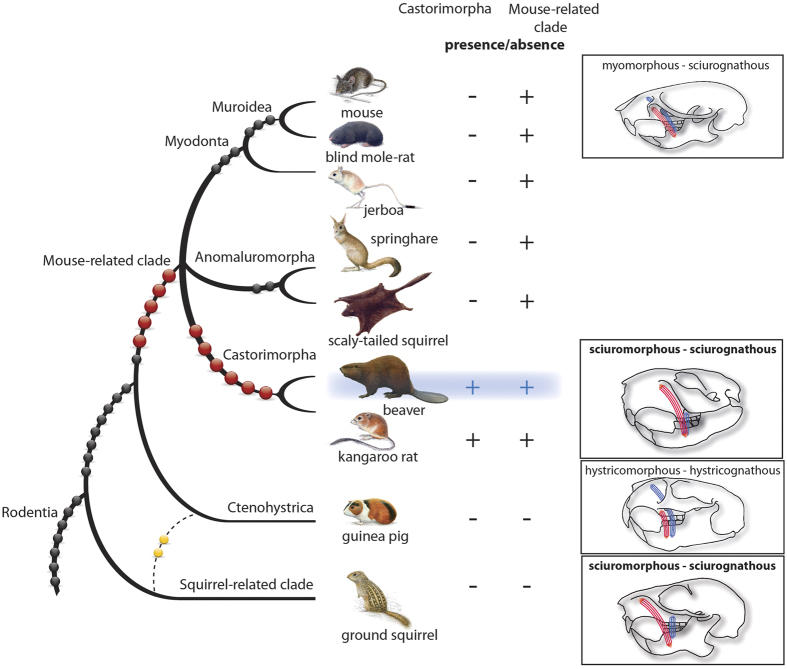
Reconstruction of the rodent phylogenetic relationships based on a gross beaver genome assembly and retroposon presence/absence screening (highlighted in blue). 3,780 potential phylogenetically informative retroposons were extracted from the beaver reference assembly and projected onto sequence information of other rodent genomes and onto PCR-amplified orthologs from Anomaluromorpha. These newly revealed markers are shown as enlarged red balls. Previously identified phylogenetically diagnostic retroposon markers are indicated by black and two conflicting yellow balls^5^. The two screening strategies and the resulting diagnostic presence/absence patterns are indicated for Castorimorpha and also the mouse-related clade. The myomorphous, sciurimorphous, and hystricomorphous zygomasseteric systems^18^,^19^ are illustrated to the right (blue and red lines show anterior parts of medial and lateral masseter, respectively; for details of zygomasseteric systems in rodents see Potapova^27^). The mandible types^20^ are noted: sciurognathous and hystricognathous. For the squirrel-related clade, only the zygomasseteric system of Sciuridae is presented. The rodent paintings were provided by Jón Baldur Hlíðberg.

To test the sister-group relationship between Castoridae and Geomyoidea (the Castorimorpha clade) to the exclusion of Myodonta and Anomaluromorpha against other possible relationships, we screened this *de novo* partial genome assembly of the beaver for SINE retroposons, and compared identified loci with those in the kangaroo rat (Geomyoidea) and mouse (Myodonta) computationally and with members of Anomaluromorpha experimentally (via PCR amplification of orthologous loci, see Materials and Methods). From the more than 13,000 embedded SINEs, we extracted 3,780 potentially informative ones via fastCOEX. Manual alignments and analyses of their presence or absence in orthologous regions of other species finally yielded six phylogenetically informative SINE markers that integrated into the genome of the common ancestor of Castorimorpha (see Supplementary Material) and none that suggested other relationships. Thus, they significantly support the union of Castoridae and Geomyoidea to the exclusion of all other lineages of the mouse-related clade (multi-directional derived significance value of p < 0.0015; [6 0 0] KKSC-test^2^; Fig. 2).

The search for SINE markers to resolve the immediate phylogenetic relatives of the beaver was combined with screenings for more distant relationships to other rodent lineages. This search revealed five new SINE insertions shared by the beaver and representatives from the mouse-related clade that were absent in the guinea pig (Ctenohystrica) and ground squirrel (squirrel-related clade), and no markers grouping the beaver with representatives of Ctenohystrica and the squirrel-related clade. These five combined with one previously found marker^5^ provide the first significant retroposon evidence (p < 0.0015; [6 0 0] KKSC-test) for the previously proposed mouse-related clade^15^,^16^, including the subclades Myodonta, Anomaluromorpha, and Castorimorpha.

### Additional multi-directional screenings

As the multi-directional KKSC insertion significance test^2^ ideally checks the significance of data by testing all possible hypotheses, we also performed additional multi-directional screenings of combined 2-way genome alignments supplemented by associated genome screenings to detect potential markers contradicting the Castorimorpha and mouse-related clade hypotheses. For the Castorimorpha clade we tested the following alternative tree topologies. Retroposon markers with pattern 1 – mouse(+)/beaver(+)/kangaroo rat(−)/outgroups(−) would indicate a close relationship between the mouse and the beaver to the exclusion of the kangaroo rat. However, all 16 potential phylogenetically informative markers identified for this pattern were rejected based on clear misalignments in the 2-way comparisons. Markers with pattern 2 – mouse(+)/kangaroo rat(+)/beaver(−)/outgroups(−) would indicate a close relationship of the mouse and the kangaroo rat to the exclusion of the beaver; but again, all 14 potential candidate loci were rejected due to alignment inaccuracies in the 2-way comparison. There were 26 candidate loci with retroposed elements supporting pattern 3 – kangaroo rat(+)/beaver(+)/mouse(−)/outgroups(−), of which three fulfilled all the criteria of informative markers and were also detected as informative markers in the exhaustive screening of the beaver assembled Illumina reads. The other three informative markers found during the fastCOEX screening were not detected in the additional multi-directional screening because of additional insertions of repetitive elements in the flanking regions of the corresponding mouse loci (exclusion criterion in automated 2-way screening). Thus, testing the alternative hypotheses of the phylogenetic position of the beaver did not reveal markers contradicting the Castorimorpha clade.

To test potential alternative hypotheses to the monophyly of the mouse-related clade, we used the same 2-way alignment sets to screen for the following presence(+)/absence(−) patterns. Twelve loci containing potential markers with pattern 1 – guinea pig(+)/kangaroo rat(+)/mouse(−)/outgroup(−) were all rejected due to detected misalignments in the 2-way comparisons. Thirteen loci containing potential markers with pattern 2 – guinea pig(+)/mouse(+)/kangaroo rat(−)/outgroup(−) were also all rejected because of misalignments in the 2-way comparisons. Thirty-three loci were identified with potential phylogenetically informative retroposon markers supporting pattern 3 – mouse(+)/kangaroo rat(+)/guinea pig(−)/outgroup(−). Eighteen of them were rejected due to misalignments, and 15 potentially supported the monophyly of the mouse-related clade. Four of these were also found in our fastCOEX screening; however, the beaver genomic assembly was incomplete in the regions containing the remaining 11 markers. One of the diagnostic markers from the fastCOEX screening and the previously published marker^5^ escaped detection in the multi-directional screening due to a large deletion and an additional element insertion, respectively, in the flanking regions of the mouse loci. Thus, testing the alternative hypotheses to the monophyly of the mouse-related clade did not reveal any conflicting markers.

## Discussion

Based on the zygomasseteric structure of their skulls (i.e., the arrangement of the masseteric musculature components in their jaws) and the configuration of their infraorbital foramina, beavers, pocket gophers, and kangaroo rats were once placed together with squirrels in the rodent suborder Sciuromorpha^17^. In contrast to the protrogomorphous, hystricomorphous, and myomorphous conditions, in the sciuromorphs the lateral masseter is shifted anterodorsally and originates on the rostrum and the widened root of the zygomatic arch, whereas the medial masseter does not pass through the reduced infraorbital foramen onto the rostrum^18^,^19^. This sciuromorphous zygomasseteric structure as well as the sciurognathous type of mandible^20^ were responsible for a more than 50-year-long phylogenetic association of Castoridae and Sciuridae^17^,^21^ (see Fig. 2 right panel sciuromorphous+ sciurognathous for beaver and squirrel). Interestingly, like beavers, members of the Geomyoidea clade (e.g., pocket gophers and kangaroo rats) also possess the sciuromorphous zygomasseteric condition^17^. Dental characters of early Tertiary rodents speak for only distant phylogenetic relationships among Sciuroidea, Castoroidea, and Geomyoidea, therefore supporting the idea that the sciuromorph characteristics evolved independently three times^22^. However, comparisons of molecular sequences have increasingly suggested a close relationship of Castoridae and Geomyoidea united as Castorimorpha and a phylogenetic affiliation of Castorimorpha with rodents of the mouse-related clade^15^,^16^,^23^,^24^,^25^,^26^. Therefore, in light of molecular data, the similarity of zygomasseteric types in Sciuridae and Castorimorpha is currently interpreted to be the result of parallelism^27^. The higher functional efficiency of advanced zygomasseteric types (sciuromorphous, hystricomorphous, myomorphous) is only apparent when compared with the more generalized protrogomorphous type^19^,^27^,^28^. A recent comparison of the advanced types among the squirrel (sciuromorphous), guinea pig (hystricomorphous), and rat (myomorphous) showed that the sciuromorphous muscle arrangement in squirrels emphasizes incisor gnawing versus the molar chewing emphasis in hystricomorphous guinea pigs^29^. Evolution of the sciuromorphous condition, albeit with its own individual features of skull morphology and jaw-closing muscles, enabled the beaver to become the most efficient gnawer among rodents^30^.

Based on a new approach of deriving a gross genome assembly from deposited genomic Illumina paired-end reads, we present six retroposon markers as highly significant evidence of the clade Castorimorpha (Castoridae + Geomyoidea) (p < 0.0015). This association was tentatively endorsed by Carleton and Musser in the *Mammalian Species of the World* 2005^31^, based mainly on molecular data^19^,^32^,^33^. However, at that time the Castorimorpha clade was still not generally accepted. For example, DeBry’s data^34^ support an unresolved trichotomy of Geomyoidea, Pedetidae (e.g., springhares), and Castoridae. More recent sequence data from four nuclear and two mitochondrial genes^4^, analysis of two mitochondrial genes, two nuclear exons, and four nuclear introns plus the effect of removing fast-evolving nucleotides^35^, a molecular supermatrix likelihood-based analysis of 26 nuclear loci^6^, and a partitioned coalescence analysis of Meredith’s 2011 data^7^ provide relatively strong support for Castorimorpha.

Veniaminova and colleagues^36^ performed a retroposon SINE B1 structural variation analysis and, based on just two probably homoplasious changes, challenged the monophyly of Castorimorpha, placing the beaver outside the mouse-related plus Ctenohystrica clades. In contrast, we performed multi-directional screenings of SINE presence/absence patterns and found strong, conflict-free, sequence-independent evidence for the Castorimorpha clade based on the 3% beaver genome assembly.

Furthermore, the mouse-related clade, once postulated by Huchon and colleagues^15^, and supported by one retroposon presence/absence marker in a later study^5^, is now significantly supported by five additional presence/absence retroposon insertions shared by Myodonta, Anomaluromorpha, and Castorimorpha, and no markers suggesting other relationships.

Unfortunately, as impressive as beavers are at modifying their local environment, they have been vulnerable to exploitation by humans especially in the nineteenth century. Hunting them for fur or the chemical substance Castoreum and the degradation of their natural habitats led to their near extinction. This reduced the Eurasian beaver population to only ~1,200 individuals^37^. A ban on hunting and reintroduction enabled partial recovery of the Eurasian beavers, which today number ~1.04 million individuals^38^. The beaver is still far from being the key faunal element it was during the European and Siberian Palaearctic era^39^, and therefore remains highly relevant for conservation.

## Conclusions

Solving prominent phylogenetic questions in evolutionary biology is one of the major targets of the era of genome analyses. Such phylogenomic investigations not only provide new insight into basic zoological/phylogenetic questions, but also create a solid basis for comparative genomic and functional studies (see for example the evolution of vocal learning in birds^40^). As exemplified in this study, data from Next-Generation Sequencing (NGS) techniques promise new advances in phylogenomics. With this in mind, we derived a gross genome assembly (~3% of the genome) of the beaver and extracted clear presence/absence patterns of SINE insertions to settle two of the most contentious issues of rodent phylogeny, the monophyly of Castorimorpha and the mouse-related clade. This provides a basis to address other still unresolved questions with SINE information as soon as more Illumina data are available: for example to test a potential sister relationship of Anomaluromorpha and Castorimorpha.

## Materials and Methods

### fastCOEX screening

From Traces to Contigs: The CLCbio Genomics Server 7.0.2 (QIAGEN, Hilden, Germany) was used along with the Bruijn graph^41^,^42^ for the eight available published paired-end (PE-RAD) Illumina sequence runs (ERR215688-91, ERR215693-96; https://trace.ncbi.nlm.nih.gov/Traces/study/?acc=ERP002076). The settings were (1) Mapping mode: simple contig sequences, (2) Automatic bubble size (bubbles in the graphical Bruijn structure are caused by inconsistencies in sequence reads due to SNP or sequencing errors. These regions must be resolved before the Bruijn graph structure is transferred to contigs, e.g., by choosing the sequence presenting the highest coverage of reads. If there are many systematic errors in sequences, bubbles will be very large and sequences will be split into shorter contigs. The default bubble size setting is suitable for short, high quality reads, e.g., as presented in the investigated PE-RAD data.), (3) Minimum contig length: 200, (4) Automatic word size (compiled read sub-sequences of a certain length are called words. Word-length is either short, e.g., 20 bases for small data sets or long, e.g., 27 bases for large data set and will be determined automatically), and (5) Performing scaffolding (for details see http://resources.qiagenbioinformatics.com/manuals). The resulting maximum contig length was 11,369 nt, with an average of 545 nt and 133,796 counts. The count for N25 was 708, for N50 664, and for N75 504 (for example the count for N25 is retrieved by summarizing the lengths of the biggest contigs until 25% of the total contig length is reached). The *de novo* assembly recovered ~3% of the beaver genome (~73 Mb of genomic information), took 24 h, and was performed with a UNIX server with 2 × Xeon 3.07 GHz, 16 cores, and 192 GB RAM.

Repeat detection in contigs: 133,796 contigs with sequences >200 nt were screened for SINEs in a RepeatMasker run (setting rodents for the repeat library) that yielded a total of >13,000 rodent-specific SINEs. These were extracted by a new bioinformatics fasta sequence coordinate extractor called fastCOEX that selects table-listed regions amenable for PCR verification. The web tool is available at http://retrogenomics.uni-muenster.de/tools/fastCOEX. We selected element families that were active in lineages leading to beaver, and elements embedded in beaver scaffolds with >50 nt to their left and right flanks in which to place PCR primers for experimental verification and computational screening of orthologs in other rodents, resulting in 3,780 SINEs consisting of 174 B1F, 136 PB1D7, 25 PB1D9, 724 PB1D10, 340 PB1D11, 232 SP-D-Geo, and 2149 SINE/ID elements (using default settings of fastCOEX). Each beaver locus, containing one of the mentioned SINEs, was manually aligned to the orthologous locus in the genomes of other rodents available from the NCBI database (date of access: May 2016) to derive presence/absence patterns (Supplementary Table S1). The manual alignments were generated with the assistance of NCBI Nucleotide BLAST (blastn, https://blast.ncbi.nlm.nih.gov/Blast.cgi. Option: Align two or more sequences). The presence of a given beaver SINE, e.g., in the kangaroo rat as a representative of the Geomyoidea (Dord_2.0; http://www.ncbi.nlm.nih.gov/genome/?term=Dipodomys), and its absence in all other rodents provided evidence for a Castoridae-Geomyoidea sister-group relationship, whereas shared SINEs in the beaver, kangaroo rat, jerboa, blind mole-rat, and mouse supported the mouse-related clade affiliation. We checked the orthology of elements, according to established criteria^2^, using the UCSC Genome Browser (https://genome.ucsc.edu) or available 2-way and multi-way genome alignments. All derived alignments of informative loci are provided as Supplementary Material.

Experimental verification of informative loci: We used standard PCR amplification and DNA sequencing protocols to fill necessary sequence information for the phylogeny of the South African springhare (*Pedetes capensis*, sample voucher: T-3277, Université de Montpellier II), and a scaly-tailed squirrel (*Anomalurus* sp., sample voucher: T-1787, Université de Montpellier II), and to experimentally verify the computationally assembled beaver loci (the list of designed PCR primers is given in Supplementary Table S2). All experimentally derived sequences have been deposited in GenBank (accession numbers KY230357–230373).

### Additional multi-directional screenings

To perform 2-way genome screenings for testing potential alternative tree topologies we used a combination of computational screenings to check for potential support of different tree topologies using the following 2-way alignments:

mouse/kangaroo rat (http://hgdownload.soe.ucsc.edu/goldenPath/mm10/vsDipOrd1/axtNet/);

mouse/guinea pig (http://hgdownload.soe.ucsc.edu/goldenPath/mm10/vsCavPor3/axtNet/);

mouse/ground squirrel (http://hgdownload.soe.ucsc.edu/goldenPath/mm10/vsSpeTri2/axtNet/);

guinea pig/mouse (http://hgdownload.soe.ucsc.edu/goldenPath/cavPor3/vsMm10/);

kangaroo rat/mouse (http://hgdownload.soe.ucsc.edu/goldenPath/dipOrd1/vsMm10/).

Furthermore we used the following available genomes:

mouse http://hgdownload.soe.ucsc.edu/goldenPath/mm10/chromosomes/;

kangaroo rat http://hgdownload.soe.ucsc.edu/goldenPath/dipOrd1/bigZips/dipOrd1.fa.gz;

guinea pig http://hgdownload.soe.ucsc.edu/goldenPath/cavPor3/bigZips/cavPor3.fa.gz;

ground squirrel http://hgdownload.soe.ucsc.edu/goldenPath/speTri2/bigZips/speTri2.fa.gz,;

and partially assembled genome of the beaver presented in this study.

We downloaded and used the following RepeatMasker reports:

mouse http://hgdownload.soe.ucsc.edu/goldenPath/mm10/bigZips/chromOut.tar.gz;

kangaroo rat http://hgdownload.soe.ucsc.edu/goldenPath/dipOrd1/bigZips/dipOrd1.fa.out.gz;

guinea pig http://hgdownload.soe.ucsc.edu/goldenPath/cavPor3/bigZips/cavPor3.fa.out.gz.

We extracted rodent SINE element coordinates from RepeatMasker reports and mapped them to the corresponding reference sequence of the respective 2-way genome AXT alignments. In a pre-analysis, a diagnostic absence of a SINE in given species of the 2-way alignment was only selected if >70% of the SINE elements were adjusted to a gap in the second species. Only SINE loci free of additional repetitive elements in their 50 nt flanks were extracted and the maximum allowed overlap of SINEs and flanks was 10 nt. Combinations of 2-way alignments presenting the absence state of SINEs in the ground squirrel were used to define the plesiomorphic condition.

We used different combinations of 2-way alignments (Mm10 vs. DipOrd1, Mm10 vs. CavPor3, Mm10 vs. SpeTri2, dipOrd1 vs. Mm10) and reciprocal genome blasting (for the beaver) to screen for retroposed elements supporting the following potential presence(+)/absence(−) patterns:

1. mouse(+)/beaver(+)/kangaroo rat(−)/guinea pig(−)/ground squirrel(−)
2. mouse(+)/kangaroo rat(+)/beaver(−)/guinea pig(−)/ground squirrel(−)
3. kangaroo rat(+)/beaver(+)/mouse(−)/guinea pig(−)/ground squirrel(−)

To test potential alternative hypotheses to the monophyly of the mouse-related clade we used the same 2-way alignment sets used in the previous screening to search for the following presence(+)/absence(−) patterns:

1. guinea pig(+)/kangaroo rat(+)/mouse(−)/ground squirrel(−)
2. guinea pig(+)/mouse(+)/kangaroo rat(−)/ground squirrel(−)
3. mouse(+)/kangaroo rat(+)/guinea pig(−)/ground squirrel(−)

Potential candidates were extracted and realigned manually.

### Statistical evaluation of phylogenetic informative markers

The significance of the derived phylogenetic position for beaver was tested using the KKSC test^2^ located at http://retrogenomics.uni-muenster.de:3838/KKSC_significance_test/. The KKSC test is based on the assumption that the time of insertion of retroelements and their fixation or loss in a population are critically different depending on the effective population size, and might overlap speciation events. We used the Wright-Fischer coalescent model^43^,^44^, Markov processes, and a diffusion approximation to describe the probability of fixation of elements^2^. All possible phylogenetic tree topologies are tested and processes such as incomplete lineage sorting and ancestral hybridization can be differentiated. Performing multi-directional screenings of combined 2-way genome alignments supplemented by associated individual genome screenings enabled us to apply the multi-directional KKSC insertion significance test to evaluate the significance of the Castorimorpha and mouse-related clade hypotheses.

## Additional Information

**How to cite this article:** Doronina, L. *et al*. The Beaver’s Phylogenetic Lineage *Illumina*ted by Retroposon Reads. *Sci. Rep.* **7**, 43562; doi: 10.1038/srep43562 (2017).

**Publisher's note:** Springer Nature remains neutral with regard to jurisdictional claims in published maps and institutional affiliations.

## Supplementary Material

Supplementary Information
