# Supplementary material for: The Beaver’s Phylogenetic Lineage Illuminated by Retroposon Reads
Source: Sci Rep. 2017 Mar 3;7:43562. doi: 10.1038/srep43562 (PMC5335264; doi:10.1038/srep43562)
Supplement: Supplementary Information [file srep43562-s1.pdf]

Supplementary Information for "The Beaver's Phylogenetic Lineage *Illumina*ted by Retroposon Reads"

Liliya Doronina[1], Andreas Matzke[1], Gennady Churakov[1,2], Monika Stoll[3], Andreas Huge[3], Jürgen Schmitz[1]

[1]Institute of Experimental Pathology (ZMBE), University of Münster, Münster, Germany

[2]Institute for Evolution and Biodiversity, University of Münster, Münster, Germany

[3]Core Facility Genomics, Medical Faculty, University of Münster, Münster, Germany

Table of content:

Supplementary Table S1	Presence/absence table of all informative retroposon markers
Supplementary Table S2	List of PCR primers
Supplementary Material	List of markers

Supplementary Table S1

Presence/absence table of informative retroposon markers

(1) indicates the presence of retroposed elements, (0) indicates its absence, (?) denotes absence of sequence information. Marker IDs correspond to those of the contigs containing these markers.

The Castorimorpha clade

Species name	Marker ID					
	42657	76528	64100	97783	62793	69458
Mus musculus	0	0	0	0	0	0
Rattus norvegicus	0	0	0	0	0	0
Apodemus sylvaticus	0	0	0	?	0	0
Peromyscus maniculatus	0	0	0	?	0	0
Microtus agrestis	0	0	0	?	0	0
Nannospalax galili	0	0	0	0	0	0
Jaculus jaculus	0	0	0	0	0	?
Pedetes capensis	?	0	?	?	?	0
Anomalurus sp.	0	0	0	0	0	?
Castor fiber	1	1	1	1	1	1
Dipodomys ordii	1	1	1	1	1	1
Heteromys gaumeri	?	1	?	?	?	?
Chaetodipus baileyi	1	1	?	?	?	?
Cavia porcellus	0	0	0	0	0	0
Ictidomys tridecemlineatus	0	0	0	0	0	0

The mouse-related clade

Species name	Marker ID				
	20039	16758	93865	25386	7855
Mus musculus	1	1	1	1	1
Rattus norvegicus	1	1	1	1	1
Apodemus sylvaticus	1	1	1	1	1
Cricetulus griseus	?	1	1	1	1
Mesocricetus auratus	?	1	1	1	1
Peromyscus maniculatus	1	1	1	1	1
Microtus agrestis	1	1	1	1	1
Nannospalax galili	1	1	1	1	1
Jaculus jaculus	1	1	1	1	1
Pedetes capensis	?	?	?	?	?
Anomalurus sp.	1	?	?	?	?
Castor fiber	1	1	1	1	1
Dipodomys ordii	1	1	1	1	1
Cavia porcellus	0	0	0	0	0
Heterocephalus glaber	0	0	0	0	0
Ictidomys tridecemlineatus	0	0	0	0	0
Marmota marmota	0	0	0	0	0

Supplementary Table S2

List of PCR primers

Locus names are based on the marker IDs in Table S1, (f) indicates forward primer, (r) – reverse primer.

Locus name	Primer sequence	Tm temperature, °C
42657f1	GCCTGGCTTGCAGYTGACCTT	62.8
42657r1	GCTCTCTGCCTTCTGCCTCTSAT	64.2
42657f2	TGAGGACATGTCCCTGTRTGACCT	63.6
42657r2	GGAAACAGCTCTCTGCCTTCTGC	64.2
76528f	GCACCAGCTCCCCRACACC	64.2
76528r	GGAAGCAGAATAGGAATCTCCATTGAG	63.4
64100f	GAAATTTCCTGRTGGTTTTAGAAGAGC	61.2
64100r	TCCTCCCACACGTGGATGATGT	62.1
97783f	GGACCTTTTRAACTGCTCTGATTGAG	62.4
97783r	CTGACCTCCTCCCAGGCTTGTC	65.8
62793f	TCATTTGCTCCACTRTGAGGAAA	57.5
62793r	CCGAGCACCAGCAGTTTGATG	61.8
69458f1	GAAGGGCTAGCACAATGTGTTCTAAG	63.2
69458r1	CAATTCACCTGCCTGGACTTG	59.8
69458f2	GGCACATGATAGACACCTTGTAAATG	61.6
69458r2	AGTGCCTCAGAAATGTTTGATGATC	59.7
20039f	AGGCCTCTCTGGATTCTGCTCAG	64.2
20039r	GCATCAGTTGCCATTCCAGTGAG	62.4
16758f	CACAGAATTTCGGCTGGGCATAG	59.5
16758r	GAGCAGTTTGACAAGAAGTACCTGAG	60.1
93865f	TACTACCTCCCATATGCACTGACTGAC	65.0
93865r	GTTAATTGCTGGTTCCTGGCTCTGA	63.0
25386f	CTTTCTTGATGCTTTTGGGTTCTRTC	60.9
25386r	CTTCCTTTCTTGGTGCTCTTTGG	60.6
7855f	TGCAAGAAGGGGTGGAATAGATG	60.6
7855r	CGCAAATGTTTATGGAATGGGATAG	59.7

Supplementary Material

List of markers

Castorimorpha clade

Marker 42657

```
>PB1D10
--------------------------------------------------------------------------------
-------TTTTGTTTTTTT------GAGACAGGGTC----TTGCTATGT-AGCCCAGGCTGGCCTTGAACTCACGATCCT
CCT----GCCTCAGCCTCCCGAGTGCTGGGATTACAGGCGTGCGCCACCACGCCCGGCT---------------------
--------------------------------------------------------------------------------
--------------------------------------------------------------------------------
--------------------------------------------------------------------------------
--------------------------------------------------------------------------------
---------------------------------------------------
>Mus musculus
TGATT---TGTCTAGCATATGCAGCTGACCTT---CTGAGGACACA-ATGCCTGTACGAGCTGGCTCTTT--TAAG--GT
--------------------------------------------------------------------------------
--------------------------------------------------------------------------CAGAG-
----AAAGAAG--CCGTT-TCATCCTCAGATTCTCCCTCAGCCCAGGATCCC-AGCAGAGTCCAAACTGTGAAGTCTGGC
--------------------------------------------------TCCAGCC-----TGCTGACCACCATCCGTG
GTCCTGTGGCCTTGCTGGGTTGCCCA---TTGTCTAGCCAGGCCAGCCCCTCAGGCCTCAAGCTCACCCTGTCTGT-GCC
C-GCTCAGCCT-GGCCAGCTGGCCGTCCAACCCC-----------------AGCCCCTCCTGC-CACCGTG---CTTTCG
CCT--------CAAGCTC-----CGTTAA-TTTTCATGGCCTCCATCAGAG
>Rattus norvegicus
TGATT---TGTCTAGCATATGCAGCTGACCTT---CTGAGGACACA-ATGCTTGTATGAGCTGGCTTTTT--TAAG--GT
--------------------------------------------------------------------------------
--------------------------------------------------------------------------CAGAG-
----AAAGAAA--CTGTTCTCATCCTCAGATTCTCCCTTAGCCCCGGATCTT-AGGAGAGTCCAAACTGTGAAGCTTGGC
---------------------TCC-CCA---AGGATGACTAGA--CTGGCTCCAGCC-----TGCTGACCACCATCCGTG
ACCCTGTGGCCTTGTTGGGTTGCCAG---TCGTCTGTCCAGGCAAGCCCCTCAGGCCTCAAGCTTACCCTGCTGGT-GCC
CA-TTCAGTCT-GGCCAGCTGGCCAGCCAGCCCC-----------------AGCCCCTCCTGC-CACAGTG---CTTTCG
CCT--------CAAGCTC-----CGTTAA-TTTTCATGGCCTCCATCAGAG
>Apodemus sylvaticus
TGATT---TGTCTAGCATATGCAGCTGGCCTT---CTGAGGACACA-ATGCCTGTATGAGATGGCTCCTT--TAAG--CT
--------------------------------------------------------------------------------
--------------------------------------------------------------------------CAGAG-
----AAAGAAA--CTGTTCTCATCCTCAGAGTCTCCCTCAGCCCAGGAACCC-AGGAGAGTCCAAACTGTGAAGTCTGGC
---------------------TCC-CCA---AGGAT---------CTAGCTTCAGCC-----TGCTGACCACCACCCGTG
ACCCTGTGGCCTTGCTGGGTTGCCAA---TTGTCTGACCAGGCCAGCCCCTCAGGCCTCAAACGTGCCCTGCTGGG-GTC
C-GCTCAGCCT-GGC-AGCTGGCCGTCCAGCCCC-----------------AGCCCCTCCTGC-CACAGTG---CTTTCG
CCT--------CAAGCTC-----CGTTAA-TTTTCATGGCCTCCATCAGAG
>Peromyscus maniculatus
TGATTT-CTGTCTAGCATATG--------------CTGAGGACACACGTGCCTGTATGACCTGGCTCTTT--TAAG--GT
--------------------------------------------------------------------------------
--------------------------------------------------------------------------CAGAG-
----AAAGAAA--CTAGTCTCATCCTCAGATTCTCCCTGGGTTCGGGATCCC-AGGATAGTCCAAACTGTGAAGTCTGAC
---------------------TCCCTTA---AGAGTAGCTGGA--CTTGCTCCAGCC-----TGCTGACCACCATCCGTC
ACCCTGTGGCCTTGCTGGGTTGCCAA---TTGTCTGGCCAGACCAGCCCCTCAGGCCTGAAGCTTGCCCTGCTGGT-GCC
--GCTCGGCTT-GGCCAGCTGGCTGTCCAGCCCG-----------------AGCCCCTCCTGC-CACAGTGTTTACGTCT
CTCTTTCGCCTCAAGCTC-----TGTTAATTTTTCATGGCCTCCATCAGAG
>Microtus agrestis
TGACT--CTGTCTAGCATATGCTGCTAGCCTT---CTGAGGACACT--TGCCTGTGTGACCTGGCTCTTT--TAAG--GT
--------------------------------------------------------------------------------
--------------------------------------------------------------------------CAGAG-
----AAAGAAA--CCATTCTCATCCTCAGGATCTCCCTGGGTTCAGGATCCC-AGGATAGTCCAAACTGGGAACTCTGAC
---------------------TCCC-TA---AGGGTAGCTGGA--CTTGCACCAGCC-----TTCTGACCACCATCCATG
ACCCCGTGGCTTTGCTGG-------------------TCATACCGACCCCTCAGGCCTCAAGCTTGGCCTGCTGGT-GCT
--GCTCAGCTT-GGCCAGCTGGCCGTGCAGCCCT-----------------GGCCTCTCCTGC-CACAGTGTTTTCATCT
CTCTTTAGCCTCAAGCTC-----TGTTAATTTTTCATGGCTTCCATCAGAG
>Nannospalax galili
TGTTT--CTGCCTGGCC--TGTAGCTGAC------CTGAGGACGCA--TCCCTGCATGACCTGGCTCTTT--TAAG--GT
--------------------------------------------------------------------------------
--------------------------------------------------------------------------CAGAG-
----AAAGAAT--CTGTTCTCATCCTCAGAGTCTCCATGGGCTAGAGATCCC-AGGAAGGTCTAGACTG--AAATCTGTC
---------------------TCCC-CAGGGGGTGTAACCAGG--TTTGCTGCAGCC-----TGCTGACCACCATTCATG
ACCCTGTGGCCCTGCTAGATGGCCAA--------------GGCCAGCCCCTCCTGCCCGAAGCTTGCCCTGATGGT-GCC
CA-TTCAGCTT-AGCCAGCTGGCCATACAAAACCTCCCCCCGCCCCCTGCCTTCCCCTC--AC-CACAGTGTTTCCTTCT
CTTTTCCTCTTTCATGGT------GTTAATTTTTCATGGCCTCCATCAGAG
>Jaculus jaculus
CAATTG-CTGCCTGGCC--TGCAGCTGACCTT--CCCAGGGAC--ACGTCCTTGTGTGACCTGGCTCCCT--TAAC--AG
--------------------------------------------------------------------------------
--------------------------------------------------------------------------AAGAG-
```

----AAAGAGGCTCTATTCTTGTCCTTGGATTCTCCATGG-------ATTC--GGGAGGGTCTGGCCTGTATAATGTGGC
---------------------TCCT-CA---GAGGAAACGGGG--TTTACTCCAGCCCACCCAGCTGACCATCATCCATG
ACCCTGTGGCCTTGCTGGGTG---AGATGTTGTGTGGCTGGGCCAACCCCTCGGGCTCCAGGCTTGCCTTGCTGGT-GCC
CA-CTCAGCTC-GGCCAGCTGGCCATGCAGCCCT-----------------CACCCCTCCCGC-GGTCATGTTTCCTTCT
CTCTTTG-CCTTGCGCTG-----TGTTAATATTTCATGGCCTCCATCAGAG
>Anomalurus sp.
----------------------------------CCCGAGGAC--ATGTCCCTGTGCGACCTGGCTCT----TAAG--GG

--CAGAG-
----AAAGAA--TATATTCTCATCCTCAGATTTCCCACGGTCTCGAGACCCC----------------------------
---------------------------A---GGGGTAGCTGGC--TTTGCTGCAGCC-CCCAGGCTGACCACCATCCACG
AGCCCCTGCCTCTGCTAGGTGGTCAG--
------------------CTGGCCGTCACACTCC-----------------CACAGCCCTCCACCGCCATG--TCCCTCT
CTCCTGGCCCGCGTGCTG-----TGGGAATTTTTCCTGGCCTCC-------
>Castor fiber
AGAGTTTCTGCCTGGCT--TGCAGTTGACCTT--TCTGAGGAC--ATGTCCCTGTATGACCTGACTCTCTCTTTTCTGAG
GTTTTTTTTGTGTTTTTTTTTTTTTGAGACAGAGTC------ACTGTGT-AGCCCAGGCTAACCCTGAACTCAGAATCCT
TCT--G-GCCTCTGCTTCCTGAGTGCTGGGATCACAGGCATGTACTACCTTGCCTAGCTTACCTGA-CTCTC--AAGGGC
AGAGCAAGA-----TATTCTTGTCCTCAGATTCTCCATGGACTAGAGACCCC-AGGAAAGTCAAGACTATATATTCTGCC
CCCGACCCTGCCCCCCCGCCCACCACCA--TGGACT-GTTGGG--TTTCCCCCAGACCCCCAGGCTAACCACCATCCACA
CCCCTGTGGCCCTGCTGGGTGGTCAG---TTTTCTGATCAGGCCAGTCCCTGGGGCTCCAAGCTTGCACAGCTGGT-GTT
C-CCTCAGCCTAGGCCAGCTGGTCATC------------------------CATACCCCACCCGCCACGTGTTTCTTTCT
CTTTCTCTGTGTGCTGTA-----T--TCATTTTTCATGGCCTCCATGAGAG
>Dipodomys ordii
GGATCT-CAGCTCAGTC--TGCAGCTGACCTT--TCCAAGGGC--ATGTCCCTATGTGATCTAGCTCTCT--TGTC--AG
--------TGTGTTCGTTTCTGTT-GAGGCAGAGTCAGTCTCACTATGC-AGCTCAGGCTGGCCTTGAATTCACGATCTT
CCT--ATGTTGCTGTCTTTTGACCG-TAGGATCACAGGCATGTGCCACCT-ACCTGGTTTAC-TAGGCTCTCTTAAAGGC
A-ACAAAGAG--TATATTCTTGTC---ATATTCTCCATGGACTCCAGATCCC-AAGAAGGTCAGGACTGTTTAATCTAGC
---------------------TCCCCAA---GGGGTCGTTGAA--TTTATTCCAATCCCCCCGGGCGAGC-----CAACA
ACTCTTTACCCCTGCTGGGTGGT-AG---TGGTCTGGCTA---------CTTGGGTTCCAAGCTTCCTTAGCTGGT-ACC
CACTTGGGTTCGGCCAGCTGGTCATCTGGCTCC------------------CACGCCCCTCCCTCCA--CGTTTCCTTCT
CTCTTTCTCTGCATGCTA-----TGTTTATTTTTCATAGCCTCCATGAGAG
>Chaetodipus baileyi
--AGCTCTTTT-TGCT--GT
-----------GTTTGTTTCTGTT-GAGGCGGAGTC----TCCCTGTGTAAGCTCAGGCTGGCCTTGAATTCGCAATCTT
CCTGCGTGGCTCTGCCTTTTGGGCG-TAGGATCATAGACAAGTGCCACCT-ACCTGGTTTTC-CAGCCTCCCTGAAAGGC
A-ACAAAGAG--TATGTTC--GCCATTATATTCTCCATGGACTCCAGACCCC-CAGAAGGTCAGGACTGTTTTATCTAGC
---------------------TCCCCAA---GGGGTCTTTGGA--TTTACCCCTGTCTCCCTTGGTGGG----GT-GACC
AGTCGTTACCCCTGCTGGGTGGT-AG---AGGTCTGGCTG---------CTTGGGCTGCAAGTTCACATAGCTGGC-ACC
CACTCTGGTGCTGGCCAGCTAGCTAGTCATCAGG-----------CTSCCGCACCCCTCCCTCCACA--TGTTTCCTTCT
CT-TTTCTCTGCATGCCC-----TGTTTATTTTTCATAGCCTCCATGAGAG
>Cavia porcellus
TGAGTTT---CCTGGCC--TGGTGTTGACCTTGACCCGAGAGC--ATGTCCCTGTGTGACCTGGCTGTCT--TCCA--GG

--TAGAG-
-----TGGAG--TATTTCCCTGCCCTCAGATTCTCCA-GGG-TC----------------TCAGTACTGTGTAATCCAGT
CCTGACTGTATAATCCTTGTCCCCCGAG---GGGAGTGCTGGGGCTGTGCCCCACTCC---TGGATGACCAC-ATCCGAG
ACACAGTGGCAGCACTGCGTGGTCAG---ATGTGTGGCCTGGCCAGTT-CTTGGGCTCCAGGCTTGTCCAGCTGGTCATT
CACCTGA--CAATCCTCCTAC-CCTGGTGTTTA---CT
C-CTCTCTGCTTGCTGTG-----T--TAATTTTTTATGGCTTTCATCAGAG
>Ictidomys tridecemlineatus
CGAGTTTCTGCCTGGTC--TGTAGTTGACCTT--CCTGAGGAC--ATGTCCTTGTGTAACCTGGCTCTCT--TAAG--GG

--CAGAG-
----AAAGAGA-ACCTATCCTGTCCTCAGATTCTCCAGGGGCTTGAGACCCCCAGGAAGGTCCCGATTG--TAACCTAGC
---------------------TCCTCCA--GGGGAGT--TGAG--TTCACTCCAGATCCC-AGGCTGACCTCCATCCTGA
ACCCTGTGGCCCTGAGGGGTGGTCAG---TTGTCTGGCTGAGCCAGTC-CTTTGGCTCCAGCCTTGT-------------
--------------CCAGCTGATCCTCCAGCCCT-----------------GACCCCTCC-GC-CCCAGTGTTTCCTTCT
CTCTCTCCTCTCTGTGTGTGCAGTGTTAATTTCTCATGGCCTCCATCAGAG

Marker 76528

>B1F1

----------------------------TTTTGTTTTTTTGAGACAGGGTCTCACTATGTA-GCCCTGGCTGGCCT-GGA
ACTCACTATGTAGCCCAGGCTG-GCCTCGAACTCACGGCGATCCT-------CCTGCCTCAGCCTCCCGAGTGCTGGGAT
TACAGGCGTGTGCCACCACGCCCGGCT---

>PB1D10

----------------------------TTTTGTTTTTTTGAGACAGGGT------------------------------
-CTTGCTATGTAGCCCAGGCTG-GCCTTGAACTCAC---GATCCT-------CCTGCCTCAGCCTCCCGAGTGCTGGGAT
TACAGGCGTGCGCCACCACGCCCGGCT---

>Mus musculus
CCCTTCCTCTGCAG-GT-----GGAGCCCACCGCCACG-CTGGCTGGCTGACT-------------------------TG
AGTGT-TTCTTGCCTTTGCTTCCACTT-GTGTCTGTGCAGGATGATAGCAAG--GAGGACTTTGGGGTGGGG----TGG-
-GGGAAGACCAGGCTAAGAT----GGGGTGGCAATGTCAA-GG-------------------------------------
----------GGTAGCCTGTTG----TGAGGGAG--
---GGCCTGCT-GTTCTGTG----

--GATAATCTTCATATAC-TCCGAGTC-TAGTCCCAGA
GAG---------TGAGCTTT-CTGGAAGCTCAGGGTTGGGTTTCCCT----CCTGTCCCCCATCAGA-GGGCCC-TGTGC
TTTTT--CTGATTCTCTCTCTCTCTGCCCTCGAA-----AG-GCTCTGGCGGCTCTTTCAATCTCTCAGGATCCGGAGAC
ATGTTTCTGGGGATGCCCGGG
>Rattus norvegicus
CCCTTCCTCTGCAG-GT-----ACAGCTCACTGCCGCT-CTGGCTGGCTGCTT-------------------------TG
CCCAT-TTGCTGCCTTTGCTTCCACTT-TTGTCCATGC-----------------AGGACTTTGGGGGGG------TG--
---AAAGACCAGGCTAAGAC----GGGGTGGTGATGTCAA-GG-----------------------------GCCTT---
---AAG--TGGGTAGCCAGTTCACGTTCACGAAT--
---AGCCTG----TCCTGAG----

----------------------------GGTGGG----CCTGTGGATAG------------TCTGCATA-TGCTTCCTGA
G-----------TGAGCTTT-CTGGAAGCCAAGGGTTGGATTTACCT----CCTGTCCCTCATC--AGGGTGCCCTGTGC
TTTTTCTCTGATTCTC----TCTCTGCCCTCCCA-----AG-GCTCTGGCGGCTCTTTCAATCTCTCAGGATCCGGAGAC
ATGTTTCTGGGGATGCCCGGG
>Apodemus sylvaticus
CCCTTCCTCTGCAG-GT-----GGAGCCCGCTGCCACG-CTGGCCG----------------------------------
-------------CTCTGCTTCCGCTT-CTGACCATGTGGGGTGACAGCAAG--GAGGTCTTTGGGGGGCTGGGGGTGAG
GGGGAAGACCAGGCTAAGAT----GGGGCGGTGATGTCAG-GGG------------------------------------
---CCT--TAGGTGGGTAAGCCAG-TTCACAAGT--
---AGCCTG----TTCTGAGGGT-

---------------------------GGGCCTGCTGTTCTGTGGG----------------------------------
------------TAATCTT--CTGGAAGCTAAGGGCTGGGGTTACCT----CCTGTCCCCCACCCAAGGGT-CC-TGGGC
TTTTTCTCTGATTCTC----TCTCTGCCCTCCCA-----AG-GCTCTGGCAGCTCTTTCAATCTCTCAGGATCCGGAGAC
ATGTTTCTGGGGATGCCCGGA
>Peromyscus maniculatus
CCCCTCCTCTGCAG-GT-----ACAGCCCACTGCCACT-CTAGCTGGCTGCTT-------------------------TG
CATGC-TTCCTGTCT-------------------ATGCAAGATGACAGCAAA--GAGGACTTTTTGGGG-------TG--
-AAAGAGACCA-------------GGGGTAATGATGTCAG-GGG------GAGGGGCCTTTA------------------
-----G--TGGGTGAGCTGGCGGGGTTCACAAGT--
---AGCCTG----TTCTGAGGGT-

---------------------------GTGTGTGCTGATCTGTGTGCGA------------TCCGTGTG-CATTCCCAGA
G-----------TTAGCTTCCCTGGAAGCTAAGGT-TGTGTTCATCTCCTTCCTGTCTACCGTCCGACGGTCCCCTGTAC
-TTTCCTCTAATTCTC----TCAATGCTCTCCCA-----AG-GCTCTGGCGGCTCTTTCAATCTCTCAGGATCCGGAGAC
ATGTTTCTGGGGATGCCCGGG
>Microtus agrestis
TCCTTCCTCTGCAG-GT-----GGAGCCCACGGCCACT-CTAGCTGGCTGCTT-------------------------TG
CATGA-TTCT------------------------ATGCAAGATGATAGCAAA--GAGGACGTTTTGGGG-------TG--
-AAAGAGACCAGGGGT--

```
-----G--TTGATGTCAAGGGGAGGGTCCCAGAT----------------------------------------------
-----------------------------------------------------------AGCCTG----TTCCGGGGGT-
--------------------------------------------------------------------------------
--------------------------------------------------------------------------------
---------------------------GGGACTGCTGTTCTGTGGGTAATTTGTATGTAA-TCCATGTG-CATTCCCAGA
G-----------TTAGCTTT-CTGGGAGCTAAGGGTTGCGTTCGTCT----CCTGTCTGCAGTCAGATGATCCCCTGTGC
-TTTTCTCTGATTCCC--TCTCTCTGCTCTTGCA-----AG-GCTCTGGCGGCTCTTTCAACCTCTCAGGATCCGGAGAC
ATGTTTCTGGGGATGCCCGGG
>Nannospalax galili
CCCTTCCTCTGCAG-GT-----GGAGCCCACTGCCATT-CTGGCTGGCTGTGTTTCTTTTGTTTTGTTTTGTTATTTTTG
CATT--TTCCTGCGTTTGTTCCCATTTCCTGTCTGTGCAGGATTACAGCAGA--GAGGACTTTG-GGGG-------TAA-
-AAAGGGGCCAAGCTGAGAT-GGTGTGATGTCAAGGACACAGGCCAGGGGAGACTTGGGAATAGCC--TGTTGTAA-GCC
TGGCA---TGGTGGTGCACTCTTAGTCCCAGCACTCAGGAGGCCGAGGCAGGCGGATCTCTGTGAGTTCGAGGCCATCCT
GGTCTATGTAGTGAGTTCCAGGACAGCTGGGGCTACAGGGAGACCCTGTCACAAAAAAGAAATTGCCTGTTCTGAGTGT-
--------------------------------------------------------------------------------
--------------------------------------------------------------------------------
---------------------------TGGA---CTGTTTTGTGGATAATTGATGTGTAA-TCCACACA-TATCCCCGGT
GAGTTAGCTTAGAGAGCTCT-CTGGAAGCTAGGAGTTGTGTTTATGG----CCTGTCTACCATCACAAGGTCCCCTGGGC
TTTTCC-CTCATCTCT---CTCTGT-CTCTCCCA-----AG-GCTCTGGTGGCTCTTTCAATCTCTCAGGATCCGGAGAC
ATGTTTCTGGGAATGCCTGGG
>Jaculus jaculus
CCCTTCCTCTGCAG-GT-----GGAGCCGACTGCCACC-CTGGCTAACTGTTT-------------------------TT
CACGC-TTCCTGCTTTTGTTCCCACTTCCTGTCTAGGCAGGATTATGGCAGA--GAGAACTTTTAGGGG-------TG--
-AAAGGGACCAAGCTAAGAT---GGGGGTGATGATGTCAG-GGATTGAGGGCTCTCCAAG----------TTGCTCTGCC
TGCCAG--TGAGTGGCTAGGGCAGGCTCGGGAAT----------------------------------------------
-----------------------------------------------------------AAACTGCTCTTTCCCAGGGT-
--------------------------------------------------------------------------------
--------------------------------------------------------------------------------
---------------------------GGGA---CTGTTCTGTGGGTAGTTTGTATGTAA-TCCATGTA-TGTCCCCAGA
GAACTAGCTTAGAGGGCTTT-CTGGAGC-------TTCT-CTTATCT-----------GCCATCTCAGGGCCCCTTGTAT
TTTTCC-CTAATTCCC----TCTGTGCTCTCTCA-----AG-GCTCTGGCGGCTCTTTCAATCTCTCAGGATCCGGAGAC
ATGTTTCTGGGAATGCCTGGC
>Pedetes capensis
CCCTTCCTCTGCAG-GT-----GGAGTCCTCTGCCCCCACTGGCTGGCTGAGT-------------------------TG
CACACTTTCCTGCTCTTATTCCCACTTCCTATCTAGGCAGGATCGTAGCAGAGAGAGGACTTTCTGGGG-------TG--
-AGAGGGATCAAGTTGAAAG----GGGATGGTGATGTCCA-GGATAGAGGCCACTCCAGTCAAGTTGCTTCTGCCTTGC-
TTCCAGGGCAGGGGG-CAGGGGAAGCT---GAGC----------------------------------------------
-----------------------------------------------------------AGCCTGCTCTTTCTGAGCAT-
--------------------------------------------------------------------------------
--------------------------------------------------------------------------------
---------------------------AGGAATGTCGTTCTGTGGATAACTTGTATGTAG-CCCACATA-CATCCCCATA
GAGCTAGTTCAGAGGGCTTT-CTGGAACCTGAGAGTTGTGTTCATCT----CCTGTCTGCCATCACAGGCTCCCTTAAAC
TCTCCC-CTAAGTCCC----TCTCTGCTCCCCCA-----AG-GCTCTGGCGGCTCTTTCAATCTCTCAGGATCCAGCGAC
ATGTTTCTGGGGATGCCCGG-
>Anomalurus sp.
CCCTTCCTCTGCAG-GT-----GGAGCCCGCTGCCACC-CTTGCTGGCTGTTT-------------------------TG
CACAC-TTCCTGCTTTTGTTCCCACTTCCTATCTAGGCAGGATCATAACAGAGAGAGGACTTTTTGGGG-------TG--
-AGAGGGACCAAGCTGAGAT----GGGGTGGTGATGTCAG-GGACAGAGGCTGTACTGGTCAGGTTGCTTCTGCCTTGC-
TTCCAG--CAGGTGGGCAGGGGAGGCTCTGGAAC----------------------------------------------
-----------------------------------------------------------AGCCTGCTCTTCCTGAGTGT-
--------------------------------------------------------------------------------
--------------------------------------------------------------------------------
---------------------------GGGAATGTTGTTTTGTGGATAACTTGTGTGTGG-CCCACATA-CAGCCCCATA
GAGCTAGTTTAGAGGGCTTT-CTGGAACCTGAGAGTTGTGTTCATCT----CCCATCTGCCATCACAGGCTCCCTTGTAC
TCTTCC-CTAACTCCC----TCTCTGCTCCCCCA-----AG-GCTCTGGCGGCTCTTTCAATCTCTCAGGATCTGGAGAC
ATGTTTCTGGGGATGCCCGG-
>Castor fiber
CCCTTCCTCTGCAG-GT-----GGAGCCCACTGCCACC-CTGGCTAGCTGTTC-------------------------TG
TATAC-TTCCTGTTTTTGTTCCCGCTTCCTGTCTAGGCAGGATTATAGCAAA-AGGGGACTTTTGAGGG-------TT--
-AAAGGGACCAAGCTGAGATAGGATGGATGACAATGTCAG-GGACAGAGGTCACTCCAGTAAAGTTACTTCTGCCTTTC-
TCCCAG--TGAATGGCCAGGGGAGGCTCTGGAAA----------------------------------------------
-----------------------------------------------------------AACCTGCT----CTGATTGTG
GGAATGT--TGTTTTGGGCT-TGGTTTGGTTTGGTTTTTTGATACAGAAT------------------------------
-CTTACTATGTAGTCCAGGCTC-GTCTGGAACTCC---TGATTTC-------CCTGCCTCAGCCTCCCCCGGTGTGTGAT
TACAGATGTGTACCACCATGTCTGGCTGGGAATGTTGTTTTGTGGGTAATTTGTGTGTAG-CCCACTTAATATCCCCACA
GAGCTAGCCTAGAGAGCTTT-CTAGAACCTGAGAATTGTGTACATCT-----------GCCATCACAAGCTCCCTTGTAC
TCTTCC-TTAATTTGT----TCTC-GTTTCCTCA-----AG-GCTCTGGTGGCTCTTTCAGTCTCTCAGGATCTGGAGAC
ATGTTTCTGGGGATGCCCGGC
>Dipodomys ordii
CCCTTCTTCTGCAG-GT-----GGAGCTCACTGCCACA-CTGGCTAGCTGTTA-------------------------TA
CACAG-TTCCTGCTTTTGTTCCCATTTCCTGTCTAGGCAGGATCATAGCAGA--GAGGACTTTTGGGGG-------TT--
-AGGAGGTCCATGCTAAGAT-----GGGTGATCGTGTCAG-GGTCAGAGGTCACTCTAGTGAAATTCCTTTTGCCTTCC-
TCCCAG--CGGGTATCTA---GAAGTTCTAGAAC----------------------------------------------
-----------------------------------------------------------AGCTTGCTTTTTCTGAGTGTG
GGTATGTC-TCCACTTGATT-TGGTTTGGTTTGGTTTTATAAGATGGGGTCTCACTATGTG-GTCCAGGTTGACCTAAGA
ACTCACTATGCAGTCGAGGCTG-GTTTTGAACTCG----GATCTT-------CCTATCTCAGCTTC-TCAGTACTGGGAT
TTCCGGCATATACCACCATGCCCAGCTGGGAAGGTTGTTTCATTGGTGATTTGTGTATAA-TCCACATG-CATCCTTGCA
GAACTAGTTCACAGGGTTTT-CTGCAACATGAGGATTGTGTCTATCT-----------GCCAACTCAGGCTCCCTGGTAC
```

```
TCTCTC-CTAATTCCT----TCTCTGTTTCTCCA-----AG-GCTCTGGCGGCTCTTTCAATCTCTCAGGATCCGGAGAC
ATGTTTCTGGGGATGCCCGGG
>Heteromys gaumeri
CCCTTCTTCTGCAG-GT-----GGAGCTCACTGCCACC-CTGGCTAGCTGTTC-------------------------TG
CACAT-TTCCTGCTTTTGTTCCCATTTCCTGTCTAGGCAGGATCATACCAGAGAGAGCACTTTTGGGGG-------TT--
-AGAAGGCCTGAGCTAAGAT-----AGGTGATCCTGTCAG-GGTCAGAGGTCACTCTAGTGAAATTTCT-----------
--------------------------GGGAGAAC----------------------------------------------
----------------------------------------------------------CAGCT--CTTT--CTGAGTGTG
GGTATGTCTTTGGTTGAATT-GGGTTTGGTTTGGTTTTATAAGACGGGGTCTCACTATGTG-GTCCAGGTTGACCTAAGA
ACTCACTATGCAGTCGAGGCTG-GTTTTGAACTCG----GATTTTTTTTTTTTTCTACCTCAGCCTC-G-CATACTGGGAT
TACAGGCTTTAATCACCATGCCTGG--TGGAAGGTTGTTTTGTTGGTAATTTGTGTATAA-TCCACATA-----------
---CTAGTTGAGAGGGTTTT-CTGCAACATGAGGGTTGTACACATCT----CCCTTTTGCCATCTCAGGTTCCCTGGTAC
TCTCCC-CTAATCCTT----TCTCTGTTTCCCCA-----AG-GCTCTGGCGGCTCTTTCA--------------------
---------------------
>Chaetodipus baileyi
CCCTTCTTCTGCAG-GT-----AGAGCTCACTGCCACC-CTGGCTAGCTACTC-------------------------TG
CACAC-TTCCTGGTTTTGTTCCYATTTCCTGTCTAGGCAGGATCATGGCAGAGAGAGGACTTTTGGGGG-------TT--
-AGAAGGTCCAAGCTAAGAT-----AGATGATCATATCAG-GGTCAAAAGTCACTCTAGTAAAATGTCTTTTGCCTTTC-
TCCCAG--TTGGTACCTA---GAAATTCTGGAGC----------------------------------------------
-----------------------------------------------------------AGCTTGCTCTTTCTGGGTGTG
GGGATGTC-----TTTGATTTGGGTTTGGTTTGGTTTTATGAGACAAGGTCTCACTATGTGGGTCTAGGTTTACCAAAGA
ACTCACTATGCAGTCTAGGCTGTGTCTTGAACTCT----GATCTT-------TCTACCTCAGCCTC-CCAGTACTAGGAT
TACAGGCCTTAACCACCATGCCCACCTGGGAAGGTT----TTTTGGTAATTTGTGTGCAA-TCCACATC-----CCTGTA
GAACTCGTTCACAGGGTTTT-CTRCAACATGAGGCTTGTATACATCT----CCTATCTGCC----------------TAC
TCTCCC-CTAATTCTT----TCTCTGTTTCTCCA-----AG-GCTCTGGCGGCTCTTTCAATCTCTCAGGATCCGGAGAC
ATGTTTCTGGGGATGCCCGGG
>Cavia porcellus
CCCTTCCTCTGCAG-GT-----GGAGCCCACTGCCGCC-CTGGCTGGCTGTTT-------------------------CA
CGCAC-TTCCTGCTTTTGTTCCCACTTCCTGTCGAGGCAGGATCATAGCAGAGGGAGGACTTTT-GGGA-------TG--
-AGGAGGACCAAGCTGAG-C---GGGGTTGGTGATGTCAGGGGGCAGAGGTTACCCCAGCAAAGCTGCAGCTGCCCTTC-
CTCCAG--TGGGTGGCAAGAGGCGGCCCCAAAAC----------------------------------------------
-----------------------------------------------------------AGCCTGCT-TGGCTGACTGTT
--------------------------------------------------------------------------------
--------------------------------------------------------------------------------
----------------------------GGAATGCT---TTGTGGTTGATTTGTGGGTAGCGGCAGCTT-GGCCCCCACA
-AACTAGTTCTGAGGGCTTT-CTGGAAGCCGGGAGTTGTGTCCACCT----CCTGTTTGCCATGGCTGCCTCCCTTAAA-
-CTTCC-CTGGTTCCT----TCTCCCCTCCCCCA-----AG-GCTCTGGCGGCTCTTTCAATCTCTCAGGATCCGGAGAC
ATGTTTCTGGGGATGCCCGGG
>Ictidomys tridecemlineatus
CCCTTCCTCTGCAG-GT-----GGAGCTCACTGCCACC-CTGGCTA----TTT-------------------------TA
CATAC-TTCTTGCTTTTGTTCCCACTTCCTATCTAGGCATGATCCTATCAGA-----GAATTTTGGGGGT------TG--
-AGAGGGACCAAGCTGAAAT----GGGGTGGTAATGTCAGTGGACATAGGCCATTCCAATGAAGTTGCTTCAGCCTTTC-
TTCCAG--TGGGTGGCCAGGGAAGGCTTTGGAAT----------------------------------------------
-----------------------------------------------------------CTA-------TTCTGAGTGCT
--------------------------------------------------------------------------------
--------------------------------------------------------------------------------
----------------------------GAAATGTTGTTTTGTGGATAATTTGTATACAGATATACATA-TGTCCCTACA
GAGCTAATTTAGAGGGCTTT-CTGGAACCTGAGAGTTATGTTCATCT-----------GCCACCACAGACTCCATTATAC
TCTTCT-CTAATTCCC----TCTTTACTCCTCCA-----AG-GCTCTGGCGGTTCTTTCAATCTCTCAGGATCCGGAGAC
ATGTTTCTGGGGATGCCTGGG
```

Marker 64100

```
>PB1D10
-------------------------------------------------------------------------------A
GCCGGGCGTGGTGGCGCACGCCTGTAATCCCAGCACTCGGGAGGCTGAGGCAGGAGGATCGTGAGTTCAAGGCCAGCCTG
GGCTACATAGCAAGACCCTGTCTCAAAAAAACAAAA--------------------------------------------
--------------------------------------------------------------------------------
---------------------------------------------
>Mus musculus
AAA-------CCGTCCCTG-----ACATCACTGGGT------------TTTAGC-ATAGTTTTTGGACGAGCTGCCCCTT
G-------------------------------------------------------------------------------
-----------------------------------------------------------------GTGATCTTTATAGCA
TAGTTAAGGAAAAATGGGGTTGAGACC-----------------TACGAGGGAGGCTAGGAGATAGACGC----------
GAGTACCAACCAGACTGACTGACATTCCACCTC-----AG-GAAC
>Rattus norvegicus
AAA-------TTGCCCCTG-----ACATCACTGGGT-------------TTTGTCATGGTTTTTGGAAGAACTGCCCCTT
G-------------------------------------------------------------------------------
-----------------------------------------------------------------GTGATCTTTATAACT
TAGTTAAGGAACAATG-AGGTGAGACC-----------------TGAGAGGGAGACTAGGAGATAGACTC----------
GAGCCCCAACCAGA----CTTTCATTCTGCTTC-----AG-GAGC
>Apodemus sylvaticus
-------------------------------TGGAT-------------TTTATCATGGTTTTTGGAAGAGCTGCCCCTC
G-------------------------------------------------------------------------------
-----------------------------------------------------------------GTGATTTTTATAGCT
TAGTTAAGGGAAAATGGGGTTGAGAAC-----------------TGAGAGGGAGACTAGGAAGTAGACTG----------
GACTACCAACCAGACTGACTTCCATCCCACCTC-----AG-GAGC
>Peromyscus maniculatus
AAGAAA----CCCTCCCTG-----ACCTCACTGGGT--------------TTGTTGTGGTTT--GGAAGAGCTGCCCCTT
G-------------------------------------------------------------------------------
-----------------------------------------------------------------GTAATCCTTGCAGCT
TAGTTAGGG---------------ACC-----------------CGAGAGGGAGACTGGGAGATGGGCTCACAGCATGGA
GGGTACCAACGAGAATGCCCTTCATT-CCTCTC-----AG-GAGC
>Microtus agrestis
AAAAAAA---ACCTTCCTG-----ACCTCACTGGGT--------------TTGTTGTGGTTTT-GGAAGAGCTGCCCCTA
G-------------------------------------------------------------------------------
-----------------------------------------------------------------GTAATCTTTGCGGCT
TAGTTAGGGAACAAGCGGGATGAGACC-----------------CAAGAGGGAGACTGGGAGGTGGGCTCACAGCACAGA
GGGTACCAATGAGAACGACCTTCATCCCGCCTC-----AG-GAGC
>Nannospalax galili
AAACTGAAGAAAC--CCTG-----ACATTGCTGGGTTGCACTGTAAAGTTTCCCAGTGATTTT-AGAAGAGCTGCCCTTT
G-------------------------------------------------------------------------------
-----------------------------------------------------------------GTAATCTCCATAGCT
TAATTAGGAAATAATCTAGATGGGACCTGTTGGGCTTTTGAAACCAGGAGGAGGACTGGGAAACTGGATCACAGCATGGA
AGGTACCAACCAGACCCACCTGTGTCCCACCTT-----AG-GAGC
>Jaculus jaculus
AAAAA----TCTCTTCCTT-----ACATGGCT-----ACATTATGAAATTTCCCAGTGGTTTT-AGAAGAGCTAT-CCTT
G-------------------------------------------------------------------------------
-----------------------------------------------------------------GTCATCTCTGCAGCT
TAAATAGGG-ATAATCTGGATGGGACCAATTGGGCTTT-GAAAATGAGAGGGAGACTGGGAGTCTGGGTCACAG-AT---
-----CCTGCCAGAAAGACTTGCATCCTGCCTC-----AG-GAGC
>Anomalurus sp.
------------------------------------------------------------------------TGTTCCTT
G-------------------------------------------------------------------------------
-----------------------------------------------------------------GTAATCTGTGTAGCT
TAATTAGGGGACAATCTGGATGGAATCTGTAGGTCTTT-AAAAAGGTGAGGGAGACTGGGAGACCTGGTCACAGTGTGAA
AGGTACCAATCAGACAGAACTTCATGCCACCTC-----AG-GAGC
>Castor fiber
AAAAAA--GT-ACTTCCTG-----ACATTGCTGAGTTGTGTTGTGAAATTTCCTGATGGTATT-AGAAGAGCTGTTCCTC
ACTGGGTATGCTGGCACATGCCTGTAATGCCAACACTCAGGAGGCAGAGGCAGTAGGGTCATGAGTTTCAGGCCAGCCTG
CGCTACATATTGAGACCC-ATCTCAAAAAAAACCCAAAAACAAATTAGAAAAGAGCTGTTCCTGGGTAATCTTTGCAGTT
TAATTAGGAAATAATCTGGGTGGGACCTGTTGGGCTAT-GAACATGTGAAGGAGACTGGGAGACCAGTTCCTAGCTTGTA
GGTTATCAGCCAG-CAGACCTACGTCTCTCCTT-----AG-GAGC
>Dipodomys ordii
AAAAAAAAATCACTTCCTG-----ACATTGCTGGGT-GCATTGTGACATTTCCCGGTGGTTTT-GGAAGAGCTGTTGCTT
GCTATGGGTGGTGGCACTTGCTAGTCATCCTAGAATTTAGGA-GCA------------CCATGAGTTCCA-TCCA-----
-----------GAGACCCTG-CTGACACAAAAGCCAAAACCA-----GAA--GAGTTGTTCCT-----------------
------AGGGATA-TCTGGTTGGGACCTGTTGGGCTTT-AAAAATACAAAGTAGATTAAGAGGCCCGGTACTCGGGTGGA
GGATACTAACCAGATAGGCCTCCATCTTACCGT-----AG-GAGC
>Cavia porcellus
AAAA------CAGTTCCTG-----ATACTGCTGGGTTGGATTGTGAGATTATCCTGTGGTTTC-AGAAGACCTGGCCCTC
G-------------------------------------------------------------------------------
-----------------------------------------------------------------GTGATATTGGCAGCT
TCATTAAGGGATAATGTAGATAGGACCTTTGGGGCTTT-AAAAATGTGAGGGAGACCCGGGGCCTGGATCACAGCAGGGC
GGGCAGCAGCCAGACAGCCCTGCATTCTTCCCC-----AG-GAGC
>Ictidomys tridecemlineatus
AAAAACAA-TAA-TTCCTGGGTTTATTTTGCTGGATTGCATTGTGAGGGTTCCTGGTGGTTTT-AGGAGAGCTATCACTT
G-------------------------------------------------------------------------------
-----------------------------------------------------------------GTAATCTTGGCAGTT
```

TAATTAAGGGATAATAT-----GCACCTGTTGACCTTC-AAAAGTGAGAGGGAGCCTGGGAGAAC-AGTCGCAGTATAGA
GAGTACCAACCAAACAGACCTATCTCTCGTCTC-----AG-GAAC

Marker 97783

```
>PB1D10
--------------------------------------------------------------------------------
--------------------------------------------------------------------------------
----------------------------------------------------------------------------TTTT--
------GTTTTTTTGAGACAGGGTCTTGCTATGTAGCCC-AGGCTG-GCCTTGAA-------------------------
-CTCAC---GATCCTCCTGCCTCAGCCTCCCGA-GTGCTGGGATTACAGGCGTGCGCC-ACCA-CGCCCGGCT-------
--------------------------------------------------------------------------------
--------------------------------------------------------------------------------
--------------------------------------------------------------------------------
--------------------------------------------------------------------------------
--------------------------------------------------------------------------------
--------------------------------------------------------------------------------
--------------------------------------------------------------------------------
--------------------------------------------------------------------------------
--------------------------------------------------------------------------------
--------------------------------------------------------------------------------
--------------------------------------------------------------------------------
--------------------------------------------------------------------------------
--------------------------------------------------------------------------------
--------------------------------------------------------------------------------
--------------------------------------------------------------------------------
--------------------------------------------------------------------------------
--------------------------------------------------------------------------------
--------------------------------------------------------------------------------
--------------------------------------------------------------------------------
--------------------------------------------------------------------------------
--------------------------------------------------------------------------------
--------------------------------------------------------------------------------
--------------------------------------------------------------------------------
--------------------------------------------------------------------------------
--------------------------------------------------------------------------------
--------------------------------------------------------------------------------
--------------------------------------------------------------------------------
--------------------------------------------------------------------------------
--------------------------------------------------------------------------------
--------------------------------------------------------------------------------
--------------------------------------------------------------------------------
--------------------------------------------------------------------------------
--------------------------------------------------------------------------------
--------------------------------------------------------------------------------
--------------------------------------------------------------------------------
-------
>PB1D7
--------------------------------------------------------------------------------
--------------------------------------------------------------------------------
----------------------------------------------------------------------------TTTT--
------GTTTTTTTGAGACAGGGTCTCGCTCTGTAGCCC-AGGCTG-GCCTCGAA-------------------------
-CTCACGGCGATCCTCCTGCCTCAGCCTCCCGA-GTGCTGGGATTACAGGTGTGAACC-ACCA-CGCCCGGCT-------
--------------------------------------------------------------------------------
--------------------------------------------------------------------------------
--------------------------------------------------------------------------------
--------------------------------------------------------------------------------
--------------------------------------------------------------------------------
--------------------------------------------------------------------------------
--------------------------------------------------------------------------------
--------------------------------------------------------------------------------
--------------------------------------------------------------------------------
--------------------------------------------------------------------------------
--------------------------------------------------------------------------------
--------------------------------------------------------------------------------
--------------------------------------------------------------------------------
--------------------------------------------------------------------------------
--------------------------------------------------------------------------------
--------------------------------------------------------------------------------
--------------------------------------------------------------------------------
--------------------------------------------------------------------------------
--------------------------------------------------------------------------------
--------------------------------------------------------------------------------
--------------------------------------------------------------------------------
--------------------------------------------------------------------------------
--------------------------------------------------------------------------------
--------------------------------------------------------------------------------
--------------------------------------------------------------------------------
--------------------------------------------------------------------------------
--------------------------------------------------------------------------------
```


>ID4

--TTTTTT-------TGCGGTGCTGGGG
ATTGAACCCAGGGCCTCGCG-CATGCTAGGCANGCACTCTACC-AC-TGAGCTACATCCCCAGCCCC-------------
----

>Mus musculus
AAAA-ATACATAGACACATCAAGAGTGAATATAAAAAATAAAATTGTCTTCCCAATTTTGTCCTGT------CTGATT--
CTTTGATTCCTCCCTTTCCATTCTCTGTCCCTCCT--TTGT---------------------------------------
----

---TGTCGTG
GTTTTAATGAGAATGACCTCCAAAGGCTCATATATTTGAATTTTTAGTCACCAGGGAATGGCATTATTTGAAAGGATTAA
GAGATGTGGCCCTGTTGGAAGAAGTGTGTCACTGGGGGTGGACCAAGCCTAGAACCATTTTCTTCATGATGCTTGCTGAT
CCTGATATAGAGTTCTCAGCTACTTCTCCAACACCATGTTTGCCTACCACTAT---------GTTTTCTGCCATGATGAC
AATGGACCTCTGAATCTGTAAGCAAGCCCAGTTAAACATGTTCCTCTATAAGAGTTACCATGGTCATGGTGTCCCTTCAC
AGTAAACAGAACAATGAGAGGAGTTGGTACCAGGAAGCTGGGTATTGCTGTGACAGGCCTGACCACTCTGCTTGTTGGTG
GAATGTGGACTTTGTGACTTTGTATTAGGAAAGAAGTTGGATGCTTTAAGCAGGTCTTAGTGGGCCATGCTAGCAGGAAC
ATGGAAGGCAGTGGTGCTGAGAGCAATGGAGATTATGATGGCCCAGCTCAATATGTTTCAGAGGGGAAGAATACTAGTAA
GTGGCCTAGAAACTGTTCTTGTGGTGTTTTGGTAAAGAATGTGGCTGTTTTCTGCCCCTGTCCTAACAATATGCCTGAGG
CTAAATTGAAGACTTTTGGATTAATGGTGTTGGCAGAGGAGATTTCAAGACAAACTAATATTGACTGTGTTACATGGTTA
TAAGTGGTAACTCTTATGCAGATCTATAATTTAAAGGAGCAAGTTAGCGAGGAAAAAAATATAAAATGTACAGTTT----
----AAGGAGCATTGGAAAGTGCAATGAAGCCACGTCCTGTAC----------TAAAGGAGGTGAAAAAAATCTGATGCT
AAGTGTAATAAAGGGAATGGTGACCTCATAGCAAGAAAGCACCCAGCGGAACTTCCAACTTATGAAAAGTAATTAAAGAA
AAAAAAGTTTAGCAGTGAAAAAAACCATAGAAAACAGAAAGATGGTGTAGATGTAACAGAACAAGTGGGGCATGTTTCAG
CCCCAGCAAACAGAAGAATTTGACAGCTTCAGCCACATGGTTCTGGCTTTAGAGTTAAGGATAGAACAAAGGGGTTATGG
AAGCTCCCTCTGTGATGAAGGAAAGGCTAGGTATGTGTTCTTCCGTGGAGGCCTAGAGAGGCCACTGTGTGAAACTGTGA
AAGTAAAACCTGGATTGCCTTGGAGACCTCAAGATGTTGTAGATGCTAGAGTCATCTGAGA--TGCTGAGAAAACCTGCT
ACAAAGGAGTAGAACCATTACAAGAGAAAGAA--GGGTTGAAGTCAACAAAGGTCAACATACAGTGGCACAGAACACACG
TTACCATTCCAAAAGGGAGGAAAGGGAGCGTAATGAGGAAATAGTGGAGCAAAGCAAGATTGAAAGCTGGGCACACTCCA
AACTCTGCATCTGTGTCTGATGT---

------------------------CCACAGGCTCATATATTTGAATGCTTAGTCATCAGGAAGTGACACTACTTGAAAGG

ATTAGGAGGTTTGGCCATGTTGGAAAAAGTGTATCACTGAGAGTGGGCTTTGAGGCTTTCAAAGCCCATATCAAGCCCAG
AGTCTCTCTTCCTCCTGCCTGCAGATCTGAATGTAAAGCTCTCACCTACTTCTTCAGCATCATCTCTGCCTGCTCGCCAC
CATGTTTCCCACCATGATAACAATGGACTAAACATCTGAAAGTTTAAGCCCGACACAACTAAATGCTTTTCTTTGTAAGA
GTTGCCATGGTCATGGTGTCTGTTCACCATGTAATAGAACATTGATTAAGACATTTACTCTTTTCATTTTTTTTTAAAGA
TTCA----------CTTTTGTCTACATGAGTGTGGGTTCACCA----CATG-AATACCTAGTACCTACATA-GA-TCAGA
AGAATGCATTAGATACCCTAGAACTGGATGGGTGTCATCTACCATATCCATGCTAGGAACCAAACTTGGGTCTTCTGCAA
GAACAAGAAATATTCTTAACCACTGAGTCGTCTCTCTAGTGCC----GTGTGTGTGTGTGTGTGTGTGTGTGTGTGTGTA
TGTGTGTGTGTGTGTGTGTAGATTAGAGGCCACCTTTTGGGAATTGTTTCTATCCTAACACC-TGT--GTTCCAGGAACT
GAACTTGGGTTGTAAAGCTTGACAGAAAACGCCTTTACCTGCTTGAGCATCTTGCTAGCTTGCCTTTTCAAAAGGGAGAT
AGTTGTTTGCATTCAAATGTTTGTGCATTTGTACAAATATATG-------TGTGGCGGGCCTCAGGGACCCGAAGAGGGT
GTTGGATGCCCTAGAAAGGCAATTACAGGTAGTTGTGAGCCACCCAGCATGAGTACTAGGAACAAAACA-GA-TCCTCTG
GAAGATCAGCAA--GTGCTTTTAACAGCTGAACTGTTTCTCCAGCTCCATCCTTCTCTGCA-TTTTTAATGA--CTCTGT
AAAATCCCTTTAGATTCA--GTGTGT---------------CTCCCTCTACTATACAGGCACTCTGACACTTAGAACAGT
TTGGGTT
>Rattus norvegicus
GAAA-ATACATAGACATGTCAAGAGTGGATATAAAGAATAAAATTGTCTTCTCGATTTTCTCCTCT--------------
-------------CTTTCCATTCT--------CCT--TTAC---------------------------------------
----

---TGTAGTG
GTTTGAATGAGAATAGCCTCCATAGACTCTTATATTTGAATTTTTAGTCACCAGGAAATGACACTATTTGAAAGGAGTAA
GAGATGTGGCCCTGTTGGAAGAAGTGTATCAAT-AGGATGGGCCAAGTCCAGAGTCACTTTCTTCATGCCTGATGATCCA
GATATAGAGCT---CTCAGCTACTTCTCCAACACCATGTCTGCCGATACGCCACCATACTGACTGCCATGATGACAATGG
ACTAAACCTCTGAATCTGTAAGCAAGCCCAGTTAATCGTGCTCTTCTATAAGAGATACCACGGTTGTGGTGTCTCTTCAC
AGTAAACAGAACAGTGAGAGGAGTTGGTATGAGGGAGCTGGGTATTGCTATGGCAGACCTGACCATTCTGCTTGTTGGAG
GAATGTGGACTTTGAGACTTTGTATTAGGAAACA-GTTGAATACTTTTAGTGGGGCTTAATGGGCCATGCTAGCAGGGAC
ATGGAAGGCTGTGGTACCGAGAGCAATGTAGATTATGATGGCCCAGCTAAAGAGGTTTCAGAGGAGAAGGATATTAGTAA
GTGGCCTAGAAACTGTTCTTGTGGTGTTTTGGTGAAGAATGGGGTTGTTTTCTACCCTTGTCCTAAAAATCTGCCTGAGG
ATAAATTGAAGACTTTTGGATTAATGGTGTTGGCAGAGGAGATTTCAAAACAGCCTATGTCACATGGTTATTACTGGCCA
GTTTTATACAGATCTATA----------ACGAAAAGGAGCAAGTTCTGCAAGAAACAA-TATAAAATGTACAGTTTGAGG
GGGAAAGGAGTATTGGAAAGTACAATGGAGCCAAGTCCTGTGCTCAAGGAGTTAAAAAGCACTAAAGAAAATCTGATTCT
AAATGTAATAAAGG-AATGGTGACCTCATAACAAGAACCCACCTAGTGGAACTTCCAATTTTTGAAAAGGAATTAAGGAA
AA----GTTTAGCAGTGGAGGAAACCATAGAAAACAGAAAGATGGTATAAATGTAATAGAACAAGTAGGGCATGTTTCAG
CCCCAGCAAACAGAAGAACTTGACATCTTCAGCCACATGGTTCTGGCTTTAGAGTTAAGGATAGAAGAAAGGGGCTATGG
AATCTCCCTCTGTGAGGAAGGAAAGGATAGCCGTGTGCTCTTCCATGGAGGCCCAGAGAGGCCCGTATGTGAAGCTGT-A
AAGGAAAACCTAGCTTGCCTTGGAGACCTCAAGATGTTGGAGATGCTAGACTCATAGGATACCTGCTGAGAAAACCTGCT
ACAAAGGAATAGAACCATTACAGGAGAAAGAAATGTGTTGCAGCCAACAAAGCTGAAAAGAGTTGGACATCACACACGGA
GATGAGGAGTTTGGAGTGTGCCCAGCTTTCAGTCTTGCTTTGGTCCACTATTTCCTCATTATGATCCCCTTCCTTCCTTT
TGGAGTGGTAACATGCATTCTGTGCCATTGTATGTTGAAAGTATGTGATCTGTTTTTTCATTTTGATTTTGTAGGGGCTT
AAATTTAAAAATTGCTTTGAGTCTCAGAAGAGACTTTGAACTTTGGACTTTTAAACAGTGTTGAGACTGTTACAGACTAT
GAGGGGTTTTGACATTGGACTAAAAATACTTTTTCATTATGATATGGCTATAAGCCTATGGGGGACAGGGAGTGGAATGT
GGTTGTTTGAATAAGCATTAACTCCCACAGGCTCATATATTTGAATGCTTAGTCACTGGGGAGTGACGGTATTTGAAAGG
ATTAGGAGGTATCACCCTGTTGAAGAAAGTGTATCA--
---CTCTTTTCCTCCTGCCTGCAGATCTGGATGTAAAGATCTTAACTACTTC---AGCATAATTTCTGCCTGTCCGCCAC
GATGCTTT---CCACCATAACAATGGACTAAACATCTGAAACTGTAAGCCAGCCCCAATTATAAGAGTTGCCATGAGGGT
TGGGGATTTAGCTCAGTGGTAGGGCGCTTGCCTAGCAAGTGCAAGGCCCTGGGTTCGGTCCCCAGTTCTGAAAAAAAAAA
GAAAAAGAAAAAAAAAAGAGTTGCCATGCATGGTGTCTCTTCAC---AGTAATAGAACTCTGACCAAGACATAGGTTAGA
AGAACACATTGGATGCCCTGCAACTGGATGGGTGTGATCGACTTTATCCATGTTGGGAACCAAACCTGGGTCTTCTTAAG
AAAAAA--

>Nannospalax galili
GAAT-ATGTACTGCTAAGCCTCCAGTGGATGGAGTGGGTGAAATTGTCCTA-CAATTTTGTCCTTC------TTCATCCC
CTTTGACTCGTTCCT-CCCAGATCCC-TCCCTCCT--TTACT--------------------------------------
----

---CTTCTCAGGAAAAATTTTAAAGTATTTTTTTATGAGT
ATGAGT--------GTTTTGCCTGCATGTATGTAAGTGCCCTACGTGTGTGCAATACCCGAGGAGGTCAAAAGAGGGAGT
CAGATCCCCTAGAACTGGAGTTA-AGGACGGTTGTCAGTTGCCATGAGGGTGCTGGGAACCAAACCCAGGTACTATGCAA
GAGCAGCCAGTGCTCTTAATCACTGAGCCATCTCTCCAGCCCCACTTTTTTTTCAATTAATTTTTTTTAAAAAAATTA--
---------GTGTGTGTGGAGGTCAGAGTACAACTTGCAAGATTCAATTCTCTCCTTCCGGGATGTGGGTTCTGGGGATT
GAACTCAGGTTATCAGGTTTAACAGCAAGTGCCTTTACCTGCTGAGCTCTCTCACCAGTCCCTCTTTTTAAAAAATATTT
C--TTTTTTTATGTGTATGTGTGTGCATTTATGTGAATGTATGCCACCTATGTGTATGTGCCTGTGGAGGCC-AGAAGGC
TTTGGATCCTCTGGATCTGGAGTTTCAGGCAGTTGTGAGCCACC-AGATGAGGTACTGGGAACTGAACTTGAGTCTTTTG
GAAGAGCAGCAAGTGTGCTCTTAACCTCTGAGTCATCTCTCCAGCCCCATCCCTCTTTTCACTTTTTAATGAATCTGTGT
AAAATCCCTTCAGATTCATAGTGTGT---------------TTCTCCCAGCTCCACAGGCACTTTGACCCTTAGAACAGA
CCGGGGT
>Jaculus jaculus
GAAT-AAGCATTACTATGTCTACAGTGA-TGGTGAGGGTCAAATTGTCCTCCCATTTTTTTCCTGC------CTTATTCT
CTTTGATTCCTTCCT-CCCATTCTCTCTCCTTCCT--TTT--

---TACTTTTTTTCACTTTTTAATCAATCTGTCT
AAAATCC-TTCCGACGTATAGTGTGC---------------TTCTCCCCACTCCACAGGCACTCTGACACTTCGAATCGA
CTGGGCT
>Anomalurus sp.
GAAT-AAGCATTGCTAAGCCTGGAATGAATGGAGAGGGACAAATTGTCCTCCCAATCTCATCCTGC------CCCCTTCC
CTTTGACTTCTTCCT-CCCATCTTCTCTTACTCCCTCCCTCCT-------------------------------------
----

---TTCCTCTTTTCATTTTTTAATTAATCTGAGT
GAAATCACTTCGGATTCATAGTGAGC---------------TTCTCCTCACTCCACAGGCACTCTGACGCTCAGAATGCA
TCAAGTT
>Castor fiber
GAAT-AAGCATTGCTAAGCTTGTAGGGAACAAAGAAGGTCAAATTGTCCTCCCAATGTCATCCTGCCCCCATCTCATCTC
CTTTGATTCCTTCCT-CGCATCCTCTTTCTTTCCCTCTTATTTTGTTTTCTCTTTCTTTT-------TGCAACGCTAGGG
ATCAAACCTAGGGTCTTGTG-CATGCCAGGCAAGCGCTCTATA-AT-TGAGCTACATCCCCAG----CCTTT--------
------GGTGTTTTGA--GCCAGTCTCGATATGTAGCAC-AGGTTG-GCCCTGAA-------------------------
-CTT---GCAATCCTCTTGCC------TCCC-A-GTCGTGGGTC---------------ACCA-CACCCGAA--------
----

---GTCCTCTTTTCATTTGTTAATAAATCTGAGT
AAAATCTTTTCAGATTCAGA-TGAGTTT--------------TTCTCCCCACTCCACAGGCGC--TGACACTTAGAATGGA
CCGGGTT
>Dipodomys ordii
GCAT-AACCATTGCTAAGATTGTAGGGAATGGAGAAAAAACTATTTTCCTCTCAATTTCATCCTGC------CCTATCCC
CTT-GGTTCTTTGCT-CTTACTCTCTTCTGCTGAACCCCCTCCCCT---CTCTTTTTGTGGTACTGGTGCAG-AGGAGGG
GTCAAATCAAAGACCTTGCCTTATGCCAGGCAAGCAGTATATATATCTGAGGTACATTCCCAGTCCCACTTTTTTTTTGG
GGGGGGTGCTTTGTATTTGCCAGGCA-GCCAC-TAGCACTGAGACATGCCTCCAATTCCTT-CACTTTTTTTTTTCCCCC
AATACAGCCTCCTTATGTAGCTCAGCCTGCCTACGTGCTGGGATTACAGGTGTTAACCTACCAAGGCCCAGC--------
----

---ATCTTCTTTTCATTTTTTAGTAAATTTAAGA
AAACTCCCATCCGATTCATAGTGAGCATCTTCCCGCTCCACTTCTTCCCGCTCCACAGGCGCTCTCACACTCAGAA--GA
CCTGGCT
>Cavia porcellus
GAAT-AAACATTGCCAAGACTAGAGTGAATGGAGAAGGCCAAATTGTCCTTACAAGTTCATCCAGC--------CATCCC
CTTTGACTCCTTCGA-TCTTTCCTTT-CTCCTTCC--CTCT---------------------------------------
----

--TTTCA-TTTTTAATGAATCTGTGT
AAAATCTTTTCAGATTCATAGTGAAC---------------TTCT-CTCATGCCACAGGCTCTGTGACATTCAGAATATA
CCAGGCT
>Ictidomys tridecemlineatus
GAATTAAGTATTGGGAAGCCTGAAGTGAATAGAGAGGGTCAATTTTTTCTCCCAATTTTGCCCTGC------CTCATTCC
CTTTGACTCCTCCCT-CCCCTCCTTCCATTCTTCT--TTCT---------------------------------------
----

---TTCCTCTTTTCATTTTTTAATGAATCCAAGT
AAACTACCTTTAGATTCATAGTAAGC---------------TTCTCTCTACTCCACAGGCTCTTGAACACTTAGAATGAA
CCCCACC

Marker 62793

```
>PB1D10
--------------------------------------------------------------------------------
---------TTTTGTTTTTTTGAGA-CAGGG-TCTTGCTATGT-----------AGCCCAGGCTGGCCTTGAACTCACGA
TCCTCCTGCCTCAGCCTCCCGAGTGCTGGGATTACAGGCGTGCGCC--ACCACGCCCGGCT-------------------
--------------------------------------------------------------------------------
--------------------------------------------------------------------------------
--------------------------------------------------------------------------------
--------------------------------------------------------------------------------
--------------------------------------------------------------------------------
--------------------------------------------------------------------------------
--------------------------------------------------------------------------------
--------------------------------------------------------------------------------
--------------------------------------------------------------------------------
--------------------------------------------------------------------------------
--------------------------------------------------------------------------------
--------------------------------------------------------------------------------
--------------------------------------------------------------------------------
--------------------------------------------------------------------------------
--------------------------------------------------------------------------------
------------------------------------------------------------------------------
>Mus musculus
CAGGGTGGTTTGTTTGTTTGTTTGTTTGTTTGTTTGCTGGAATTACC---CTGCTTTACCC-GAAGGGATTCTTTTT---
--------------------------------------------------------------------------------
---------------------------------------------------------------------ATCTGTTTAAT
TTACCTGCCACACACATGGGCTGGATCCATAAGGGCAATTATTTA-GCCATCTTTCATTCAAATAAAAAAA--TCTAAAT
TATTGTCATGTATGTATT-TAAGTATGTTG-------TCCCT-TTG-AATGAAA---------------------GAC--
---TTTTG-------TCAGAACACAGTCAAAAGGATAG------------------------------------------
--------------------------------------------------------------------------------
--------------------------------------------------------------------------------
---------TGCAT------------------------------------------------------------------
-----------------------------------------------------------------GCTGAACGATGTCTT
-GGGGACCTGTGAGAGAGCCAGGATAAA----GGCAGAGGGCCTTTTACCTCAGGCGCATTTTGTTCCCTGAGTTGTTTT
AAGGTGTGACTTCATGCCTCCTGTGACAAACGTTTATATTC-----AATTTAT--------------AAGGCA-TGTTTA
TTCCTATTGCATGTCAGCTCATAGCTCTAGAATCCATTCTGGTCAGTGTGCCCTGAATCTGGATATGGCCTT-CTGCCTC
GCAGTCCTGCTTTCTGAGAAACGAGCTATACAACATGAGAGACAACTGTGTTTAAATTGGTCTGTCCTGAGGAAGTTCTG
GGAA-GGGCTGCTCTGTTAATATTTAGTATGTGCTTACTGACACTTATTTACATGTTGTCCTG-----------------
--ATTGCTTTCTTTGTTACATTCGTGTATACAAGTCCACTGAAATGGAAAGAAGGCTGTCTACAGCATCAGACTCTAGTC
TGCCCCTTTCTCTCCAGTCCTCAGATCCCAGGTC-AGCACACAAGATCTGCACAGGTCAGTGAAAGTCAACAATCTCTCC
TTTCTGAATATATCTATTTTCCAT-TTTGAGAGGATCCATTTT-----AC-CTTGTGCCGAGTGAGAGCCACTTGATGGT
CCCTTAAATTTATCTCTGAAGTCCTTTGCAATTCGCCGAGAAACTGCTTAATCCAGACAGAGAAGGAAAATAGCAGTT
>Rattus norvegicus
CAGGGTGGCTTTTT---------GTTTGTTTGTTTTCTGGAGTTACC---CTGATTTACCC-AAAGTGG---TTTTC---
--------------------------------------------------------------------------------
---------------------------------------------------------------------GTCTGTTTGAT
TTACCTGCCCAACACATTGGCTGGATCCCTAAGTTCAATTATTAA-GCCATCTTTCATACAAATAAAAAAAAGCCAACAT
TATTGTCATGTATGTATT-TAAGTGTGCTG-------TTCCT-TTG-AACTAAA---------------------GAC--
---TTTTG-------ACAGAATACATTCAAAAGGACGA------------------------------------------
--------------------------------------------------------------------------------
--------------------------------------------------------------------------------
---------TGCAT------------------------------------------------------------------
-----------------------------------------------------------------GGTGAGCAATGTCTT
CAGGGGCCTGGGAGAG--CCAGAAAAAAAAAAGGCAGAGGGCCTTTTACCTCAGGTGCATTTTGTTTCCTGGGTTGTTTT
TAGAAGTGACTTTGTGCCTCCTGTGACAAACATTTATATAAGGCCCAATTTATTTATTTATATAGATAAATTTATATTTA
TTCTTGTTGCATATCAGATCATTGCTCTAGAACCCAATCTGGTCAGTGTACCCTGAATCTGGATATTGCCTTTCTGCCTT
GCTTTCTTGCTTTCTGAGAAACAATCTATGCTACACACAAGGCAATTGTGTTTAAGTTTGTCTGTCCTGAGAAAGTTCTG
AGAAAGGGTTACTCTGTTAATATTTAGTATGTGCTTACTGATCTTTATTTGCATGTT-TCCTGTCACAACCTTCCTTGGC
TCATTGC---CTTTGTTACATTCGAGTGTAAAAGTACACAGAAACTGAAGCAAGGCTGTCTACAGCATTAGACTGTAGTC
CACCCCATCTTTTCAGGTCCTTAGATCACAGGTTGAGCAAATAAGATCTACCCGGGTCAGTGAAAGGCGACAAACTCT--
TTTCTAAATGCATCTATTTGCCAT-CTTGAAAGAATCCATTTT-----AC-CTTGTGTCGGGTAAGAGCCACTTGATGGT
CCCTTAAATTTATCTCTGAAGTCATTTGCAATTCACTGAGAAA-----------------------------------
>Apodemus sylvaticus
CAGGGGTTTTGGTTTGTTTGTTTGTTTTGTTTTTGCTGTAGTTACTCATCTGCTTTCCCT-GATGTGA---TATTC---
--------------------------------------------------------------------------------
---------------------------------------------------------------------TTCTACTTAAT
ATTCATGCCCAGCACCCTGTCTGGATCTATAAGTTCCATTCTTAG-ACCAA-TTTCATAC-AATAAAAAA---TCTTAAT
TGCTTTCATGTATGTA----------------------------------------------------------------
--------------------------------------------------------------------------------
--------------------------------------------------------------------------------
--------------------------------------------------------------------------------
--------------------------------------------------------------------------------
--------------------------------------------------------------------------------
--------------------------------------------------------------------------------
--------------------------------------------------------------------------------
--------------------------------------------------------------------------------
--------------------------------------------------------------------------------
--------------------------------------------------------------------------------
--------------------------------------------------------------------------------
```

```
--------------------------------------------------------------------------------
--------------------------------------------------------------------------------
--------------------------------------------------------------------------------
------------------------------------------------------------------------------
>Peromyscus maniculatus
TA---TTTTGTTGTTGTTGTGTTGTGTTTTGTTTTGTTGGAGTTACCCAGCTGTTTTACCT-GAAGTGA---TTTTT---
--------------------------------------------------------------------------------
---------------------------------------------------------------------ATCTGCTTAAT
CTGCCTTCTCAACACAGTTGCTGGGTCCATAAGTTCAATCATTAA-GCCATTTTTCATAC-AACCAAAAG----CTAAAT
TGTTGCTGTGTATGTATTTAAGTGTGTTGT--------CCCT-TTG-AACTCAA---------------------GGACT
CTCTTCTCT-------------------GAATATATCT------------------------------------------
--------------------------------------------------------------------------------
--------------------------------------------------------------------------------
---------GTTTT------------------------------------------------------------------
--------------------------------------------------------------------------------
--------------------------------------------------------------------------------
--------------------------------------------------------------------------------
--------------------------------------------------------------------------------
--------------------------------------------------------------------------------
--------------------------------------------------------------------------------
--------------------------------------------------------------------------------
--------------------------------------------------------------------------------
--------------------CCAC-CTTGAGAGAACCCATTTT-----AC-CTTGTGCCGAGTAAGAGCCACTTGATGGT
CCCTTAAGCTTATCTCAGAAGTCGTTTGTAATTCACTGAGAAACTGCTTAATCCAGACGGAGAAGGAAAACAGCAGCT
>Microtus agrestis
CAG----TTGTTTTTGTTGT-TGTTGTT----ACTGTTGGGGTTACCCTGCTG--------------------TTTT---
--------------------------------------------------------------------------------
---------------------------------------------------------------------ATCTGAT--AT
CTACCTGCCCAACACAGTGGCTGGGTCCATAAGTCCAATTATCAA-GCCATTTTTCATACAAACACAAAA---CCTAAAT
TGTTGC--------------------------------------------------------------------------
--------------------------------------------------------------------------------
--------------------------------------------------------------------------------
--------------------------------------------------------------------------------
--------------------------------------------------------------------------------
--------------------------------------------------------------------------------
--------------------------------------------------------------------------------
--------------------------------------------------------------------------------
--------------------------------------------------------------------------------
--------------------------------------------------------------------------------
--------------------------------------------------------------------------------
--------------------------------------------------------------------------------
--------------------------------------------------------------------------------
--------------------------------------------------------------------------------
------------------------------------------------------------------------------
>Nannospalax galili
CAG--------------------------TTTCTTACTGGAGTTTTCAACCT-TTTTACCT-GAAGTGA----TTTT---
--------------------------------------------------------------------------------
---------------------------------------------------------------------ATCTGTTTAAT
TTATTTGCATAACACATAGGCTGGACCTATATGTTCAATTATTAA-GTCAT-TTTCATATGAATAAAAAG----TTAAAT
TGCTGCCAAGTATGTTTC-AAAGTGGTGCA-------TCCCT-TT-TAATTCAA---------------------GAACT
CTGTTTTCG-------------------GAATAAATAT------------------------------------------
--------------------------------------------------------------------------------
--------------------------------------------------------------------------------
---------GCCTT------------------------------------------------------------------
--------------------------------------------------------------------------------
--------------------------------------------------------------------------------
--------------------------------------------------------------------------------
--------------------------------------------------------------------------------
--------------------------------------------------------------------------------
--------------------------------------------------------------------------------
--------------------------------------------------------------------------------
--------------------------------------------------------------------------------
--------------------CAGT-CTTGAGAG-AAACACTT------AC-CTTGTGCCGAGTAAGAGCCACCTGATGGT
CCATTAAGCTTATCTCTGAAGTAGTTTGTAGTTCACCAAGAAA-----------------------------------
>Jaculus jaculus
CAA--------------------------CTTCTTATTGGCATTTTCAATGTGTTTTACCT-GAAATAG----TTTC---
--------------------------------------------------------------------------------
---------------------------------------------------------------------ATTTGCTTAAT
TTATCTATATAACACATTGGCTAGACTGGTATATTCAATTATTAGGGTCAT-TTTCCTATGAATAAAATA----TTACAC
TGC-AGCAAGCGTGCTTT-CCAGTGTTGTG-------TCCCT-GTGTAACTCTGTCTTCTGGATAAGCTTGTCTTCAACT
CTGTTTTT--------------------ATTTTTATTTATTTGACAGCAACAGATAAAGGAAGAGGCAGATAGAGAGAAA
TAGAGAGAATGGGTGCGCCAGGGCCTCCAGCCACTGCAAACAAACTCCAGATGCATGCGCCCCCTTGTGCATCTGGCTAA
CATGGGTTCTGGGGAACCGAGCCTCGAACCAGGGTCCTTAGGTTTCACAGGCTAGCACTGAACCACTAAGCCACCTCTCC
AGCCCACTCGCCTT------------------------------------------------------------------
--------------------------------------------------------------------------------
--------------------------------------------------------------------------------
--------------------------------------------------------------------------------
--------------------------------------------------------------------------------
```


--------------------CAACGCTTAAAG--AGTCACTTT-----AC-CTTGTGCCGAGTCACCGCCGCTTGATGGT
CCGCCAAGCCGATCTCCGAGGTGGTCTGCAGTTCAGTGAGAAACAGCTTCAGCCACACGGAGATCGAAAGCAGCCGCT
>Anomalurus sp.
CAG--------------------------TTTCYTACTGTAGTT----GCCTATTKTAACT-GAAGTGG----TTTT---
----
---ATCTGCTCAAT
TTATCTGYATAACACATTGGCTAGACCCATGTGTTCAATTATTAA-ACCAT-TTTCACGTGAACAAAAWN----TTAAAT
TGCTGCCAAGTGTATTTT-TAAATGCTGCA-------TCCCT-TTGTAATTCAT---------------------GARCT
CTCCTTTCT-------------------GAACAAATTC--

---------TTTTT--

--------------------CAAT-CTTAAAAGAATCCACTTT-----AC-CTTGTGCTTAGTAAGAGCCACGTGGTGGT
CCACGAAGCTTATCTCAGAGGTAGTTTGTAATTCACTGAGAAACAGCTTAATCCAGACAGAAAAGGAAAGAAGCAACT
>Castor fiber
GAG--------------------------TTTCTTATTGTGCTT----ACCTATTTTATCT-GAAGTTT----TTTGTTG
GCTTGTTTGCTTTTTTGTTTTGAGA-CAGGGCTTTCCTTAT-------------AGCCCAGGGTGGCCTCCAATGCTCAT
TCCTCCTGCCTTAGCTTCCTGAGTTCTGGGATTACAGGCGTGCACCTGACTCTCCAAAG---TGGTTTTATCTGCTTAAT
TTATCTGTGTAACACATTGGCTGGACCCATATGTGCAATGATTAA-ACCAT-TTTCATGTGACTAAAA-G----TTAAAT
TGCCATCAAGTGTGTTTT-AAAATATTGCA-------TCCCT-CTGTAATTCAA---------------------GAACT
CTCTTTTCC-------------------AAATAAGTCT--

---------TTCTT--

--------------------CCAT-CTTAAAAGAACCCACTTT-----AC-CTTGTGCCGGGTAAGGGCCACTTGGTGAT
CCATTAAGCTTATCTCAGAGGTAGTTTGTAACTCACTGAGAAACAGCTTAATCCAGACAGAAAAAGAAAGCAGCAGCT
>Dipodomys ordii
AAG--------------------------TTTCTTATGGTGGTTAACTAACTACTTTACCC-GGAGGGT----TTTGGAT
GTT-------------GTTTTAAAAGCAACA-TCTCCCTATGTATCCCAGACAGAGCACAGACCGACCTCAAACTGACAA
TCATCCTGTTTAAACCTCCCAAGTGGTGGGATTACAGGCATGCACT--ACCATACCCAGTTCTCTGAGTAGCTGCTTCAC
TGATCTGCATAACG-ATTGGCTGGACCCATATGTTCAATGATTAA-CCCAC-TTTCAAATACATACAATA----TTAAA-
---CCA---------------------CAGCT
C---

--------------------AGTT-CGTACCGGGACACACTTT-----AC-CTTATGCCGAGTGTGAGCCACCTGATGGT
CCATTAAGCTGATCTCAGAAGTAGTCTGCAGTTCACTGAGAAACATCTTAATCCAGACGGAGAAGGAGAGCAGCAGTT
>Cavia porcellus
CAA--------------------------TTTTTAAATACAATT---AACGTATTTTATCT-AGAGTCG----TTTT---
----
---ATCTGCTTAGT
TTATCTGTAAAATACATTGTCAAGACCCAGGTGCTCAATTATTAA-AATAT-TTTCATGTGAATAAAAAG----TTAAAT
TACTGCCAAGTATTTTTTAAAAATG-T-CAAA-----TCCCT-TTGTAATTTAT---------------------GAGCC
CCCTATTAC-------------------AAATAAAAGT--

---------TTCTG--


```
--------------------------------------------------------------------------------
--------------------------------------------------------------------------------
--------------------------------------------------------------------------------
--------------------------------------------------------------------------------
--------------------------------------------------------------------------------
--------------------------------------------------------------------------------
--------------------TAATCCTTAAAGCATGT-ACTTC-----AC-CTTGTGCTGAGAGAGAGCCACTTGATGGT
CCATTATGCTGATCTCGGATGTCATTTGTAATTCACTGAGAAACTGCTTAATCCACGTGGAAAAGGAGAGGAGCAGCT
>Ictidomys tridecemlineatus
CAG--------------------------TTTCTTGTTGTATTT----CCCTATTCTGTCTTGAAGGGG----TTTT---
--------------------------------------------------------------------------------
---------------------------------------------------------------------ATCTGCTTAAT
TTATGTGTGTAAGAAATTGGCAGGACCCATATACTCGATCATTAA-AACAT-TTTCATATGAATGAAAAG----TTAGAT
TACTGTCACATATTTTCC-TTTGTAATTCAAGAACTCTCCCTTGTG-AAGGGAT--------------TTTTCTTCAA-T
CTTTTTTATTTCTGTTTAGAAC---TTACCCTAGGTGA------------------------------------------
--------------------------------------------------------------------------------
--------------------------------------------------------------------------------
---------CTCCATTTTGTTATTATTATTATTATTATTATTATTATTATTATTATTTTGTGATGCTGAGGATTGAACTC
AGGGCCTTATGCATGTGAGGCAAGCACTCTACCAACTGAGCTACATCCCCAGCCCTTGTTTTCTT---------------
--------------------------------------------------------------------------------
--------------------------------------------------------------------------------
--------------------------------------------------------------------------------
--------------------------------------------------------------------------------
--------------------------------------------------------------------------------
--------------------------------------------------------------------------------
--------------------------------------------------------------------------------
--------------------TAAT-CCTAACAGAAGA---TTT-----AC-CTTCTGCCGAGTAAAAGCTACTTGATGGT
CCATGACACTTATCTCAGAAGTAGATTGTAATTCACTGAGAAA-----------------------------------
```

Marker 69458

```
>ID4
--------------------------------------------------------------------------------
--------------------------------------------------------------------------------
--------------------------------------------------------------------------------
--------------------------------------------------------------------------------
--------------------------------------------------------------------------------
--------------------------------------------------------------------------------
--------------------------------------------------------------------------------
--------------------------------------------------------------------------------
--------------------------------------------------------------------------------
--------------------------------------------------------------------------------
------------------------------------------------------------------------TTTTTTT-
GCGGTG-CTGGGGATTGAACCCAGGGCCTC-GCGCATGCTAGGCANGCACTCTACCAC-TGAGCTACATCCCCAGCCCC-
--------------------------------------------------------------------------------
--------------------------------------------------------------------------------
--------------------------------------------------------------------------------
----------------------------------------------
>STRID1
--------------------------------------------------------------------------------
--------------------------------------------------------------------------------
--------------------------------------------------------------------------------
--------------------------------------------------------------------------------
--------------------------------------------------------------------------------
--------------------------------------------------------------------------------
--------------------------------------------------------------------------------
--------------------------------------------------------------------------------
--------------------------------------------------------------------------------
--------------------------------------------------------------------------------
--------------------------------------------------------------TTTTTTTTTTTTTTTTTG
GTGGTG-CTGGGGATTGAACCCAGGGCCTT-GTGCATGCGAGGCAAGCACTCTACCAACTGAGCTATATCCCCAGCCC--
--------------------------------------------------------------------------------
--------------------------------------------------------------------------------
--------------------------------------------------------------------------------
----------------------------------------------
>twinID-Spe
--------------------------------------------------------------------------------
--------------------------------------------------------------------------------
--------------------------------------------------------------------------------
--------------------------------------------------------------------------------
--------------------------------------------------------------------------------
--------------------------------------------------------------------------------
--------------------------------------------------------------------------------
--------------------------------------------------------------------------------
--------------------------------------------------------------------------------
--------------------------------------------------------------------------------
--------------------------------------------------------------------------TTTTTA
TGTGGTGCTGAGGAT-GAACCCAGGGCCTC-ACACATGCTAGGCAAGCACTCTACCACTTGAG-----------------
--------------------------------------------------------------------------------
--------------------------------------------------------------------------------
--------------------------------------------------------------------------------
----------------------------------------------
>Mus musculus
AACTCCAGGGCCTGGAAA-----GGTCCTAGAAATCCCCAGAGGTCTTTGCATCATACTTAAGAGAACTAC---------
--------------------------------------------------------------------------------
----------TGACC--GAAAACACTTTCC--ATAGCTCAG-CAGG--AAAAA-TGGAGCTTACCTCTCTCCCAAG--CC
TAACATA-CACCGCTTCAGCTATGAT-ATGCTCTGGC----GGTTGAAT-GAG----TTAACAAGCACAGCTACTTTATT
GGTAGCTGTG----AATGTATTTATGTTAT--------------------------------------------------
--------------------------------------------------------------------------------
--------------------------------------------------------------------------------
--------------------------------------------------------------------------------
--------------------------------------------------------------------------------
--------------------------------------------------------------------------------
--------------------------------------AGGAGG--CTGAT-TTCA-TTTGG------------------
--------------------------------------------------------------------------------
---TACCTACCTTAG-TGAGGCATCTGTCTGGTGAGGGAAATTAG----TTACCTTTACC--TGTTAGTCAACTCATAGT
C------GGGT-ATA-ACAGCCAGAGTGAAACCCTCAGAA-----------GCCAGAAGCCGTT---CTC----------
AGTGTGTG-TCATCACAGCAGTATG------------------------------------AGGGTGAGGAGGTCTA-GG
CAAGCAGTCTGT---GCAGTGTCTTCCCTTCCTGC-----AG-GGA
>Rattus norvegicus
AACTCC------TGAAAA-----GGTCCTGGAAATCCCCAGAGGCCTCTACATCCCACTTAAGAGAACTACGGGGTTGGG
GATTTAGCTCAGTGGTAGAGCGCTTGCCTAGGAAGTGCAAGGCCCTGGGTTCGGTCCCCAGCTCCGAAAAAAAGAAAAAA
AAAAAAAAAAGAAAAAAAAGAGAACTACTGACAGATCTCAG-CAGG--AGAAA-CATAGCTTTCCTCTCTCCCAAG--CC
TGACATA-CACCA-TTCATCTATGAT-AGGCCTTGGC----GGTTGAAT-GAG----TTAACAAGCACAATTA-TTTATG
GGCAACTGTG----ACTGTGTCTATGTTCT--------------------------------------------------
--------------------------------------------------------------------------------
--------------------------------------------------------------------------------
```

```
--------------------------------------------------------------------------------
--------------------------------------------------------------------------------
--------------------------------------------------------------------------------
--------------------------------------AAGAGG--CTGAT-TTTA-TTTGT------------------
--------------------------------------------------------------------------------
---TATCTACCTTTG-TGGGGTATCTGTCTGGTGAAGGGAATTAG----TTACCTTTACCTTCCTTAATCAACTGATAGT
T------GGATAATA-ACATCCAGAGTGAAACCCTCAAAA-----------GAC--AAGCCATT---CTT----------
AGTATGTGATCGTCTCAGGAGTATGAGATAC---AGCCC-------------------TGTAGGGTGAGGAGGTCTT-GG
AGAG--GTAGGTGCAGCGCCTCCATCCCTTCCTGC-----AG-GGA
>Apodemus sylvaticus
AACTCCTGGCCCTGGAAA-----GGTCCTGGAAATCCCCAGAGGTCTTTGCCTCACACTTAAGAGAACTAC---------
--------------------------------------------------------------------------------
----------TGACA--GAAAACACTGTAG------CTCAG-CAGG--AAAAA-TGTAGCTTGCCTCTCTCCCAAGCCTA
GCGTACA---CCACCTCAGATCTGGT-GGGCCTTGGC----AGTTGAAT-GAG----TTAGCAAGCACAGTTATTTTATT
GGCAGCCGTG----AATGTATTTACGTTAT--------------------------------------------------
--------------------------------------------------------------------------------
--------------------------------------------------------------------------------
--------------------------------------------------------------------------------
--------------------------------------------------------------------------------
--------------------------------------------------------------------------------
--------------------------------------AAGAGC---TGA--TTCA-TTTGG------------------
--------------------------------------------------------------------------------
---TACCTACCTTTG-TGAGGCATCTCCCTGGTGAGGAAAAGTAG----TTACCTTTACCTTCCTTAGTCAGCTCATAGT
T------GGATACTA-ACAGGC--AGAGAAACCTTCAAAA-----------GACAGAAGACATTATTCTC----------
AGTGTGTGGTCATCACAGAAGTGTGAGGTGC---AGCTG---------------------TGGGGTGAGGCGGTCTT-GG
CACG-AGGTGGT---GCAGCGTCTTCCCGCCCCGC-----AG-GGA
>Peromyscus maniculatus
AACTCCGGGTCCTGAAAG-----GGTCCTGGAATTCCCCAGAGGCCCTTGCGTCACACTTAAGAGAATTAC---------
--------------------------------------------------------------------------------
----------TGACACAGAAAACACGCTCC--ATAGCTGAG-CAGG--AGAAA-CGCAGCTTGCCTCTGTTC---GTGCC
TCATCTAACACCGCTTCGCATATGAT-GGGCCGTGGT----GGATGAAT-GAG----TCAACAAAC--AGTT-TTTAATT
GGCAGCTGTG----AAAATGC-TTTT---G--------------------------------------------------
--------------------------------------------------------------------------------
--------------------------------------------------------------------------------
--------------------------------------------------------------------------------
--------------------------------------------------------------------------------
--------------------------------------------------------------------------------
----------------------------------TTAGAAGAGG--TTGAT-TTCA-TTCAG------------------
--------------------------------------------------------------------------------
---CACCTACCTTTG-TTGGGCATTTTTCTAGTAAAGAAAATTAG----TTACCTTTA----CCTTAGTCAACTCGTAGT
T------AGTTAATA-ACA-CCTGAG-AAAACCTTCAAAA-----------GATGGAAGCCATT---CTC----------
AGTGTGTGCACATCCGAGGAGTTTGAGGTGC---AGGCC-------------------AGTGGAGCAAGTGGGTCTA-GG
CAGGAGGTAGGT---GCAGTGTCTTCCCTTCCTGC-----AG-GGA
>Microtus agrestis
AACTCCAGGTCCTCAAAG-----GATCCTGAAA-TCCCCAGAGGTCCTTGTGTCACACTTA-GAGAACTAC---------
--------------------------------------------------------------------------------
----------GAACACGGAGAGCATTTTCC--GTGGCAGAG-CAGG--AGAGG-TGTAGCTTGCCTCTGTCCCAAGTGCC
TCACATG---------------GGGT-GGGCCTTGGT----GCTTGAAT-GAG----TCAACCAACACAGTTACCCCATT
GGCAGCTACG----AAAGTTCTTAGATTAT--------------------------------------------------
--------------------------------------------------------------------------------
--------------------------------------------------------------------------------
--------------------------------------------------------------------------------
--------------------------------------------------------------------------------
--------------------------------------------------------------------------------
--------------------------------------AGGAGG--TTGAT-TTTA-TTCAG------------------
--------------------------------------------------------------------------------
---CACCCACCTTTG-TTGGGCATCTGTCTGGTAAAGAAAATCTG----TCACCTTTG----CCTTAGTCAACTC----T
T------AGAA--TA-GCAGCCTGAGGAAAACCCTCACAG-----------GACAG-AGCCGTT---CCC----------
AGTGTGTGGTCACTG-ATGAGCGTGAGGTGC---AGGTC-------------------------------TGGTCTA-GG
TGGGAGGTAGGT---GCAGTGTCTTCCTTTC-TGC-----AG-GGA
>Nannospalax galili
AACTCCAGATTCTGAAAGGAAAGGGTCCTAGAAATCCCCTGAGCTCCTTGCATCTCACTTAAGAGAACTGC---------
--------------------------------------------------------------------------------
----------TGATTCAGAAAACATTTTCCC-ATAGCTGAACCAGG--AGAAA-CGTCACTTATCTCTGTCCCAAGTGTG
TCACACA-CTGTTCTATGTATTAATAAGGGCCTCTGT----GTTTGAAT-GAG--CATG-TCA-ACAAATTTATTTTATT
GGCAACTATA----AA-GTA------TTAT--------------------------------------------------
--------------------------------------------------------------------------------
--------------------------------------------------------------------------------
--------------------------------------------------------------------------------
--------------------------------------------------------------------------------
--------------------------------------------------------------------------------
--------------------------------------AAGAGA--CCGAT-TTTA-TTCAG------------------
--------------------------------------------------------------------------------
---CCCCTGTCTTTA-TTGGGCATC----CAGTAAAGAAAAATAG----TT---TTTA----CCTTAGCCAATTCATA--
--AAGATAGATAATAGCCA-CCTGAGCAAAAATCCCAAAA-CC--TTGAAAGACAGAACCTACT---GTA----------
ATTGTCTGATCATGAAACATTTTTGAGTCTCTGTAGGCCAAGTCCATGCAGGTGAATTAGAAGGGTTAATCAGTCTA-GA
CAGGATAGAGGT---GCAGTGTCCTCCCTTCCTGC-----AG-GGA
>Pedetes capensis
```

```
AGCTCCACGTCCTAAAAA-----GGCCTTGGGAATCCCCAGAGATCCTTGCACTACACTTAA-AGAACTAC---------
--------------------------------------------------------------------------------
----------TGACTTAGAAAACTTTTTCCC-ATAGCTGAG-CTGGGGACAAGCTGTGTCTTATCTCTGTCCTTAGTACC
TCACACA-TTG--CTTGCTATGTGATGGGTGCTCTGTAAATGTTTGAAT-GAGTACATCAACAAACCAAAGTATTTTATT
GGAAGCTATCTTACTA-GTAACTTCATGAGTAGTTAGTTACACAGTAAG-------------------------------
--------------------------------------------------------------------------------
--------------------------------------------------------------------------------
--------------------------------------------------------------------------------
--------------------------------------------------------------------------------
--------------------------------------------------------------------------------
--------------------------------------AAGAGA--TT-ATATTCA-TTTAG------------------
--------------------------------------------------------------------------------
---CACACATCTTTATTTGGGCACT----TAGTGGAGAAAACTAGTAAGTTACCCTTA----CCTTAGCTGGTTAATAGT
TGAAGATAGATAATA-ACATCCAGAGCAATCAAGCAAGCA-----AAAATCCAGAGAACCAACT---CCC----------
AGTGTCTGATCGTCACT---------------GGAA-C------------------------------------------
----------------------------------------------
>Castor fiber
AACTCCATGTCCTGAAA------TGTCTTGGGAATCCCCAGAGGTCCTTCTACCACACTTAAGAG--CTAC---------
--------------------------------------------------------------------------------
----------TGACGCACA-AACTTTCCCCC-ATAACTAAGGCAGGG-AGAAACTGA----CATTGCTGTTCCTAGTACC
TCACACA-TTA--CTTGGCACATGATAGACACCTTGTAAATGTTTCAAT-CAGTGCAAAAACAAACAAAATTATTTTATT
GGAAACTACAT---AA-GTAATTCTGTGTG--------------------------------------------------
--------------------------------------------------------------------------------
--------------------------------------------------------------------------------
--------------------------------------------------------------------------------
--------------------------------------------------------------------------------
--------------------------------------------------------------------------------
--------------------------------------AAGAGG-GATTATTTTCA-TTCTG--TACCTATCTTTTTTTC
CTGGTA-CTGGGGATTGAACCCAGTGCCTCACACATGCT-AGGCAAGCACTCTAACACTTGAGGTGTG------------
---CACCAGTC-----TTTGGCACC----TAGTAGAGAAAATTAGTAAGTTATCCTTA----CCTTAGCTGGTTGGTAGT
TGAAGATAGATAATA-ACAGCATGAGTAATTAAGCAAAAA------------AAAGTACCTTCT---CCC----------
AGTCTCTGATCATCACACATTTCTGAGGCACTAGAG-CCAAGTCCAGGCAGGTGAATTGGGAGGATGAGTGGGTTTA-GA
CTGAGTATAGGT---ACAGTATCTTCCCTTCCTGC-----AG-GGT
>Dipodomys ordii
AACTGTAGGTCCTGAAA------GGTCTTGGAAAACCTTAGAGGTCCTTTGACTACCCTTAAGAG--CTAT---------
--------------------------------------------------------------------------------
----------TGACTTACAATTCCTTTCCCC-ATAGCAGAC-CTGGACAGAAGCTATAACTTATCCCTATCCCTGGTACC
TCAC-CA-TTG--CTTGGCATATTATAGGTACCTTGTAAATGTTTGAGTTGAATGCATCAACAA-CT-AGTTATTTTATT
AGAAACTATGT---AA-GTAATTAAGAGTGTCTAATCCTAGTTACTGAGGAGGATGAGATCTGAGGATGATAGTTTGAAG
CCAACATGGGCAGGAAAGTACTGAGACTTTTTTTTTTTTTGGTAATGGGGCTTGAACTCAGGGCCTGATCTCTTTTTCTC
AAGGCTAGTACTCTACTACTTGGAGCCACAGCCTCACTTCTGGGGGTTTTTTTGCTGGTGTTTTTTTTTTTTTCTTTTTG
GGTGGTTAGTTAGATGAGAGTCCTGTGGACTTTTTTTGCCCCAGCTGGCTTCAAATGGTAATCCTCAAATCTTAGCCTCC
TGAGTAACTAGGATTATAGGCGTGAGCCACTGGCACCTGGCCAGTATTAGGATTCTTTATCTCTAATTAACCACCAAAAA
AAACCAAAAAACAAAAACTGGAAGTGGAGATGTGGCTCAGGTGCTAGTCTTGATCAAAAATGTTCCAAGACAGGGCCCAG
GGCCCTAAGTTCAAGCCCCAGGACCAGCACACAAAAAAGAAAAAAAATAAACAAAA-GAAGGGATACCT-TCTTTCC-TC
CAAGCTTCTAGGGATTGAATCCAGGATCTTGCACATGCT-AGGCAAGTGCTCTACCACTTGAGGCATACCTCTACCCGTC
AGTCACCTGTCTTTA-TTGGGCACT-------TAGAGAAAATTAGTAAGTTATCCTTT----CCTTTGCTGGTTGATAGT
GGAAG---GTAAATA-GTGGGCTGAGCAAGCAAGTAAGCA------------AAA-AACCTTCT---CCC----------
AGTGTCTGATCATCAAATATTTCTGAGGCATGGGAC-CCAAGTCCAGACAGATGAATTGGAAGAATGAATAGGTCTA-GA
CAGGGTACAG-T---TCAGTGTCTTTCCTCCCTGC-----AG-AGA
>Cavia porcellus
AAGTCCGGGTGCTGAAAG-----GG-CCTGAAGCCTCCCAGGGGTCCTCACGCCA-GCTTGAGGT--CGGG---------
--------------------------------------------------------------------------------
----------TCACCTGTGA-TACTTCGCTC-AGAG---AG-------AGAGGCTATAGCTAATCACTGTCCCTAGTAGT
TCACACA-GTG--GTTGGCCAGT-----ACA-TCAGTAAATGTTTGAAT-GAGTAAGTCAACAAACAAAGTTATTTTATT
GGGAGCTACA------------AATATGGT--------------------------------------------------
--------------------------------------------------------------------------------
--------------------------------------------------------------------------------
--------------------------------------------------------------------------------
--------------------------------------------------------------------------------
--------------------------------------------------------------------------------
--------------------------------------AAGAAAAGATTGCTTTCA-TTTGG------------------
--------------------------------------------------------------------------------
---CACTTGTCTTTT-TTGGATATC----TCCTAGAGAAAATTAGTAAATTGTACTTA----CACTCACTAGTTGATAGT
TTGAGATGAATCAT--ATAGCCTGAGCAAGTAAGCAAAAA----AATG-TAAAAAGAGCCTTCT---CCTCTATTTAGAC
AGAGGGTC-TGATTAAACATATCTGAGACATTG-AGGCCAAGTCTAGAAGGGTGGGTTAGAAGGGTAA-TATGTCTACAA
-AGGGTGTAGGT---GCAGCATCTTCCTTCCTCAC-----AG-GGA
>Ictidomys tridecemlineatus
AACTCCTGGTCCGGAAAAG----GGTCTTGGAAATCCCCAGAGATCCTGGCACCACACTTAAGAA--CTAT---------
--------------------------------------------------------------------------------
----------TGACTGAACAGAACTTTCCCC-ACAGATGAA-CTGGG-AGAAACGGT---TCATCTCTGACCCTAGTACC
TCACACA-TTG--CTGGGCACATGATAGGCACTCAGTAAATGTCTGAAT-AGGTGAGTCAATGTACAAAGTTATTTTATT
GGAG-CTACAT---AA-GTGGTTACGTGGT--------------------------------------------------
--------------------------------------------------------------------------------
--------------------------------------------------------------------------------
--------------------------------------------------------------------------------
--------------------------------------------------------------------------------
--------------------------------------------------------------------------------
```

```
--------------------------------------AAGAAAAGATTTTTTTCCTTTTGG------------------
--------------------------------------------------------------------------------
---CACCTATTTTTA-TTGGTTGCC----AAATAGAGAAAATTAGGAAGTTGTCC-------TCTTAGCTAGTTGATAGT
TTT-GA--GATA----ACAGCCTAAGCAAGCAAGCAAAAAAACAAAAGACAAAAAGAACCTCCT---CCC----------
AGTGTGTGAGCATCAAACATTATTGAGGCGCTGGAG-CCGAGTTTAAGCGGGTGA-TTGGGAGTGTGCGTAGGTCTGCGA
CAGAGTACAGGT---GCAGTGTCTTTCCCTCCTGC-----AG-GGA
```

Mouse-related clade

Marker 20039

```
>PB1D10
---------------------------------------------------------------AGCCGGGCGTGGTGGCG
CACGCCTGTAATC----CCAGCACTCGGGAGGCTGAGGCAGGAGGAT----------CGTGAGT-TCAAGGCCAGCCTGG
GCTACATAG--CAAGA----------------------------------------------------------------
--------------------------------------CCCTGTCTCAAAAAAACAAAA---------------------
--------------------------------------------------------------------------------
--------------------------------------------------------------------------------
--------------------------------------------------------------------------------
--------------------------------------------------------------------------------
--------------------------------------------------------------------------------
----------------------------------------------------------
>Mus musculus
GAACTTTAAAATCTCTGGCTAAAGCACCTCTCTTTCCTGGTA-----------------------GGAC-CATGGAC--A
TGAG--TGCACTG----ACAGCCAGC-AGACACTTAGGTAACAGGATGCCAACCTGGGCTATGTAGCAAG---AACCTGG
TCTATCCAT--CAAGA----------------------------------------------------------------
--------------------------------------TCCTGTTTCAACATCTGCCCCAC-------------------
--------------------------------------------------------------------------------
--------------------------------------------------------------------------------
--------------------------------------------------------------------------------
----CCCACCCCCCAAAATAAG-GAGGGATAAAAA----GAGAAGCAATAATATCCATGAGGCCTCAGGGTGATGA----
---------------CAAGGGTACTTCATCTTTTACTCTGGGTGAA-TCTGGAA-TCCAGAGA---------TGCC-TGT
TCACTCTCCTTAGAGATGGCCAGGCCTC--------AGAGTGGGGTGGTACAGG-CGA
>Rattus norvegicus
GAACTTTAAATTCTCTGGCTAAAACATCTCTCCTGCCTAGTA-----------------------GGAC--ATGGGT---
-------GTACCT---GACAGCCAGC-AGCCACTTAAGTAACAGGATGTC-----------------------AACTTGT
GCTATCTAG--CAAGA----------------------------------------------------------------
--------------------------------------TCCTGTCTCAACATCTGCCCCAC-------------------
--------------------------------------------------------------------------------
--------------------------------------------------------------------------------
--------------------------------------------------------------------------------
----CCCAT-CCCTAAAATA-GAGAGGACAAAAAAGAGAGAGAAGCAATAATAACCACAAGGCCTCGGGGTGATGA----
---------------CAAGAGTACTTCATCGTTTCCTCTGGGCAAA-TCTGGAA-CCCAGACA---------TTCTG-AT
TCATTCTCCTGA------------------------GAGATGGCCAGGC-CTTA--GA
>Apodemus sylvaticus
GCACTTTAAAATCCTTGGCTAAGGTGTCTTTCCTTCCTGAAA-----------------------GGAT-CATGGAC--A
TGAG--TGCACCT---GACAGCCAGC-AGAC-CTTAGGTAACAGGGTGCC-----------------------AACCTGG
GCTATCTAG--CAAGA----------------------------------------------------------------
--------------------------------------ACCTGTCTCAACACCTGCCCCAC-------------------
--------------------------------------------------------------------------------
--------------------------------------------------------------------------------
--------------------------------------------------------------------------------
----CCTATCCCCAAAAATT-GAGAGGGGGAAAAAAAA-GAGAAGCAATAATATCCACGAGGCCTCGGGGTGATGA----
---------------CA-GAGTACTTCATCTTTTACTCTGGGTGAA-TCTGGAA-CCCAGGCA---------TTCCT-GT
TCACTCTCCTGAGAGATGGCCAGGCCTC--------AGAGTGAGGCAGTTCAGA-CGA
>Peromyscus maniculatus
CAGGACAAGACAGGGGAGCTAGGGCTTCTGTGTATCCTGCTTAGATTGGGGG----TGATATTAGCCAGGCATGGTGGTA
TATG--CGTATTG--TTTCAGCACTC--AAGGAGGCAGCAGAAGGATGGC----------AGGT-TCAAGACCAACCTGG
GCTATATAG--CAAGA----------------------------------------------------------------
--------------------------------------ACCTGTCTCAACAATTTTCCAAA-------------------
--------------------------------------------------------------------------------
-------------------------------------------------------AAGAAAGAGAGAGAGAGAAAGAGAG
GAGGGAGGGAGGGGGGAGGGAGGGAGGGAGGAAGGAAGGAAGGAAGGAAGGAAGGAAGGAAGGAAGGAAGGAAGGAAGGA
AGGAAGGAAGGAAGAAAAGAATAGAGAGAGAAAAAAAG-CAAAAGCAATAATATCCATGAGACCTCAGGTTGATGGG---
---------------CAAGAGTATTTCAGCTTTTACTCTAGATGAA-CCTGGAA-CCCAGAAA---------TCCCTTGT
TCACTCTCCTAAGAGAAGGCCAGGCCCCTGCCTCAGAGAGTGGGGTGGTGCAGA-TGA
>Microtus agrestis
GAACTTTAAAATCCCTGGCTAGAGCAGCTCTCCCTCCT------------AG----TGGAG--ACCTTG--AG-----CA
TGAG--TGCACCT---GACAGCCAGC-AGACCCTTACGTACCAGTCTGTC---------------CTTCCCCCACTCTGG
GAGATTCAGGACAAGACAGGGGAACTAGGGCCTCTGCACATCCTGCTCAGATTGAGGGTGATAGTAGCCAGGCATGGTGG
TACATACATATAGTCTCAGCACTCAAGGAGTCAAAAGATCCTGTCTCAACATCCTTCAAAA-------------------
--------------------------------------------------------------------------------
----------------------------------------------------------------------------AAAA
AAAGAATAGAATAAAACAAAGAGGAAACTCAACTATGTTCATAGCAGCATTGTTTGTCATANNNNNNNNNNNNNNNNNNN
NNNNNNNNNNNNNNNNNNNNNNNNNNNNATAGAGAGAGAGAGAAGCAATAATATCCACAAGGCCTCAGGATGATGGG---
---------------CAAGAGAATTTAAGTTTCTACTCTGGATGAA-TCTAGAA-CCCAGAAA---------TCCTTTGT
TTGCTGTCCTAGAAGAAGGCCAGGACCTTGGCTTCAAGAATAGGGTGGTGAACA-TGA
>Nannospalax galili
CA-GACAAGGCAAGGAGGTTG-AGCGTTTCTAGATTCTGCTCAGCTAGAAAG----TGGTATTAGCTGGGCTTGGTGGTA
GAGATCTGTAGTC----CCAGCATTC--AAAGAGGAAGTAGAAGGATGGC----------ATGT-TCAAGACCAACCTGG
GCTACGTAG--CAAAA----------------------------------------------------------------
--------------------------------------TCTTGGCTCAACATCTCCCATCC-------------------
--------------------------------------------------------------------------------
--------------------------------------------------------------------------------
--------------------------------------------------------------------------------
```

----------GAGAGAGAGAGA-----------------GAAAT-CAACAATATCCATGAGGCCTCAGAGTGATGGGCAA
GAAC--ACAGGATACTAGGAGTATTTCAGCTTTTACTCTGGAAGAA-ACTGGAA-CCCAGGAA---------TCCCTTGC
TCACTCTCCTGAGAGTAGGCCAGGCCTCTACCTC--AGAGTGTGGTGATGCAGAGGCA
>Jaculus jaculus
CAGAACAAGACAAGCAGACTGGAGCCTCCATGAATTCT---CAGATGGAAAG----TAAGCGTAACCAGGCATGGTGGTA
CAC-CACGTAGTCAGTCCTAGCACTTGAGAGGAAGACGCAGGAGGATGAT----------TTGC-TC-AGAC--------
-CTACATAG--GAAGA--
--------------------------------------TTCTG-TTCAAGAACTGAGAGATAGGGGCTGGAGAGATGGCC
TAGCAGTTAAGGTGTGTGCCTACGAAGCCTAAGAACCCATATTCAATTCTCCAGGTCCCATGTATACCAGATACACATGG
TGGCACATGCGTCTGGAGTTTGTTTGCAGTGGCTAGAGGCCCTGGTGTACTCATTCTCTCTCTCTCCCTCTCTCT-----
-----------------AATAAAAATAAATAAATAAAATCTTTTAAAAAGGAA--------------------CCAAGA-
GAGAAAGAGAGAGAGAGAGAGA-----------------GAAAA-CAGTAATACCCAGGAGGCCTCAGGGTGATGTACAG
GAAC--ATAAGACACTAAGAGTGCTTTAGATTTTA-TCTAA-GGAA-GCAGGGA-CCCAGGAA---------TCCCTTAC
TCGTTCTCTTAAGAATGGGCCAGGCCCCTACCTCAAAGAGAGTGGAGGTGCAGG-TGA
>Anomalurus sp.
--AGAGAAAGCAA-TAATATCAGCCAGGCATGGTGTTG
TACACCTGTAATT----CCAGCACTTGGGAGGCTGAGGCAGGAGGAT-------------------CACAAA--TTTGAA
GCCACATAG--TAAGA--
--------------------------------------CCCTATCACAAAAGAGAGAGAGA-------------------
----

------GAGAGAGAGAGAGAGA-GAGAGAGAGAGAGAGAGAAAG-TAATACTACTCATGAGGCCTCAGGACGATGGG--G
AAAC--ATGAGACACCAGGAGTGTCTCAGCTTTCACTGTAGGAGAA-GTTGGAA-CCTAGGCTGTCCACTGCTCATCAGC
ACACTCTCTTGAGGGTGGCCCAGGCCCC-ACCTCAAGGAGGGTGGTAGTACAGACTGT
>Castor fiber
CAGGACAAGGCAGACAGACAGG--CCTCTCTGGATTCTGCTCAGATAGAAAGCAG-TAATATTAGGTGGGTTTGGTGGT-
-ACACA-AT-------CCCAGCACTTGGGAGGTGGAGGCAGGTGG--------------------TCAAGGCCAGTCTGG
ACTACATAG--GGAGA--
--------------------------------------CCTTCTC-TAAAAACTAAAGAAAA------------------
----

------------AGAAAGATAAAAGAGAGAAAGGAAGGAAGAAGAGGAAAGGAGGGAGGGAAGAAAGGAGAAAAGTAAGA
AGGAAAGGAAGGAAGGGAGAAA-AGGAAGGAGGGAAAGAAA---------------------------------------
----
--------------------------------------GCGTGGTGGTGCAGAGTGG
>Dipodomys ordii
CAGGACCAGGCAGGCAGACAGG--CCCCTGTGAATTCTGCTCAGACAGAAAGCAGTTAATAATA--TTGG--TGGTGGCT
CACATCCGT-------CCTACCACCTGAGCGGTATAAACAAGAGGACTGA----------AAGTTTAAAGCC-AG-CTGG
GTGACACAG--GGAGA--
--------------------------------------CCC------AAAATTG---AAAA-------------------
----

---ACAAA
AGAAAGGAAGGGAGGGAGGGAG-GGAGGGCGGGCAAGATGGAAAGGAAGAATACCCGTGAGGCCTCAGGGCAATGGGCAG
GAACTTGGGAGAT-CCTAGAGTGTTTGAGCTTTCACTCTGGGAGAA-GTCGGGCCTCAGGCAATCTCC-TGTTTACAGGC
ACGCACTCCTGAGGATGGGCAAGGCCCTTATCTGGAGGAGGGTGGTGGTGCAGAGTGG
>Cavia porcellus
CAGCATGAGGCAGGCAGGCAGGGACCTCTCTGGATCCTGCTCATATAGAAAACTA-TAAGA-------------------
----

---TTCCTGAGGCCTTATGATGATGGGCGG
GAACC-CA-GGACATCAAGAGTGTCACAGCTTCTATTCTGGGAGAAAGCTGGAACCCCAGAAACCC--------------
---------------CTTGTGGGGCCCTC-CCTCGCAGAAGGTGGTAGTACAGCGTGG
>Heterocephalus glaber
CAGCACAAGGCAGGCAGACAGGGACCACTCTGGATTCTGCTCATATAGAAAGCCA-TAGTA-------------------
----

---TTCCTGAGGCCTTACAGTGATAGGCAG
GAACC-TG-GGACACCAGGAGTGTCACAGCTTCTACTTTGGAAGAAAGCTGGACCCACAG---TAACCCTGCTCACAGGC
AGGCCCTGTCGAGGATGGGCCAGGCCCCCACCTCATGGAGGGTGGTGGTGCAGAGTAG
>Ictidomys tridecemlineatus
CAGGACAATGCAGGCAGACAGGGGTCTCTCTGGATTCTGATCAGAGAGAAGGCAA-TGATG-------------------
----

---TCCATGAGGCCTCAGGGTGATAGG--G

GAAAC-AGAGGATACCAGGAGTGTTGTAGCTTTCACTCTGAGAGAAAGTTGGAAACTTGGTGATCTCCCTGCTCACTGGC
AGACTCTTCTGAGGCTGGGGTAGGCTCCTACCTTGAAGAAGGTGGTGTTGCAGAGTGG
>Marmota marmota
CAGGACAATGCAGGCAGACAGGGGTCTCCCTGGATTCTGATCAGAGAGAAAGCAA-TGATG-------------------
----

---TCCATGAGGCCTCAGGGTGATGGG---
GAAAC-AGAGGATACCAGGAGTGTTGTAGCTTTCACTCTGGGAGAAAGTTGGAAACTTGGTGATCTCCCTGCTCACTGGC
AGACTCTTCTGAGGCTGGGCTAGGCTCCTACCTTGAAGAAGGTGGTGTTGCAGAGTGG

Marker 16758

```
>ID_Spe
--------------------------------------------------------------------------------
--------------------------------TTTAAAAAAAAAGTTTAGTTGAAGATGGACACAATGCCTTTAT-----
-TTATTT-ATTTTTATGTGGTGCTGAGGATCAAACCCAGTGCCTCACACATGCTAGGCAAGTGCTCTACCACTGAGCTAC
AAC-CCCAG-CCC-------------------------------------------------------------------
--------------------------------------------------------------------------------
--------------------------------------------------------------------------------
--------------------------------------------------------------------------------
--------------------------------------------------------------------------------
--------------------------------------------------------------------------------
--------------------------------------------------------------------------------
--------------------------------------------------------------------------------
--------------------------------------------------------------------------------
-----------------------------------------------------------------
>ID4
--------------------------------------------------------------------------------
--------------------------------------------------------------------------------
---------TTTTTTTGCGGTGCTGGGGATTGAACCCAGGGCCTCGCGCATGCTAGGCANGCACTCTACCACTGAGCTAC
ATC-CCCAG-CCCC------------------------------------------------------------------
--------------------------------------------------------------------------------
--------------------------------------------------------------------------------
--------------------------------------------------------------------------------
--------------------------------------------------------------------------------
--------------------------------------------------------------------------------
--------------------------------------------------------------------------------
--------------------------------------------------------------------------------
--------------------------------------------------------------------------------
-----------------------------------------------------------------
>Mus musculus
AGGGCAGAGTGAGTCCTGTGGAGGACAAGACATGGCCTCC-CT-----GTAGGAGTCAAGACAGAGGTCAGACAAGGGTC
AGATGAGGAGGGGGCCCAGA-CAGAG----------AGTAATAAAAAGAATTACAAGCGTTAACAATTATTTTGAACAC-
----TTTTGTTTTGAGCCAGGGCTGGGGATCA--CCCAGGTTCTTGAA--TGCTACACAAGTGGTAAACCGTTGAGCTAT
ACC-CACGG-GCATTA-TTTTGTTATTTGTTA-CAGCAGACACT----GAAACACTTCACA-------------------
--------------------------------------------CTGAA-TTCTCAGAGTCGGGCCTGGGCAGTTCCAT-
---CCTTATGGTTACTTTCCCCTC--------------------------------------------------------
--------------------------------------------------------------------------------
-------------------ACTGAGAAAACTGAAGCCCAGGCAATG-TAAGTAACTTATCCAAA--TATCAGCCCACCCG
TGATAATGG-CTGAACTGTGTCTGCTGACTTCAGCACCCAGCTGTTTCCCACCTTCTTGTACAAGCCTGCCAGACT----
CTGTCACT-----ACCAGGCTTG---CCCCTGCAGCTGTGACTTCACGACACCGTGGGGCCAGCTGGA-------AATTC
CGGCT---AATTCCTTGCTAACAAGCCTGCCTGCC----TGTCGTTCCTTACAGCCCTACTCTGCTGACCATCTGTCTAG
CCGCCAC---TGGCCTACCTGG-TAACTGCTTGGCCC-----AC-CTGGTCCACAAAGCTGATGGCATCTCGGATGTTGA
GTCTGTAGTACACATCCCAGATCTGGTCGCGGATACGATAGGCTGACCGCCGCATCAAGAGCTGA
>Rattus norvegicus
AGGGTAGAGTGAGTCCCGCGGAGGACAAGACGTGGCCTCC-CT-----GTAAGAGTCAA----------------GGGTC
GGATGAGGAGTGG--CCAGG-CAGAGTAGGTAGACAGGTAATAGAAAGAATAGCAAGCATTAACAATTGTTTTGAACAC-
--TACTTTGTTTTGAGCCAGAGCCAGGGATTA--CCCAGGTTCTCGAA--TGCTATGCAAGTGATGAGCCATTGAGCTGC
GCC-CCACT-GCATTG-TTGTGTTCTTTGTTA-TAGCAGATACT----GAAACACTTCACACA-----------------
--------------------------------------------CTGAA-TTCTAAAGATCTGACCTAGGTAGGTAGTCC
TTTCCTTATCGTTACTTTCCTCTC--------------------------------------------------------
--------------------------------------------------------------------------------
-------------------GAAGAGGAAATGGAAGCCCAGATGATA-TAAGTAACTTATCCAAT--CATTGGCCTACCCA
CAGTAATAG-ATGAACTGTGTCTGT------CAGGGTCCAGCTGTTTCCCACCT-TCTGTCCCACCCTGCCAGACT----
CTGGCAAT-----ACT-GGCTTGTCACTCCTGCAGCTGTGACTTCA-----CCCTGGAGCCATCTGGG-------AACTC
CAACTCCCAGCCCCTCGCTAACAAGTCTGTCTGCC----TGTTACTCCTTACTGCCCTACTCTGCTGACCATCTGTCTGG
CAACCAC---TGGCCTACCTGG-AAACTGC-TGGCCC-----AC-CTGGTCCACAAAGCTGATGGCGTCTCGGATGTTGA
GCCTGTAGTACACATCCCAGATCTGGTCTCGGATACGATAGGCTGACCGCCGCATCAAGAGCTGG
>Apodemus sylvaticus
AGGGCAGAGTGAGCCCCGTGGAGGACAAGACGTGGCCTCC-CT-----GTAGGAGTCAAGATGGAGGTCAGACAAGGGTC
AGATGAGGAGAGA--CTGGC-CAGAG----------GGTAATGAAAAGAGTAACAAGCGTTAACAATTATTTTGAAC---
--TATTTTGTTTTGAG-------------------TCGGGTTCTCGAAAATGCTTTGCAAGTGATGAACCTTGGAGCTAC
GCC-CCAGG-CCATTA-TTTTATTTTTTGTTA-CAGCAGTTACT----GAAACACTTAACACA-----------------
--------------------------------------------CTTAA-TTCCCAAAACCTGACCTAGGCAG-TCCTAG
---CCTTATGGTTATGTTCCTCTC--------------------------------------------------------
--------------------------------------------------------------------------------
-------------------ATCTTGGAAACTGAAGCTCTGAAAATG-TCAGTAACTTAGCCAAG--CATCGACCTACCCA
CGATAAAAG-CTAAACCGTGTCTGTTGACTTCAGAGTCCAGCTGTTTCCCACCTTCTGGTACTG-CCTGCCAGACT----
GGGTCAAT-----ACCAGGCCTT---CTCCTGCAGCTGCGGCCTCA--------------CAGCTGGG-------ACCTC
CAGCTCCCTGCTCCCCGCTAACAGGCCCGCCAGCC----TGTCGTTCCTCCCTCCCCGACTCTGCTGGCCATCTGTCTAG
CGGCCAC---TGGCTCCCGTGGTTAACTGC-TGGCCC-----AC-CTGGTCCACAAAGCTGATGGCGTCCCGGATGTTGA
GTCTGTAG---------------------------------------------------------
>Cricetulus griseus
AGGGTAGAGTAAGTCCTGTGGAGGACAAGACGTGGCCTCC-CT-----GTAGGAGTCAAGACTGAGGTCAGACAAGGATC
AGATGAAGGG-GG--CCAGA-CAGAG----------GGTAATAAAGAGAATAACATGCATTAACAATTATTTCGATCAC-
TCTATTTTGTTTAGAGCGAGTGCCGGAGATTA--TTCAAGCTCTTAAA--C--TATGCAAGTACTGAACCATTGAGCTAC
AGC-TCCAG-GCATTA-TTTTGTTCT----------------------GAAACACTTCACATA-----------------
--------------------------------------------CTGAA-TTCTTAAAATCTAACCTAGGTACCACCTAT
```

```
---TCTTGTTGCTACTTTCTTCTC--------------------------------------------------------
--------------------------------------------------------------------------------
-------------------ACTGAGGAAACTGAAGCTCAAGGAAAA-TAAGTAACGTATATAAA--CATCAGCCTATACA
TGATAACAG-TTGAACTG--TCTGTTGACTCCAGAGTCCAGCTGCTTCCCACCTTTCTGTACTG-CCTGCCAGACT----
CTGTCAAC-----ACCAGGCTGG---CTCCAGCAGCTGTGACTTCACAACACCCCAGACTCAGCTGGG-------AATTC
CAACTCCCTGGCCCCTGCTAACAAGCCTGCCTGCC----TGTCATCCCTTACTGGCCTGCTCTGCTGACCACCTGTCTAT
CCACTGC---TGGCCTACCTGG-TAACTGC-TGGCCC-----AC-CTGGTCCACAAAACTGATGGCATCTCTGATGTTGA
GTCTGTAGTACACATCCCAGATCTGGTCTCTGATACGATAGGCTGACCGCCGCATCAACAGCTGA
>Mesocricetus auratus
AGGGTAGAGAAAGTCCTGTGGAGGACAAGACGTGGCCTCC-CT-----GTAGGAGTCAAGACCGAGGTCAGACAAGGATC
AGATGAAGG-GGG--CCAGA---------------------------GAATAACATGCATTAGCAATTACTCTAATCAT-
TCTATTTTGTTCTGAGAGAGTACTAGAGATTA--TTCAGGCTCTTAAA----CTATGCAAGTACTGAACCACTGAGATAC
AGC-CCCCG-GCATTA---------TTTGTTT-TACCAGATACT----GAAACACTTCACATA-----------------
--------------------------------------------CTGAA-TTCTTAAAATCTGGCCTAGGTAG-TCCTAT
---TCTTATTGCCACTTTCTTCTC--------------------------------------------------------
--------------------------------------------------------------------------------
-------------------ACAGAGGAAACGGAAGCTCAAAGAATA-TAAGTAATTTACATAAG--CATCAGTCTATGCA
TGATAACAG-TTGAACTGTGTCTGTGGACTCCAGAGTCCAGCTGTCTCCCACCTTTCTGTACTG-CCTGCTAGACT----
CTGTCAAC-----ACCAGGCTGG---CTCCAGCAGCTGTGACTTCACAACACCCCGGCGTCAGCTGGG-------AATTC
CAACTCCCTGGCCCCTGCTAACAAGCCTGCCTGCC----TGTCATCCCTTACTGGCTGGCTCTGCTGACCACCTGCCTAT
CCATTGC---TGGCCTACCTGG-TAACTGC-TGGCCC-----AC-CTGGTCCACAAAACTGATGGCATCTCTGATGTTGA
GTCTGTAGTACACATCCCAGATCTGGTCTCTGATACGATAGGCGGACCGCCGCATCAACAGCTGG
>Peromyscus maniculatus
AGGGCAGAGTGAGTCCTGCCGAGGACAAGACGTGGCCTCC-CT-----GTAGGAGTCAAGGTGGAGGTCAGGCAAGGATC
AGATGA----TTA-CCCAGGCCAGAG------AGAAGGTAATAAAGAGAATAACATGCGTTAACAATTATTTTGATGAC-
--TATTTTGCTTTGAGCC-GTGCTTAGGATTA--CTTGGGTTCTCCAA--CACTATGCAAGAGCTGAACCACTGAGCGAC
ACC-CCCAG-GCATTA-TTTTGTTCTTTGTCT-TACCAGATAC-----GAAACACTTCACCCA-----------------
--------------------------------------------CTGAA-TTCTTAAAATCGGACCTAGGTAG-TCCTAT
---TCTTATTGCTACTCTCTCCTC--------------------------------------------------------
--------------------------------------------------------------------------------
-------------------ACTGAGGGAACTGAAGCTCAGGCAGTG-TAAGTCGCTTATCTAAG--CATCGGCCTATACA
-----------TGAACTGTGTCTGTGGACTCCGGAGTCCAGCTGTTTCCCACCTTTCTGTACTG-CCTGTCACACT----
--GTCAAC-----AGCAGGCTGG---CTCCAGCAGCTGTGACTTTACAACCCTGTGGAGCCAGCTGGG-------AATTC
CAACTCCCTGGCCCCGGCTAACAAGCCTGCCTGCC----TGTCATCCCTTACTGCCCGGCTCTGCTGACCATCTGTCTGT
CCACTGC---TGGCCTACCTGG-TAACTGC-TGGCCC-----AC-CTGGTCCACAAAACTGATGGCATCTCGGATGTTGA
GTCTGTAGTACACATCCCAGATCTGGTCTCTGATTCGATAGGCTGACCGCCGCATCAACAGCTGA
>Microtus agrestis
AGGGTAGACCGAGTCCTGTGGAGGACAAAATGTGGCCTCC-CT-----GTGGGAGTCAAGATAGAGATCAGACAAGGATC
AGATGAAGG-TGG--CCAGA-CAGAG----------GGTAATAAAGAGAACAG--TGTGTGAAAAATTAGTTTGAACAC-
TCGATTTTGGTTTGAG------CTGGGGGCT---ACTCGGTTCTCAAA--CGCTATGGAAGTGCTGAGCCACCGAGCCAC
ACC-CCCAG-GCATCA-TTTTGTTCTTTGCTT-TACCACACACT----GAAACACTGCACATA-----------------
--------------------------------------------CTGAAATTCTTAAA--CCAACCTCGGTTG-TTCTAT
---TCTTGTTGCTGCTTTCTTCTC--------------------------------------------------------
--------------------------------------------------------------------------------
-------------------ACTGTAGAAACTGAAGCTCAGGCAATA-TAAGTAACATATCTAAG--CATTGGCCTCTACG
CGATAACAG-TTGAACTGTG--TGCTGACTCTAGAGTCCAGCT-TTCCCCACCTCTCTCTACTG-CCTGCCAGACTC---
CTGTCAAC-----ACCAGGCTGG---CTCTAGCAGCTGGGACCTCACAGCACCCTGGAGCCAGCTGGG-------AATGT
CAACTCTCTGGCCCCTGCTAACAAGCCTGCCTGCC----TGTCATCCCTGACTGCCCTGCTCTGCTGACCATCTGTCTAT
CC-CCGC---CGGCCTACCTGG-TAACTGC-TGGCCC-----AC-CTGGTCCACAAAACTGATGGCGTCTCGGATNNNNN
NNNNNNNNNNNNNNNNNNNNNNNNNNNNNCTGATACGATAGGCTGACCGCCGCATCAACAGCTGA
>Nannospalax galili
AGGGTAGAGTGAGGCCTGTGGAGGACAAAACGTGGCCTCC-CT-----GTAGGAGTCAAGACCAAGGTCAGATAGGGAGC
AGATGGAGGGTAG-CCCAGA-CAGTC------AGGGG--AAT--AGAGATTAACCCTTGTCAACAATTATTGTGATCAC-
-------TGTTTTGAGCCAGTGTTGGGGATTAAACCCAGGGTCTCACACATGCTGTGCAAATGCTGAATCAC--------
-AC-CCCAG-ACCTTA-TTTTGCTCTTTTGTT-GGCTGGATACT----GAAATACTTCATATA-----------------
--------------------------------------------CTGAA-TTCATAAAATCTGACATAGGTAG-TCTTAT
---TCTTGTTCCTGCTTTCTTTCTTTTTCTTTTTTTTTTTTTTTTTTTTTTTTTTTTTTGAGACAGGGTCTCCCTGCAGTG
GGGCTGGCCTTGAACTCACAGAGATCTACCTGCCTCTGCCTCCCAGGTGCTGGGATCAAAGGCGTGTGCCACCATGCCCA
GCATTCCTGCTTTCTTATCACTGAGGAAACTGAGGCTCAGGCAATA-----TGACTTAGCCTTG--CACCGGCCTGTTTG
TGATAACAG-TTGAACTATATCTGTTGACTCCAGAGTTCACCTGTTTCCTGCCTTTCTATACTG-TCTGCCAGACT----
CTGTCAACATCAGACCAGGCTGG---CTCCTGCAACTGTGATTTCATAGAACCATAGAACCAGCTGGG-------AATTC
CAATTCCCTGCCCTCGGCTAACAAGCCTACCTGCC----TGTCACCCCTTTCTGCCCTGCTCTGCTCACCATCTGTCTCT
CTGCCAC---TCGCCTACCTGG-TAACTGC-TGGCCC-----AC-CTGGTCCACGAAGCTGATGGCATCTCGGATGTTGA
GTCTGTAGTACACATCCCAGATCTGGTCCCGTATACGATAGGCTGATCGTCGCATCAACAGCTGA
>Jaculus jaculus
AGGGCAGAGTGAGGCCTGCAGAGGACAAGATGTGGCCTCC-CT-----GTAAGAGTCAAGATTGAGATCAG-------GA
AGGTGAATAGTGG-CCCAGA-CGTTC------AGAGGACAATCAAGAGAATAACAT----TAGCAATTATTTTGATTAT-
TTTGTTTTGCTTTTAGCCAGTGCTGAGAATTAAACCCAGGGCCTCACACATGCTAGGTAAG-----TATTACTGAGCTAT
ACC-CTCAG-GCCTTA-TTTTGTTCTTTTGCTGTGCCAGGTACT----GACACACTT-ACA-------------------
--------------------------------------------CTGAA-TTTGCAGAGTCCAACATAGGTAC-C-----
----TTTATTCCTGCTTTCTTATC--------------------------------------------------------
--------------------------------------------------------------------------------
-------------------ACTAAGGAAACTGAGGCTCAGGCAATA-TAAGTGACTTACCCAAGGTCACAAGCTTGTAAG
TACTAATAG-TTGAACCGCATCTGCTGACTCCAGAGTCCAGCTG-TTCCCACCTTTCTGTACTG-CCTGCCAGACT--GA
TTGTCAACACCAGGCCAGGCTGG---TTCCTGCAGCTGTGACTTCACAAGACCAACGAGCCAGCTGGG-------AATTC
CAATTCCCTGCTCGATGCTAACAAGCCTGCCTGCC----TGACATCCAT----------CTCTGCTGACCATCTGTTCAC
CTACCAC---TGGCCTTCCTGG-TAACTGC-CGGCCT-----AC-CTGGTCCACAAAGCTGATGGCATCGCGGATGTTCA
GTCTGTAGTACACATCCCAGATCTGGTCCCGGATCCGGTAGGCTGACCGCCGCATCAGCAGCTGA
```

```
>Castor fiber
AAGGCAGAGTGAGGCCTGTGGAGGACAAGATGTGGCCTCC-CT-----GCAGG-------ACCAAGGTCAGACA----TC
AATTAGAGGGTGG-CCCAGA-C----------AGAGGGTAATAAAGAGCATAACAATAGTTCATACTTTTC---------
---TTTTGGTTTTTTTATAGTGCT-------AAACCCAGGGCCTCGTGTATCCTAGATAAGTGCTCTACTACTGAGCTAC
ACCTCCCAGCCCCTTA-TTTTGCTCTTTTGATGT-CCAGGTACTGTGAGAAACACTTTACATA-----------------
--------------------------------------------CTAAA-GTCACAAAATCTGACATAGGTGC-TCTTA-
----CTTATTCCTGCTTCCT--CC--------------------------------------------------------
--------------------------------------------------------------------------------
-------------------ATTGAGAAAAATGAGGCTCAGACAATG-TAAGTTACTTACCTAAGGTCACAGGCCTGTAAT
TAGTAACAGTTTGACCCATAAATGCTGACTCCAGAGTCCAGCTGTTTCCCACCTTTCTGTACTG-CCTGCAAGACTCTGA
CTGTTAG-ACTTGGCCAGGTTTG---TTCATGCAGCTGCCACTTCACAAGACTGTGAAGACAAATGGG-------AGTTT
CTAAC------TCCCTGCTAACAAGCCTGCCTGCC----TATGGCCCCTTTCTGCCCTGTTCTGC---------------
----------AGGCCCACCTGG-TAACTGC-TGGCTC-----AC-CTGATCCACAAAGCTGATGGCATCCCGGATGTTGA
GTCTGTAGTACACATCCCAGATCTGGTCCCGGATGCGGTAGGCTGACCTCCGCATCAGCAACTGA
>Dipodomys ordii
AGGGCAGAGTGAGACCTGTGGAGGACAGGACGTGACCTCC-CT-----GCAGGGGTCAAGACCGAGATCAGACAGCAATC
----AGAGGACGG-CCCAGA---------------AAGTAAGAAAAAGAAGAAGAATATCTCACACGTATTTTACTTTTT
TTTGTTTTGCTTT---GTAGTTCAGCAGATCAAAACCAGAGCCTCAGGCCTGCTAGGTAAGCAC------ATTCAG--AG
ACC-CCCAGCGCCTTATTTTTGCT-----------CCAGATAA-GTATGAGACACTTTATATT-----------------
---------------------------------------------TAGAATTCACAAAATCTGACACAGGTGC-TCTTAT
TATTCTTACGCCTGCTTTCTTATC--------------------------------------------------------
--------------------------------------------------------------------------------
-------------------ACTGAAGAAAAT-AGGCTGAGACAAGGTTAAGTTACTTACC-AAGGTCACAAGCCTATAAG
TGGTAACCGTTTGAGCCACATCTGCTAGCTC--GAGTCCAGCTATGTCCCACATTTCTGTACTG-TCTGCCAGACT--AA
CTG-----------------------------CAGCTGCCACTTCATATGACCATAAAGTCAACTGGG-------AGTTC
CCAAT------TGTCTGCTAACAAGCCTGCCTGCT----TATTGCCCCTTTCTGCCCTGCTCTTCTGGCTCCATGTCCTC
CCACCATAAGTGACCCACCTGG-TAACTGC-TGACCT-----AC-CTGGTCCACAAAGCTGATAGCATCCCGGATGTTAA
GTCTGTAGTATACATCCCAGATCTGGTCCCGGATGCGGTAGGCTGACCGACGCATTAGCAGCTGA
>Cavia porcellus
AGGGCAGAGTGAGGCCTGTGGAGGACAAGACATGGCCTCC-CT-----GCAGAGGTCAAGACCAAAGTCAGATAGCAGTC
AAATGGAGGGTGG-CCCAGA-----------TAGAGGGCAATAAAAATAACAATAA------------------------
--------------------------------------------------------------------------------
------------CTTA-CTTTGCACTTTTTATATGCCAGATACTGT-AAAAATACTTTACATAGTCTAACATAGATACTT
GTCTATGTTAATACTTTACATAGTCTAACATAGATACTTGTAACATAGA-TT-ACAAAGTCTAACATAGATAC-T--TGT
-------ATTCCAGCTTTCTTATTATG-----------------------------------------------------
--------------------------------------------------------------------------------
-------------------GCTAAGAAAACTGAGTCTCGCACAAGG-TAAGTGATTTACCCAAGGTC-CAAGCCTGTAAA
TGGTAACAC-TTGAATCACATCTGCTGACTGCAGAGTCCAGCTGTTTCCCATCTTTCTGTACCA-CCTGCCAGACTCTCT
ATATGGGC-CCAAGCCAGGCTGG---TTCCTGCA--------CCCAC----CTATGGAGCCAACTGGG-------AATTC
CCTAC------TCCCTGCTTACAATCCTGCCTACC----TGTTGTCCCTTT----CCTGCTCTGCTGGCCATTTTCCCAC
CCACCGC------------------------TGGCCC-----AC-CTGGTCCACGAAGCTGATGGCATCCCGGATGTTGA
GCCTGTAGTATACATCCCAGATCTGGTCCCGGATGCGGTAGGCTGACCGCCGCATCAGCAGCTGA
>Heterocephalus glaber
AGGGCAGAGTGAGGCCTGTAGAGGACAAGACGTGGCCTCC-CT-----GTAGGGGTCAAGACCAAAGTCAGATAGCAATC
AGATGGAGGGTGG-CCCAGA-CAGT------CAGAGGGTAATAAAAATAACAATAACTAA--------------------
--------------------------------------------------------------------------------
------------CTTA-TTTTGCTCTTTTTATGTGCTAGATCCTGT-AAAAATACTATATGTA-----------------
--------------------------------------------TTAAA-TCTACAAAATCTAACATAGGTAC-TATTGT
---------TCCTGCTTTCTTACA--------------------------------------------------------
--------------------------------------------------------------------------------
-------------------GCTGAGAAAACTGAGTCTCACACAATA-CAAGTGACTTACCTAAGGTCACAAGCCTGTAAA
TG-TAACAC-TGGAACCGCATCTGCTGAATGCAGAGTCCTGCTGTTTCCCACCTTTCTGTACTA-CCTGGCAGATTCTCA
ATATGAGCCCCAAGCTAGGCTGG---TTCCTGCA--------CCCAC----CTATGGAGCCAGGTGGGAATTCCCAACTC
CCTGC------TCCCTGCTTACAAGCCTGCCTACC----TATTGTCTCTTT----CCTGCTCCACTGGTCATCTTCCCAC
CCACCAC------------------------TGGCCC-----AC-CTGGTCCACGAAGCTGATGGCATCCCGGATGTTGA
GTCTGTAGTACACATCCCAGATTTGGTCCCGGATACGATAGGCTGACCTCCGCATCAACAGCTGA
>Ictidomys tridecemlineatus
AGGGCAGAGTGAGGCCTGTGGAGGACAAGACATGGCCTCC-CT-----GTAGGGGTCAAGACCAAAGTCAGACAGCAATC
AGATGGAAGGTGG-CCCACA-GCATC-----AAGAGAATAATAAAGAGAACAATAGTTAATA------------------
--------------------------------------------------------------------------------
------------CTTC-TTTTGCTCTTTTGATGTGCTAGATACTGT-AGTAACACTTTATATA-----------------
--------------------------------------------TTGAA-TCCGTAAAACCTGAAATAGGTAC-TCTTGT
---TCTTATTTCTGCTTTCTTATG--------------------------------------------------------
--------------------------------------------------------------------------------
-------------------ACTTAGGAAA-T-AGGCTCAGAC-ATA-TAAGTGGCTTCCA-AAAATCACACACCTGTGA-
---TAATAG-TTGAACTACATCTGTTGACTCCAGGGTCCAAATGTTTCCCACCTTTCTATACTG-CTTGCCAGATTCTGA
ATGTCAGCACCAGGTCAAGCTTA---ATTCTGCT------ACTTCATCAGACCATGAGTACAGCTGGG-------AACTC
CCAAC------TCTCTCCTAATAAGGCTGCCTGCCTGCCTGTTGCTCCTTTTTATCCTGTTCTCTCGGCCATATGC----
----------------------------------CCT-----AC-CTGGTCCACGAAGCTGATGGCATCCCGGATATTGA
GTCTGTAGTATACATCCCAGATCTGGTCCCGGATGCGGTAGGCTGACCGCCGCATCAGCAACTGA
>Marmota marmota
AGGGCAGAGTGAGGCCTGTGGAGGACAAGACATGGCCTCC-CT-----GTAGGGGTCAAGACCAAAGTCAGACAGCAATC
AGATGGAAGGTGG-CCCACA-GCATC-----AAGAGAATAATAAAGAGAACAATAGTTAATA------------------
--------------------------------------------------------------------------------
------------CTTC-TTTTGCTGTTTTGATGTGCTAGATACTGT-AGTAACACTTTATATA-----------------
--------------------------------------------TTGAA-TCCATAAAACCTGACATAGGTAC-TCTTGT
---TCTTATTTCTGCTTTCTTATG--------------------------------------------------------
--------------------------------------------------------------------------------
```

-------------------ACTGAGGAAAC--AGGCTCAGAC-ATA-TAAGTGGCTTCCA-AAAATCACACACCTGTGA-
---TAATAG-TTGAACTACATCTGTTGACTCAAGGGTCCAGATGTTTCCCACCTTTCTATACTG-CTTGCCAGATTCTGA
ATGTCAGCACTAGGTCAAGCTTA---ATTCTGCT------ACTTCATCAGACCATGAGTACAGCTGGG-------AACTC
CCAAC------TCTCTCCTAATAAGGCTGCCTGCCTGCCTGTTGCTCCTTTCTATCCTGCTTTCTCGGCCATATGC----
----------------------------------CCT-----AC-CTGGTCCACGAAGCTGATGGCATCCCGGATATTGA
GTCTGTAGTATACATCCCAGATCTGGTCCCGGATGCGGTAGGCTGACCGCCGCATCAGCAGCTGA

Marker 93865

```
>PB1D10
--------------------------------------------------------------------------------
--------------------------------------------------------------------------------
--------------------------------------------------------------------------------
--------------------------------------------------------------------------------
--------------------------------------------------------------------------------
--------------------------------------------------------------------------------
--------------------------------------------------------------------------------
--------------------------------------------------------------------------------
--------------------------------------------------------------------------------
--------------------------------------------------------------------------------
--------------------------------------------------------------------------------
----AGCCGGGCGTG---GTGGC-GCACGCCTGTA---------------------------------------------
------------------------------------------------------ATCCC-----AGCACTCGGGAGGCTG
AGGCAGGAGGAT-CGTGAG-TTCAAGGCCAG-CCTGG-GC----TACATAGCAAGACCCTGTCT--CAAAAAAA---CAA
AA------------------------------------------------------------------------------
--------------------------------------------------------------------------------
------------------
>Mus musculus
ACCAGGCCTCTACCCA-AGGGACCAAACCAGGATTGGAGCCATGTCCGGTGTCTACCATTAGGCAAC-GGTAGGGAACCA
GACTGCTGAAAG-G----------------GC-TATAACTCATCCTG--GTATCACAGAACCCCACATCA--CAGTGAGG
TCAGAAGATAGGCTCCCCGGTCAACTCCATAAAAAAAAAGTTCTCCCCGT-AGCTGAACCGATGAACTCAAGGAAGAAAA
-------CCCCAT----CTATTCCCAAGCACCAAAAACAGCACCTGGCCCATGTGAC-----------------------
-----------------TGGGTGCATGGCTGG-ATGGATAGCAGGCCTGCAGGAAGACACAGGACTCTAAGAAAGGAA--
--------------------------------------------------------------------------------
--------------------------------------GGAGTCCTTGCCTGAGATCTAACAGGGCTCCTCTCACT----
--------------------------------------------------------------------------------
-----------------------------------------------------------------------TCTTAG---
--------------------------------------------------------------------------------
--------------------------------------------------------------------------------
--------------------------------------------------------------------------------
---------------------------------------------------------CCTGTA-ATCACACAGGAAGCAG
AGGCAGGAGGATGAGGCACG--CAAGGCCAG-CTTGG-GC----TATTCAGCATGAA--TTT----CAAAGAAA---TGA
AAAC-TGAAATCTTTGAATCGAATTAGATACCAGTTGATCAAATCCCCAAAGCACTAATTTTCC-TGC----ACACA---
--TAGAAGAGG----TGTGTAAAGCCCACACCCTTGGACAGGGG-A-TCTGAAGTTAACA--CGTGA----GTTTTGAAA
GTTACTCTTT--GGTAGT
>Rattus norvegicus
ACCAGACCACTACCCA-AAAAACCAAATCAGGGTTACAGCCATGCCCAGTGTCTACCATTAGGCAAC-GGTAGGGAACCC
GAGTGCTGA-AG-G----------------GC-TGTGACTCACCATG--GCTTCACGGAAGCCCACATCA--CAGTGAGG
CCAAGAGATAGGCTCTTCAGTCAACTCCATAAAA----AGTTCTCCCAGT-AGCTGAACCGATGAACTCAAGGAGGAAA-
------CCCCCAC----CCATTCCC-AGCACC------------------------------------------------
-----------------TGGATGCATGAATGG-ATGGATAGCAGGCAGGCAGGAAGATGTGGGGCTCTAAGAAAGGAA--
--------------------------------------------------------------------------------
------------------------------------GTGGAGTCCTAGCCTGAGATCTATCAAGGTTCCTCTTACT----
--------------------------------------------------------------------------------
-----------------------------------------------------------------------TCTTTG---
--------------------------------------------------------------------------------
--------------------------------------------------------------------------------
--------------------------------------------------------------------------------
---------------------------------------------------------CCTGCA-ATCACACAGGAAGCAG
AGGCAGGAGGATGAGGCATG--CAAGGCCAG-CTTGG-GC----TATTCAGCATGAA--TTT----CCAAGAAA---TGA
AAAC-TGAAATCTTTGAATCGAATTAGATACCTGTTTATCAAATCCCCCAAACACTAATTTTCC-TGT----GGCC----
-----------------CATAATGCATGCACCTTTGGACAGGGG-AATCTGAAGTTAACA--CGTGA----GTTTTGAAA
GTTACTCTCT--GGTAGT
>Apodemus sylvaticus
--------------------------------------------------------------------------------
------------------------------------------------------------------------------GG
TCAGGAGATAGGCTCCCCAGTCAATTCCATAAGA----AGTTCTCTGAGG-AGCTGAACCGATGAACTCAAGAAACA---
--------CCGAT----GTATTCCCAGGCACTAAAACCAGCACCTGGCAAATGTGAC-----------------------
-----------------TGGGTGCATGGATGG-ATGGATAGCAGGCATGCAGGAAGACACAGGACTCTAAGAAAGGAA--
--------------------------------------------------------------------------------
------------------------------------GTGGTGTCCTAGCCTGAGATCTATCAGGGTTCCTCTCTCT----
--------------------------------------------------------------------------------
-----------------------------------------------------------------------TCTTTG---
--------------------------------------------------------------------------------
--------------------------------------------------------------------------------
--------------------------------------------------------------------------------
---------------------------------------------------------CCTGTA-ATCACACAGGAAGCAG
AGGCAGGAGGATGGATGATG--CATGCAGGG-CCAGC-TTGGGCTATT-ATTCAGCA--TGAATTTCAAAGAAC---TAA
AAAC-TGAAATCTTTGAGTTGGATTAGATACCAGTTTATCAAATCCCCAAAGCACCAATTTTCC-TGC----AGTC----
-----------------CAGAATGCACCAACCCGTGAACAGGGG-ACTCTGAAGTTAACA--CGTGA-------------
------------------
>Cricetulus griseus
ACCAGACCTCTACCCC-AGGGACCCAGTCCATGTGAGGGCCATCTCCAGT------------------GGTAGAAAAGCA
GAGTGCTGAAAG-GCAACAGCTTCCCATATGC-CCTGACTCATCACATCACA----------------CAGCCAGTATGG
TCAGGAGCTAGGTTCCCCAGTGGACTTTCTAAGT----AGTTCTCCCAGT--GCTAAACTAATGAACTCAGGGAAGAAAG
```

AAA---CCCTCAC----CCGTTCC-AATTCCCACAACCAATAACTGGCCCATGGGATT----------------------
-----------------TGGGTGCATGGATGG-ATGGATAGCAGGCAAGCAGGAAGACACAGGACTCTAA----GGCA--
----
------------------------------------GTGGTGT---------AGATCTATCATGACTCCTCTTACATCTT
TACATTTTATTTATTTA----TTTTGGTTTTCTGAGACAGGGTTTCTCTGTATAGCTTTGTAGCCTGTCCTAGCATTCAC
TCTGTAGATCTATTTGCCTCTGCCTCCCAAGTGCTGGGATTAAAGGCATGCACCATCTCCTCCTGGATATGCCCTTAAAT
TTCAAAAATAAATGCAGCGGGGCAGTGGTGGTGTATGCTTTTAATCCCAGCACTCGGGAGGCAGAGGCAGGTGGATCTCT
GTGAGTTCGAGGCCAGCCTGGTCTA--------CAAACAGAAACCCTGTCTCAAAAAACCAAAATAATAATAATAAAAAA
AG--
---CCTGTATATCACACACAAAGCAG
AGGCAAGAGGATGAGGCATG--CAAGGCCAG-CTTGG-AC----TGTTTAGCATGAA--TTTCTTTCAAAGAAA---GGA
AAAC-TGAAATT-TTAAATTGAATTCAGTATCAGTTTATCAAATTCCCAAAGCACTAGTTTTCACTGC----AG------
---------------------------ACAACTTTGGATGGGGA-AATCAAAAGTTAACA--TGTGAC-AAGCTTCCCAC
ATGAGT-TTC--TAAAGT
>Mesocricetus auratus
ACCAGACCTCTACCCCGGGGGACCGAATCCGGGTGAGAGCCATCTCCAGT------------------GGTCGGAAAGCA
GAGTGCTGAAAG-GTGACAGCTTCCCATATGG-CCCGGCTCATCACATCATG----------------CAGCCAGTGTGG
TCAGGAGCTAGGTTCCCCGGTGGACTTTCTAAGT----AGTACTCCCAGT--GCTAAACTAATGAACTCAGGGAAGAAAG
AAA---CCCTCAC----CCGTTCC-AATTCCCACAACCAACACCTGG---------------------------------
--------------------GTGCATGGATGG-ATGGATAGCAGGCAGGCAGGAAGACACAGGGCTCTAA----GGCA--
----
------------------------------------GTGAAGTCT-------AGATCTATCATGGCTCCTCTTACATCTT
TAAATTTTATTTATTTATTTATTTTGGTTTTTTGAAACAGGGTTTCTCTGTATAGATTTGGAGCCTGTCCTGGCATTCAC
TCTGTAGATCCACCTGCCTCTGCCTCCCAAGTGCTGGGATGAAAGGTGTGCACCACCACTTCCTGGCTACGCCTTTAAAT
T-AAAAAAAAAATGCAGCCCAGCAGTGGTGGCACATGTGTTTCATCCCAGCACTCGGGAGGCAGAGGCAGGTGGATCTCT
GTGAGTTCCAGGCCAGCCTGGTTTATAAAACTACACAGAGAAACCATGTCTCAAAAAATAAAAATAAA------AAATAA

--ATGCCTGTATATCACACAGAAAGCAA
AGGCAGGAGGATGAGGCATG--CAAGGCCAG-CTTGG-GC----TGTTTAGCATGAA--TTTCTTTCAAAGAAA---GGA
AAAC-TGAAGTT-TTTAATGGAATTTAACATCAGTTTATCAAATTCTGAAAGCACTAGTCTTCACTGC----AGCC----
-----------------CATAATGCACACAACTTTGGATGGGGG-AATCAATAGTTAACA--TGTGAC-AAGCTTCCCAC
CTGAGT-TTC--AAAATT
>Peromyscus maniculatus
ACCAGGCCTCTACCCC-AGGGACCAAATCAGGT------CCATCTCCAGTGTTTACTATCAGGCAGT-GGTAGGACACCA
GAGTGCTGAAAG-GCGACAGCTTCCCATATGCCCGTGACTCATCACATCATGC---------------------------
-----AGCTAGGATCCCCAGTCAGCTTTATAAGT----AGTTCTCCCAGT-AGCTAAACTGATGAACTCAAGGAAGAAAG
AAA---CCCCCAC----CCATCCCTAGTCACCACAGCCAACACCTGGCCCATGGGATTGGATGGATGGATGGATGGATGG
ATGGATGGATGGATGGATGGATGGATGGGTGG-ATGGATAGCAGACAGGCAGAAAGAAACAGGACTGTAA----GGAA--
----
------------------------------------GTAGAGTTT-AGCCTAGGATCTATCATGGCTCTTC---------
----
---TTTAAAT
TTAAAAA---
--AAAAAA
AA--
--ATGCCTCTATATCACACAGGAAGCAG
AGGCAGAAGGATGAGGCATG--CAAGGCCAG-CTTGG-GC----TATTCAGCATGAA--TTT----CAAAACAAA--TGA
AAAC-TGAAATTTTGGAATTGAATTCAGTATCAGTTTATCAAATTCCCAAAGCATTAATTTTTGCTGC----AGCC----
-----------------CATAATGCACACACCTTTGGACAGGGGGAATCATAAGTTAACA--TGTGAC-AAGCTCCCCAC
ATGAGT-TTC--GAAAGT
>Microtus agrestis
ACCAGGCTTCTACCCC-AGGGACCCGACCAGGGTTAGACCCATCTCCCGTGTTTACCATCCAGCAGC--------AGCAG
GAAACCAGAGTGCTGGACAGCTTCGCATGTGC-TCTGACTCATGATG-----------------TCATACAGCAGTGTGG
TCAGGAGCTAAGATCCCCATT-GACTTTGTGAGT----AGTTCTTCCAGT-ACCTATACTGATGAACTCAAGGAAGAAAC
AAA---CCCC-AC----CCATT------CACCACAACCAACACCTGGCCCATGGCAC-----------------------
-------------------------TGGGTGC-ACGGGTGGAGAGCAGGCAGGAAGACACAGGACTCTAC----GGAA--
----
------------------------------------GTGGAGACT-AGCCTGAGATCTGTCAT---TCCTTTTACG----
----
---TCTTTAAGC
TTAAAAA---
--AAA---

--ATGCCTGTGTATCACACAGGAAGCAG
AGGCAGGAGGATGAGGCCTG--CAAGACCAGGCTTGG-GC----TAGTCAGCATGAA--TTTCTTTCAAAGAAA---CAA
AACC--AAATTTTTTTAATTGAATTCAGTAACAGGTTATCAAATTCCCAGAGCACTAATTTTCACTGC----AGCC----
-----------------CATAATGCACACATC-ACGGACAGGGG-AATCAGAAGTTAAGA--TGTGAC-AAGCTCCCCAC
ATGTGT-CTC--AGAAGT
>Nannospalax galili
AGCAGGCCTGTACCCC-AGGGACCAAATT-GGGTT-GAGCCATCTCCAATGTTTAGCATTAGGCAAT-GGTAAGAAACCT
AACTACAGAAAG-----------TCTGTATGC-AGTGACTCATCATGTCACATCACAGAACCCCACATCAGCCAGTGTGG
TTAGGAGCTGGGCTTCC-AGCACA--TCATAAAC----AGTACTGCCAGT-CGCTAAACTGATGAACTCAAGGGAGAAA-
------CCTCCAC----CCATATTCAATCACCCTATCTGGCATCTGGCCCAC----------------ATGGTTAGATGC
ATGGATAGAAGGATACATAGATACATGGATGA-GTGGGTAACATGTAGGCAAAGGGATTTG----TTTAAGAAAGGAA--
----
------------------------------------GTGGATTCTTAGCCTGAGATCTATCATGGCTCCTCCCACT----
----
---CCTTTAAAA

A---
--AAATTT
TTTT--
---AATGTCTATA-ATCACACAGGAAGCAG
AGGCAGGAAGATGA-G-ATGATCTTGG---G-CTTGGAAC----TGTTTAGCAAGAC--TG-CT--CAAAAAAAAAAC-A
AAAC-TGAAAA--CTGGATTCAATTCAATAAAGGTTTATCAGATTCCCAGAACACTGGTTTTCTGAGT----GGCC----
-----------------CACAATGAACACATCTTTGGAGATGGG-AATCAGAAGTTAACACATGTGGC-AAGCT--GCAC
A-------CT--AAAAGC
>Jaculus jaculus
ATCCTCTCTCTACCCA-AGGGATCAAATCAGGGTTAGGGCCGTCTCCGATGTTTGCTATTAAGCAAT-GATG--------
-AGTACAGAAAG-CCGACAGCTTCCCATCCGC-AGTGACTCATCATATCACATCACAGCACCCCACATCAGCCAGTGGGG
TCAGAAGCTAGGATCCCCTACTGATTTTGTAAAT----AGTACCCCCAGG-AGGTAAACTGATGAACTCAAGAGAGAAA-
------CACATGC----CCATATGTAATCATCGTATCCAGCGTGGAGCCCAT----------------------------
---------------CACGGATGGATGGATGG-ATGA------------CAAGCAAAAGAA----CTTAAGAAAGGCACT
GGCACTCGGGAGGCAGAAGTAGGAGGCTCGCCATGAGTTCAAGACCACCCTGAGATTACATAGTGAATTCCAGGTCAGCA
TAGACTAGAGGGAGACCCTACTTCAAAAACAAGGCAATGGAATATTA----GAGATCTTTCATGACTCTTCTA-------
----
---TCTTCAC--

----ATGCATGCATACCTATGTATGTATATCTATATACATATATAATCTATGTAACAGACACACTCACACATATATGTGC
ATATATATATGCACATATACAAATATATATATATATATGCACATATACGTATATATACCAGT--AACCCACAGGAAGAAG
AGGCAATAGGATG--G-AAATTCAAGGCCAG-CCCAGAA-----TCCATAGCAAGACCCTATCT--CAAAAAAAAAAAAA
AAAA-TG------TTGAATTCAAT-----AGGAGTTTAT--GATTGCTACAGCACTAATTTTCAGAGGAACCACCCTACC
CCTGGGGTGGCCTAGAGCATTACAGGCACAAATTTGGGAACAGA-ATTCAGAAGTGAACACA--TGGC-AAGCTCCCCAC
ATGAATATTG--AAAACT
>Castor fiber
TGCAGGCCTCTACCCA-AGGGACCAAACCAGTGGTAGAGCCCTCACCAATGTTTACCATTAGGCAAT-GGTAAGACATCG
AAGTTCAGAAAC-TCTACAGCCTCCCATATGC-ACTGACTGACCATATCCTATTAGAGCACACCACATTAGCCAGTGTGG
TCAGGAGCTAGGCTCCCAGGCAGACTTCATAAAT----AGTATTCCAAGTTGGCTAAACTGATGAACTCAAGGAAGAGAG
AA----CGCCCCAAATTCT-GACCCAATTCCCATAACTGGCACTTGGCCCATAGCAGT----------------------
-CATATAC----CTAGATGGATGAATGTATGG-ATGG------------CAAAAGAATTTA----TTTAAGAAGAGCA--
----
------------------------------------TTGGAGTCTTAGCCAGACATCTATGATGGCTCCTCCACCC----
----
---TCCTTTAAT
TTAAAAA---
--TGAAAA
ATTTAGCCAGGTGTAGC-AGA---GCACACTTGTA---
--ATTCC-----AACACCCAGGATGCTG
AGGCAGAAGAATG--GCAAGTCCAAGGCCAG-CCTGG-GCTACATAGATAGCAAGACCATGTCT--CAAAAAA--C----
--AC-TGGA----TTAAATTCTAT-----TGAGATTGACCAAATTTCTGAAACAATGGTTTTCAGAG-----ACCCT---
--TAGAGTGCCCCAGAGCATAATGCACACAACCTCAGAGCCAGG-AACCAGCAATTAACACA--TGTC-AAACTCACCAC
ATGAGTCTTC-TTACACT
>Dipodomys ordii
TGTAGGCGTCTACCCC-AGG-----AACCAGGGACAGAGTCCTCTCCAATGTTTACCATTAGGCAATGGGTAAGAAATCA
AATTACAGAAAG-TCTACAGCTCCCTATA-GC-ACTGACTCAAAATATCACATCAGAGCATACCACATTGGCCATTGTGG
TCAGCTGCTAGGCTCTCAGGCAGATTTCATAAAT----AGTACTCCCAGT-AGCTAAA---GTGAACTCAAGAAAAAGAG
ACA---CTTCCCAAACCCT-AAT-CAGTTACCATATCTAACA-TTAGCTCATACCAGT----------------------
-CA---------GAATATAGATGAACGAATGG-ATGG------------CAAAAAAAAAAA----TGT-------GTA--
----
------------------------------------T----GTCTTATCCTGAGATCAGTTGTGCCTCTTCCTCTC----
----
---ACCTTCAAT
TCAAATT---
--TG----

--AAAGCTATGTAAGAGA-------CTG
AAGCAGGATCATG--GCAACTTCAAAGGAAA-CCTGG-G----ATATTCAGCAAGACTGCCTCCCCCAAAAA---CTTAA
AAACTTGAA----TTAATTTCAAT-----AGAGACTTGTCAAATTCGTGGAGTGATGTTTTTCAGAG-----ATCTC---
--TAGGATACACTA-AGCAAAATGTACATAATCTCAGAGTCAAG-AATCTGAAATTAACATGTGTGGC-AAGCTTCACAC
ATGCGTCTTT-TGACACT
>Cavia porcellus
AGCAGGCCTCTACCCC-AGGGACTAAATCAGGGGTAGAATTGTCT-CAGTGTTTACCATTGGGCAGT-GGTAAGAAATCA
AAGTACAGAAAG-TCTCCAGCTTTC-AGATAC-AGTGACTCATCACATCACATCATACAA-----CATTAGCCAGTGTGG
TCAGGGGCTAGGCTCACAGGCAGACTTCATAAAT----ACTACTCCTGGT-AGCCAAACTGATGTACTTAAGGAAAGAAG
AAAGAGCTTCCTGCA--CT-GGTCCAATCC-----------ACCTGGCTCCTGGCAGC----------------------
---------TGTATGCGTAAATAGAGGGATGGAATGG------------CAAAGGGATTTA----TTGAAAAAGAGCAAT
AAA---
------------------------------------GTCGACTCCTAGCCAGGAATCTAGCGTGACTCCTCCATTC----
----
---TCTTCTAAT
TTAAAAG---
--TG-AAA
ATT---

-----TGAA----TTAAATGTAAC-------AGGCTTATCAAATTCCTGGAGCAGTAGTTTTTGGAA-----ACCCCTA-

--TAAGA-GCACCAGAGTACAAGGCACACAACTTCAGAGCTGAT-AAACAGAAATTAAC----GTGGC-AGGCTCCCCAC
A--AGTCTTCTTTACATT
>Heterocephalus glaber
AAAAGGCCTCTACCCC-AGGGACAAAATTAGGGGTAGAATCTTCTCCAGTGTTTACCATTAGGCAAT-GGTAGGAAATCA
AAGTACAGAAAG-TCTCCAGCTTCCCATGTAT-AGTGATTCATCACATCACATCACACCATACCACATTGGTCAGTATGG
TCAGGGGCTAGACTCCTTGAAAGACCCCACAAAT----AGTACCCCTGGT-AGCCAAACTGACTCAGAGCT---------
------CCCTGCAT---CT--AGCCAGTCACCTTATTCAGCACCTGGCTCATTGCAGT----------------------
---------TGGATGCATAGATA-ATAGATGGAATGA------------CAAAAGGATGTA----TTTAAAAAGAGCCA-
----
------------------------------------GTGGAGTCCTAGCCAAGAATCTTGCATGGCTCCTCC-ACTGGGG
ATTTAGCTCGGTTCCACCATCTGCCTCGAAAGAGCAAGGTCCTAAATTCATTCCCTGGT---------------------
---ACCAATAAA
ATAAAAA---
--TG-AAA
ATT---

-----TGAA----TTAAATGTAAC-----AGCAGTTTATCAAATTCCTGGAGCAGTAGTTTTTGGAA-----ACCCCTA-
----CAGTGCCCCAGAGCACAATGTACACAACTTCAGAGCTGGT-AAGCAGAAATTAACAC--GTGGC-AGGCTTCCCAC
AAGAGCCTTCTTTACGCT
>Ictidomys tridecemlineatus
AGCAGGCTTCTACCCC-AGGGACCAAATCAAGGGTAGAGCCTTCTCCAATGTTTCCCATTAGGCAAT-GGTAGGAAATC-
AAGTACAGAAAG-TGTACAGCTTCCCATATGC-AATGACTCATCATATCACATCACACCACACCACATCAGTTAGTATGG
TCAGGAGCTAGGCTCCCAA--AGGCTTCATAAAT----AGTACTCCCAGG-AGTTAAACTGATGAACATAAGGAAGAGAG
AA----CTCTCCAC---CTCCACCCAGACACCATATCCAGTACCTGCCCCAGTGCAAT----------------------
-TAAATGCATAGCTAGATGGATAGACAGAT-GAATGG------------CAAAGGAATTTA----TTTAAAAAGAGCA--
----
------------------------------------GTGGAGTCCTAGCCAGAGATCCATCATGG------CTCCA----
----
---TTAAC
TTAAAAA---
--TG-AAA
ATTT--

-----TGAA----TTAAATTCAAT-----GACAGTTTATCAAATTCCTGG--TGGTGTTTTTCAGAGAT---ACCTC---
-----GGTACCCCA-AGCATAATGCATGTAACTTTGGAGCTAGA-GA-CAAAAAGTAACACCTGTGGCAAAGCTTCCCAC
ATGAGTCTTCGTCACACT
>Marmota marmota
AGCAGGCTTCTACCCC-AGGGACCAAATCAGGGGTAGAGCCCTCTCCAATGTTTCCCATTAGGCAAT-GGTAGGAAATC-
AAGTACAGAAAG-TGTACAGCTTCCCATATGC-AATGACTCATCATATCACATCACACCACACCACATCAGTTAGTATGG
TCAGGAGCTAGGCTCCCCAA-AGGCTTCATAAAT----AGTACTCCCAGG-AGTTAAACTGATGAACATAAGGAAGAGAG
AA----CTCTCCAC---CTCCACCCAGACACCATATCCAGTACCTGCCCCAGTGCAAT----------------------
-TAAATGCATAGCTAGATGGATAGACAGAT-GAATGG------------CAAAGGAATTTA----TTTAAAAAGAGCA--
----
------------------------------------GTAGAGTCCTAGCCAGAGATCCATCATGG------CTCCA----
----
---TTAAC
TTAAAAAA--

-----TGAAAATTTTGAATTAAATTCAATGACAGGTTATCAAATTCCTGAAGTGGTGCTTTTCAGAGAT---CCCTC---
----GGGTACCCCA-AGCATAACGCATGTAACTTTGGAGCTAGA-GACCAAAAAGTAACACCTGTGGCAAAGCTCCCCAC
ATGAGTCTTCTTCACACT

Marker 25386

```
>B1F
--------------------------------------------------------------------------------
--------------------------------------------------------------------------------
--------------------------------------------------------------------------------
--------------------------------------------------------------------------------
--------------------------------------------------------------------------------
--------------GCCAGGCATAGTGGCA-CACAC-CTGTAATCCCAGCACTTAGGAGGCTGAGGCAGGAGGATCTGCT
GTGAGTTTGAAGCCAGCCTGGGCTACATAGTGAGTTC-------------------------------------------
--------------------------------------------------------------------------CAGGCC
AGCCTGGGCTACACAGTGAGACCC-TGTCTCAAAAAA-CAAAAAAA----------------------------------
--------------------------------------------------------------------------------
--------------------------------------------------------------------------------
--------------------------------------------------------------------------------
--------------------------------------------------------------------------------
--------------------------
>B1F1
--------------------------------------------------------------------------------
--------------------------------------------------------------------------------
--------------------------------------------------------------------------------
--------------------------------------------------------------------------------
--------------------------------------------------------------------------------
-------------AGCCGGGCGTGGTGGCA-CACGC-CTGTAATCCCAGCACTCGGGAGGCTGAGGCAGGAGGATC-GCC
GTGAGTTCGAGGCCAGCCTGGGCTACATAGTGAGTTC-------------------------------------------
--------------------------------------------------------------------------CAGGCC
AGCCAGGGCTACATAGTGAGACCC-TGTCTCAAAAAAACAAAA-------------------------------------
--------------------------------------------------------------------------------
--------------------------------------------------------------------------------
--------------------------------------------------------------------------------
--------------------------------------------------------------------------------
--------------------------
>Mus musculus
----------------TTAGTC---TGGGGGGAAAGCTAGGA--CT--AAGCA-----------------GAGTGCCAGG
ACCCCACCTAGTA--GGCACAC------------------------------------------------ACTCACACAC
TCACTCCACCCTTTTCAGTATCC---------------------------------------------------------
----------------------TAGGACCCATCTTTAAAGCACTTTGTA-TGTCTATCA-TTCAGGGGAA-TCTATTCTG
CCTTGACCCAAAGCTTTTTCTGACACT-GATCCCC-----TCCT-GCACAATC---------------------TTAAG-
-GAA-----------------------------------------------------AGGCTGAGACAGGAGGATC----
ACAAGTTTGAGACCAGCCTGTGCTATGTAGTTAAAAG-------------------------------------------
-----------------------------------------------------------------------TTCCAGGCC
AGCCTGGGCTACATAGG----CTT-TGTTTCAAAAAAA------------------------------------------
-----------------------------------------------------------------------------AAA
AAAAAAAAAAAAGTAGACTG-ACCAGCTTTGGCTT-GTCAGCC-TGACTA-----CCCAAAGAATACCAAGAGGGAAAGG
ACGTCCAAGTTGAAA---ATGGTTTTAATAGCATCCTGTGGGGAAAGG-----------------GT-AGGGTCACATCA
CTGACTC---AGACTTAACAGGATTCTTTGGTGTGAGCCTCATACTTGGGTATGTTCAGCTCTAAGACACAGATGTGCTG
C-------------------------
>Rattus norvegicus
----------------CTAGTC---TGAGGGAAAGGCTAGGA--CT--AAGCA----TGTTACCTCTGCAGGATGCTAGG
ACCCCACATAGTACGGGCACAC------------------------------------------------ATATACATAC
TTGCTCCACCCTTTTCCATATCG---------------------------------------------------------
----------------------TAGGACCCATCTTTAAAGCACTTTGTA-TGTCTATCA-TTCAGGAAAA-TCTATTCTG
CCTTGACCCAAAGCTTTTTCTGACACT-GATCTCC-----TCTT-GCAGAATC---------------------TAAAG-
-GAC-----------------------------------------------------AGGATAAGACAGGAGGATC----
ACAAGTTTGAGACCAGCCTGTGCTAGGTAGTTAAAG--------------------------------------------
-----------------------------------------------------------------------TT-------
--CCTGGTTTA----GGGAGACTT-TGTCTCAAGAAAA------------------------------------------
--------------------------------------------------------------------------------
-----AAAGTAACTAAACTG-ACCAGCCTTGGCTT-GTCAGCC-TGATTGGCCATCCCAAAGAATACTAAGAAGGAAAGG
ATGTCCAAGTTGAAA---ATGGCTTTAACAGCATCCTGTGGGGAAAGAT--CTTACCCCTACACTGC-AGGGTCACATCA
CTGACTC---AGATTTAACAAGATACTTTGGTTTGAGCCTCATACTTGGGTATGCTCAGCTCTAAGACAT-GCTGTCCCC
ACACACCAGCAA-GCCA-AAAGCTGT
>Apodemus sylvaticus
ACGAAGTGACAGGTTTCTAGTCTGA---GGGAAAGGCTAGA---CT--AAGCA--TT-----CCCCTGCAGAATTCCAGG
ACCCCACCTAATAGACGCACACACGCACGCGTGCACATGTACACACACACACACACAAACACACACACNNNNNNNNNN
TCATTCCATCCTTTACAAAATCC---------------------------------------------------------
----------------------TAGGACCCATCTTTAAAGCACTTTGTA-TGTCTATTA-TTCAGGAGAA-TCTATTCTG
CCTTGACCCAAAGCTTTTTCTGACACT-GATCCTC-----TCTT-ACAAAATC---------------------TGAAG-
-GAA-----------------------------------------------------AGGCTGAGACAGGAGGATC----
ACAAGTTTGAGACCAGCCTGTGCTATGTAGTTAAAG--------------------------------------------
-----------------------------------------------------------------------TTCCAGGCC
AGCTACA----------TAGACTT-TGTCTCCAAAAAA------------------------------------------
--------------------------------------------------------------------------------
------AAAAAAGGTAACTA-GACTGACCAGCTTTGGCTTGTC-AGCCTGACTAGCCCAA-GAATACCAAGAGGGAAAGG
ATATTTAAGTTGAAA---ATGGTTTTAATAGCATCCTGTGGGGAAAGATAGCTCACCCCTACATTGT-AGGGTCACATCA
CAG------------------AATTCTTTGGTTTGAGCCTCATTCTTGGGTATGCTCAGCTCTAAGACAT-GCTGTCCCC
ACAAACCATAAA-GCTA-AAAGCTGT
```

```
>Cricetulus griseus
AGGAAATGACTATTTTCTAGCCTGATGAGGGAGAACCTAGAA--CT--AAGCA--TTTGTTA-CCCTGCAGGATGCCAGG
ACCCCACCTA-GGCAGACTCCT-----------------------------------------CCCCCCCCCCCACACAC
ACACACTACCCCTTTAAATGCCC---------------------------------------------------------
----------------------TAGAACCCATCTTTAAAGCACTTTGTA-TGTCTGTCA-TTCAGGAG---TC-------
--------------------------------------------------------------------------------
----------------------------TGGCACATGCTATAATGCCAGCACTCA-CAGGCTGAGGCAGGAGGATC----
ATACGTTTGAGACCAGTCTGTGCTATGTAGTTAAAA--------------------------------------------
-----------------------------------------------------------------------TTCTAGGCT
AGCCTGG----------AAGACAC-TGTCTCAAGAAAA------------------------------------------
--------------------------------------------------------------------------------
----------AAGCAAACTG-ACTAGCTTTGGCTTTGTCAACC-TGACTGGGCAGCCCAAAGAACACCAGGAGGGGAAGG
ATACCCAAGTAGAG-CCCATGATTTTAATAGCACCCTATGGGAAAAGATATCTCACCCCC----TGT-AGGGTCATATCA
CTGACCC---AGACCTAACACAATAGTTTGGTTTGGGTCTCATACTTGGGTATGCTCAGCTCTAAGACAT-GCTGTCCCC
ACAGACCATAAA-GCCA-GAGGCTGT
>Mesocricetus auratus
ACACAATGACTAGTTTCTAGTCTGATGAGGGAGAACCTAGTA--CTAAAAACA--TTTG---------------------
--------------------------------------------------------------------------------
---CTCTACCCCTTTAAATGCCC---------------------------------------------------------
----------------------TAGGACCCATCTTTAAAGCACTTTGT-----CTGTCA-TTCAGGAG---TC-------
--------------------------------------------------------------------------------
----------------------------TGACATGTGCTATAATGCCAGCACTCA-GAGGCTGAAGCAGGAGGATC----
ATAAGTTTGAGACCAGTCTGTGCTATGTAGTTAAAAG-------------------------------------------
-----------------------------------------------------------------------TTCTAGGCT
AGCCTGGG--------AGAGACTCTTGTCTCAAGAAAA------------------------------------------
--------------------------------------------------------------------------------
----------AAGCAAACTG-ACTAGCTTTGGCTTTGTCAACC-TGACTGGGCAGCCCAAAGAACACCAGGAGGGGAAGG
ATACCCAAGCAGAG-CCTGTGATTTTAATAGCGCCCTATGGGAAAAGATACCTCACCCC-----TGT-AGGGTTATATCA
CTGACTC---AGACCTAACACAGTTGTTTCATTTGGATCTTATACTTGGGTATGCTCAGCTGT-----------------
---CACCATAAA-GCCA-GAGGCTGT
>Peromyscus maniculatus
------------GTTCCTAGTCTGATGAGAGACAACCTAGGA--CT--AAGCA--TTTGTCA-CCCTGCGGGATGCCAGG
CCCCCACCTA-GACAGGTCCCTG--------------------------------------CCCACCCACCCA-GCCCCC
ACACTCCACCCTTTTCAATGCCC---------------------------------------------------------
----------------------TAGGACTCATCTTTAAAGCACTTTGTA-TATCTGTCA-TTCGGGAG---CCTATTCTG
CCTTGACCCAAAGCTTTTTCTGACACT-AATCCCC-----TCCT-GCAGAATC---------TGAGGGA-GGGATAAA--
-GAAGCAGTTAGTAGCCAGGTGTG---CTAGCATATGCTATAATGCCAGCACTCA-AAGGCTGAGGCAGGAGGATC----
ATAAGTTTGAGACCAGTCTGTGCTATGTAGTTAAAG--------------------------------------------
-----------------------------------------------------------------------TTCCAGGCT
AGCCTGGGCTACGTAGAGAGACTT-TGTCTCAAGAAAA------------------------------------------
--------------------------------------------------------------------------------
----------AAGCAAACTG-ACTAGCTTTGGCTTTGTCAATG-TGACTGGCCAGCCCAAAGAACACCCAACAGGGAAGG
ATGCCCAAGATGAGACC-GTGGTTTTAATATCTCCCTATGGGAAAAGATACCTCACCCCC----TGT-AGGGCCAAATCA
CTGACTC---AGACCTAACACGATCGTTTGCTTTGGGTCTCATACTTGGGTATGCTCAGCTCTAAGACAT-GTTCTCCCC
ACAGTCCATAAA--TCA-AAAGCTGT
>Microtus agrestis
AGGAAATGACTAGTTTCTAGTCTGGTGAGGGAGAACCTAGGA--CT--AAGCA--TTTGTTACCCCTGCAGGATGCCAGG
ACCCCACCTA-GGCGGACACAT--------------------------------------------------GCACAC-T
ACACTACAC-TTTTTCAAGGCCCTAGGACCCATCCTTTTTGTTGTTGTTGTTATTGNNNNNNNNNCTGGGATTAAAGGTG
TGCACCACCACCGCCCAGCTTCTAGGACCCATCTTTAAAGCACTTTGTA-TATC-GTCA-TTCAGGAG---TATATTCTG
CCTTGACCCAGAGCTTTTTCTGACACTAAATCCCC-----TCTT-GCAGACTC---------TGAGGGAAGAGATAAA--
-GAAGCAGTTAATAGCCAGGTGTG---CTGGCGGATGCTATGATGTCAGCACTCA-GAGGCTGAGGCAGGAGGATC----
ATAAGTTGGAGAGCAGTCTGTGCAATGTAGTTAAAG--------------------------------------------
-----------------------------------------------------------------------TTCCAGGCT
AGCCTGGGCTACAAAGGAAGACTC-AGTCTCAGGAAAA------------------------------------------
--------------------------------------------------------------------------------
----------AAGTAAACTG-ACTAGCTCTGGCTTTGTCAACC-TGACTGGCCAGCCCAAAGAATACCAGGAGGGGAAAG
ATGCCCAAGTTGAGG-CTGTGGTTTTAATAGCACCCTATGGGAAAAGATACCTCATCCCC----TGT-GGGGTCACATCA
CTGACTC---AGA-CTAACACAATTGTTTGGTTTGGGTCTCATACTTGCATATGCTCAGCTCTAAGACAT-GCTGTACCC
ATAGACCATAAA-GCCA-AAAGCTAT
>Nannospalax galili
--------------TTTGAGTCTGA---GAGA----CTAGGA--GT--AAGCA--TTTGTTATTCCAGCAGGGTGCCAGG
ACCCTGCCTA-TGGA-ACCCCT----------------------------------------------ACCCTCACCTCA
CTCCCTAGCTCTTCTCAATGCTTT--------------------------------------------------------
----------------------TAGGATCTGTCTTTAAAGCACTCTGTACTGTTTGCCA-TTTAGGAGA--CTCATTCTG
CCTTGACCCAGGGCTTTTTCTAACTCT-AATCCCCTCTTTTCCT-GCAGAATC---------TAAGGAAAGGGATAAA--
-GAAGCAA-TGATAACCAGGTGTG--GGTAGCACATGCTG---TGCCAGAACTCA-AATGCTGAGGCAGAAGGATC----
ACAGGTTTGAGACTAGCCTATGCTACATAGTAAA----------------------------------------------
-----------------------------------------------------------------------TTCCAGGCC
ACCCTGGGCTACATAGCGAGACCC-TGTCTTTAAAAAA------------------------------------------
--------------------------------------------------------------------------------
-----ATGAAACTC-AACTG-ACATGCTTTGGCTTTGCCTAGC-TGGCTGATCAACCCAAAGAATACCAAGG---GAAAG
ATGCCCAAGTAAGGACCTGTGGTTTTAATAGCATCCCATCGGAAAAGGCGTTTCACCCCCGCA-TCATGGAGTCAC-TCA
CTGATTCCTTAGATCTAAGAGGATTACTTGTTTCGAGTCTCATCTTTGGGTGCTTTCAGCTCTAAGACGT-GATGTCCTC
ACAGACTATAAA-ACCA-AAAGCTGT
>Jaculus jaculus
------------GTAGTGACTATAAT--GG---------GGATTCT--AAGCA--TTTGTTATCCATGCAGGGTTCCAAA
ACCCTGCATAAAGCAGACCCCG----------------------------------------------------------
```

----CTGGCCCTTCTCAGTGCCTTT---
----------------------AAGGGTCTGT---TAATGCACTTTGTACTCTTACTCAGTTCAGGAGATTTGTATTCTG
CCTGGACCCA-CCATTTTTCTGATCCT-AACCTTTACTTTT-CT-GTAGAATC---TGAGTTGGGAAGGAGGGTTAAAG-
-GAAG----TAATAGCCAGGTGTG---GTGGCACATTCTGTAATGTCAGCAGTTGGGACACTGAGGCAAAAGGATC----
ACAAGTTCAAGGCCACCCTGTGTTATGTAATGAGTTCTTTAAAAAAATTTGTTTTAGCCTGGTGTGATGGTGCCCACCTT
TAATCCCAGCACTCAAGAGGCCAAGAGAGGATAGCTGGGGGTTCCAGGCCAGCCTGACACTACATAGTGAATTCCAGGTC
AGCCCGGGTTACAGCAAGTGAAGC-CCTCCCTGGAAAA--
---ATAAACAAACAACAACT
AA---ATCT-AAGAACCCTAAACTAGCTCT-GCTTTACTGTTC-TGGCTGGCCAGTCCAAAAAGCAGCA---GAGGA---
-----------GGGCCCTGTAATTTTAATAGCACCCTGTAACCAAAGACA--TCCCCCC---A-TCACAGGGTCACATCA
TTGACTGCTTGGGTTTAACAGGACTGTTC----TGGATTTTGTTCTGTGATGCTCTTAGCTATAAGATAT-GCTATGCTC
ACATACTGTAAA-AGCAGGAAGTCAT
>Castor fiber
AGGGAGCAGC--GT-TC-AG-CAGTT--GGGA-AAGTTAGGA--AC--ACAAG---CTCCCATCCCTGCAGGGTGACAGG
ACCCTACCTAAGGCAGACATCA--
-----AGACCTTTCTTGATGCTTT--
----------------------TGGGTTCTATCTTTAAAGCATTTTG-ACTGTTA-----CTCAGCAGATTTGTGTTCTG
CCCCAATATGTGTCTTTT-CTGACCCT-AG-CCCC--TTTTCCT-GCAGAATC----AAGCTAAGGGGGGAGGAT-AAGA
GGAAACAATTAATAGCCAGGCATGATAGTG-CATGC-CTCTAATTCCAGCACTTGGGAGACTGAGGCAGAAG-AT-----
ATGAGTTTGAGATCATCCTAGGCTACCTAGT---

-----------------GAAATCC-TGTCTTGAAAAAAA---
---GAGAGGAAGCAATAATT
AA--AAAAAATAAATAACTG-ATCAGCTTTGGCTT---------TGGCCAGCCAACCCAAAGAGCACCAAGAAAGGAAGG
ATGCCTACACTGGGAACTGTAGTCTTAGTAACAGCCCATGAGAAAAGACATCTCACCC-CACA-TGTTAGGGCCACGTCA
CTGATTCCTTGGTTCTAACAGGATTGTTCTTTTTAATTCTCATCCTTGGGTACCCTCAACTCCAAGATAG-GCTGTCCTC
ACATACTCTAAG-GCCA-GAAGCCAT
>Dipodomys ordii
AGGAAATAATTACTTTC-GGTCTTATGAGTGA-------GGA--CT--AGGCATTTTTGTTGCCTCCACAGCTTGCCAGC
ACCCTGCTTAAAGCAGACCCCT--
-CAGCTAACTCTTCTTGATGCCTT--
----------------------TGGGATCTATCCTTAAAGCACTGTGTACTTTTA-----TTCAGGAGATTTGTATTGTG
TCCCAATCCA--TCTTT--CTCACCTT-AATCCCC---TTTCCT-GTAGAAATAGAAACACTTGAGGGAGGGGATAAAGA
TGAATCAATTTTTAGCTGGAGGTGGTAGTA-AATAC-CTATAATCCCAGTACTTGTGAGGCTAAGGTAAGAGAATC----
CCAAATTTGAGGCCAATTTGGGCTGTTCAGC---

-----------------AAGACTC-T---CCAAAAAAAA---
---AAAAAAAAGCAATAAAA
AAGAAAAAAATAGAA-CCTG-AACAGCTTTAGCTTTGCTGGTCTTGGCCAGCCAGCCCAAAGGGCACCAAGGAAGG----
------AAGTTGGGACCTGAGGTCTTCACAGTATCTTGTG-GAAA-GAC--TCCACCC-AACA-TCTTAGAGCCACGTCA
CTCGCTCCTTACATCTAACAGG---GTTCTTTTTGACTCTTGCTCTTTGGTGCCCTCAGTCCCAAGACAC-AATGCCCTT
ATATACTATAAA-GGCA-AAAGCTAT
>Cavia porcellus
AGGAAATTAACAGTTTTAAATCTAATAAA---------GGGC--CT--AGACATTTCTGCTACCCCT-CAGAGTGCAAGG
GCCCTGGAGAAGCCAGAGCCTT--
-----CAGGCCGTCTTGATACCTT--
----------------------TGGGATCTGTCTTTGAAGCACTTTGTACTGATA-----TTCAGGAGGTTTGTCATTTA
CCCTGACCGATGTCTTT--ATGATCCT-AATCTCC------TCTAGCAGAATT-----AGTCTGAGGGAGGGGATAAAGA
GGAAG---

------------------------------CAATAAAAAAAATCCTCGGGGCTGGGGATTTAGCTCAGCAACGTAAGCAC
CTGCCTTGCAAGCAGGAGGTCATGAGTTTGATACCTGGTACCGAGAAAAAGAAAAAAAAAATTAAAAAAAGAGTTAAAAA
AAAAAAAATCCTCAAAACCA-GGCAGTT-GAGCTTTGCCAGCC-TGGCCAGCCA------AGAGCATCAAGGAAGGAAGA
ATGTAAAAGTTGGGACCTGTGGTGTGAACAGCATTCTGTG--AGGAGACATCTCACTCCCATA-GCTTGCAG--------
----------GGAC-CAACAGGATTGCA---------------CCTTTGGATTCTGGTCCTCCAGGAT---GCTGTCCTC
TCAGGCCATAAT-GCCA-GAGGCTGG
>Heterocephalus glaber
AGGGAATTAATAGTTTTAAATCTAATAAA---------GGGC--CT--GGACATCTCTGCTATCCCTGCAGAGTGCCAGG
GCCCTGTGTAAGCCAGACCCTT--
-----CGGCCCTTCTTGATGCCTT--
----------------------TGGGATCTGTCTTTAAAGCACTTTGTACTGTTA-----TTCAGGAAGTCTGTAATTTG
CCCTGACCAATGTCTTTTTCTGATCCT-AATCCCC-----TCCT-GAAGAATC-----AATCTGAAGGAGGGGATAAAGA
GGAAG---

------------------------------CAATAAAA--

-----AAACCCTCAAAACCA-GCCAG-TTTGGCTTTGCCAGCC-TGGCCAGCCAGCCCAGAGAGCATCAAGAAAGGAAGG
AGGCAAAAGCTGGGACCTGTGGTGTTAATAGCATCCTGTGAGAAAAGACATCTCACCCCCATA-GCTTAGAGGGACTAGC
AGGATT----------------------------------TTTCTTTTTAATTC-TAGTCCTTGAGGGCATGCTGTTCTC
ACATACCATAAA-GCCA-GAGGCTGG
>Ictidomys tridecemlineatus
AGGAAATGAATAGTTTT--ATATAATGA----------GGGA--CT--AGGCA--TTTGTTACCCCTGCAGGGTGCCAGA
ATCCTGCATAAGGTAGACCCCT--
-----CAGCCCTTCTTGATGCTTT--
----------------------TGGGATCTGTCTTTAATGCACTTTGTACTGTTA-----TTCAGGAGAT---TAGCCTG
CCCTGACCCACACCTTTTCCTGACCCT-AATCCCC--TTTTCCT-GCAGAATC-----ATTCTGAGGGAGAGGATAAAGA

GAAAG---

------------------------------CAATAAAA--

-----------ACAAAACTG-ACCAGTTTTGGCTTTATCAGCC-TGGCCAGGCAGCCC--AGGGCACCAAGGAAGACAGG
CTATGTAAGACAAGACCCATGGTCT-CACAGCATGCTGCCTAAAGAGACATCTCACCCCCACA-TCCTGGGGTCATGTCA
CTGACTCCTGGGATGTAATAGTGCTATTCTCTTTGATTCTTATCCTTGAGTGCCCTTAGTTCCAAGGCAC-TTTGACTCC
ACATACTGTAAAAGCTA-ACA-CTGT
>Marmota marmota
AGGAAATGAATAGTTTT--ATATAATGA----------GGGA--CT--AGGCA--TTTGTTACCCCTGCAGGGTGCCAGA
ATCCTGCATAAGGTAGACCCCT--
-----CAGCCCTTCTTGATGCTTT--
----------------------TGGGATCTGTCTTTATTGCACTTTGTACTGTTA-----TTCAGGAGAT---TAGCCTG
CCCTGACCCACACCTTTTCCTGACTCT-AATCCCC--TTTTCCT-GCAGAATC-----ATTCTGAGGGAGAGGATAAAGA
GAAAG---

------------------------------CAATAAAA--

-----------ACAAAACTG-ACCAGTTTTGGCTCTATCAGCC-TGGCCAGGCAGCCC--AGTGCACCAAGGAAGACAGG
G-----------AGACCTGTGGTCT-CACAGCATGCTGCCTAAAGAGACATCTCACCCCCACA-TCCTGGGGTCATGTCA
CTGACTCCTGGGATGTAATAGTGCTGTTCTCTTTGATTCTTATCCTTGAGTGCCCTTAGTTCCAAGGCAC-ATTGACTCC
ACATACTGTAAAGGCTA-ACA-CTGT

Marker 7855

>PB1D10

---TTT-TGTTT
TTTTGAGACAGGGTCTTGCTATGTAGCCCAGGCTGGCCTTGAACTCACGATCCTCCTGCCTCAGCCTCCCGAGTGCTGGG
ATTACAGGCGTGCGCCACC-------ACG-CCCGGCT---

>Mus musculus
AGGA-TGT--CTAA-GACTCCCG-AAAACAGGGATACCCT-TGGGAAATGATGCTGGAAGAGCCGA-ATAATTGA--TCA
TGTTTTAAAGAGTGGTGATATAAAGAAGGAGAGAGGAAGATAAATGC---TAGAAAAG------GATTTTTTCT------
----------------------------ACCCTTAAACTCTTCAT----------------TTCTT-TCTTGTC-TGTTT
T---GAGACAGAATTGTGATAAGAAGCCCAGGTTGGCCTGAAGCTCATTATTGTACAGCTTCAACCTCGCAAGTGCTACA
GCTAAAG-TATGTACTACT-------ATACCCCAGCT---
--TGAACATCTTGTGGTATTTTGT----TTTTTTCC-TTG
GTTACCAAGAGGCTGG-TAACCCACT---GCCTCTGAAGTGGGCTATCCCATTCCATAGGCATTTGCGTCTCTCAAAAAG
AAGAAA------TATTTTTTTTCACCCCC-----------AAGAGGTTCCAGATCAGTTTGGTGAGGATTTCTCTCATGC
ACAGTAGCCAGTTTCTTAGCACTAAGAATTTAAGCAATTAGATCATTTTTC-TCTAAAAGATTTTATGATAGCCA--GAA
TAGGTT-CTCTTTCACACTATAAGCTCTACTCAAGACTTGGCATCTAATTGAAGCATAACTTTGATGGCAATCCTTTAGC
CAGGATTGGTTA-TAGCCCTGCTG
>Rattus norvegicus
GAGA-TGT--ATAA-GACTCCTG-GAAACAGGGATCTCCT-TGGGAAATGATGCTGAAAGAGCTGA-ATAATTGA--TCA
TGTTTTAAAGACCAGTGATACAAA--------GAAGAAGATAAAAGA---A--AAAGA------GATTTTT-CT------
----------------------------ACCCTTGAACTCTCCAT---TTC---------CTTCTT-GTTTGTTTTGTTT
TT--GACACAGAATTGCGTTATGTAGCCCAGGTTGGCCTGAAACTCACAATTGTACAGCTCCATCCTCC-----------
----------TGCACTGCT-------ATA-CCCAGCT---
--TGAACATCCCATGGTGTTTTGTTTTGTTTTTTCC-TTG
CTTACCAAGAGGCTGG-TAACCCACT---GCCTCTGAAGTGGGCTATCCCATTCCGTAGGCATTTGGATCTCTCAAAAAG
AGAAA-------TATATA-TTCCCCCACC-----------TGTAGGTTCCAGATTAGTTTGATGAGGATTTCTCTGCTGT
AGAGTAGACAGTTTCTGAGCACCAAGAATTTAAACAATTAGATCATTTTTC-TCCAAAAGATTCTATGATAGCCC--GAG
TAGGTT-CCCTTTCACACTATAAGCTCTACTGAAGCCTTGGCATCTGATTGAAGCATAACTTTGATGGCAATCCCTTAGT
CAGGATTGGTTA-TAGCCCTGCCG
>Apodemus sylvaticus
AGGA-TGT--CTAA-GGCTCCTG-GAAACAGGGATCTCCT-TGGGAAATAATGCTGAAAGAGCTGA-ATAATTGA--TCA
TGTTTTAAAGAGTGTTGACATAAAGGAG---AGAGGAAGATAAAAGT---TAGAAAAA------GATTTTT-CT------
----------------------------ACCCTTGAACTCTTCGT---TTC---------TTTCTC-GTCTGTTTTATTT
TT--GAGACAGAATTGTGTTATATAGCCCAGGTTGGTCTGAAACTCATAACCGTGCAGCATCAACCTCCCAAGTGCTACA
GCTAAAG-TACATACTACT-------ATACCCCACTTGAACATTTTTTATTTTATTATTTTTTAAAGATTTACTTTATA
TATATATATAGATAGATAGATANNNNNNNNNNNNCTCCCCCTTGAACATCTTGTGGTGTTT------TGTTTTTCC-TTG
GTTACCAAGAGGCTGG-TAACCCACT---GCCTCTGAAGTGGGCTATCTCATTCCATAGGCATTTGCATCTCTCAAAAAG
A-GAAA------TATATGTTTTTACCACCCCGCCCCTGCCCAGAGGTTCCAGATCAGTTTGGTGAGGATTTCTCTCATGT
GCAATAGCCTGTTTCTTAGCACTAAGAATTTAAACAATTAGATCGTTTTTC-TCTAAAAGATTTTATGATAGCCA--GAA
TAGGTT-CTCTTTCACACTATAAATACTACGCAAGCCTTGGCATCTGATTGGAGCATAACTCTGATGGCAATCCTTTAGC
CAGGATTGGCTG-TAGCCCGGCAG
>Cricetulus griseus
AGGA------CCTC-AA---CTC-AAAGCAGGGGTCTCCT-TGGGAAATGATGCTAGAAGAGATGA-GTAGTTGA--TCA
GGTTTAAAAAGTGGTGAGAAGGAAGGAG---AGAAGAAGATAAAAGT---CAGAAAAA------GATTTTT-CT------
----------------------------AACCTTGAACTCTTCAT--TGTT------GTTGTTGT-TGTC-TATTTGTTT
TT--GAGACAGAATCGTGCTATGTAGTCCAGGTTGGCCCAGAACTCATGATTCTACAGCCTCAACCT--GAAGTGCTGGA
GCTACAT-TATGCACTACT-------ATA-CCCAGCT---
--TGAACATCTTG-TGGTGTCTTGTCTTTTGTTTCC-TTG
GTTGCCAAGAGACTGG-CCACCCATT---GACTCTGAAGTGGGCTATCCCATTCCACAGGCATTTGCATCTCTCAAAGAC
AAAAGAGAAA--TATATGCTTTTTTTC--------------AAAGGTTCCAGATCAGTTTGGTGAGGATTTCTCTCATTC
ACAATAGCCAGTTTCTTAGCACTAAGACTTTAAACAATTAGACCATTTTTCCTCTAAAAGATTTTGTGATAACAA--GAC
TAGGTT-CCCTTTCACGCTATAAGCTCTACTCAAGTCTTGGCATCTGATTGAAGCATGACTTCGATGGCATTCCTTTAGC
GAGGTTTGGTTA-TGGCCCTGCCG
>Mesocricetus auratus
AGGA------CCTC-AACTTCCG-GAAGCAGGGGTCTCCT-TGGGAAATGATGCTGGAAGACATGA-GTAGTTGA--TCA
GGTTTAAAGAGTGGTGAGAAGGAAGGAG---TAAGGAAGATAAAAGT---CAGAAAAA------GATTTTT-CT------
----------------------------AAC---GAACTCCTCATTTTGTTGTTGCTGTTGTTGTCTATTTGTTTTGTTT
TT--GAAACAGAATTGGGCTATGTAGTCCAGGTTGGCCTAGAACTCATGATTCTACAGCCTCAACCTCCAGAGTACTGGA
GCTACAT-TATGCACTACCCACCACCATA-CCCAGCT---
--TGAACATCTTGTGGTGTTTTGG--TTTTTCTTCCCTTG
CTTGCCAAGAGACTGG-TCACCCACG---GACTCTGAAGTGGGCTATCCCATTCCACAGGCGTTTGCATCTCTCAAAGAC
AAAAGAAAAA--TATGCTTGTTTT-----------------AGAAGTTCCAGATCAGTTTGGTGAGGATTTCTCTCATTC
ACAATAGCCAGTTTCTTAGCACTAAGAATTTAAACAATCAGATCAGTTTTCCGCTAAAAGATTTTGTGATAGCAA--GAA
TAGGTT-CCCTTTCACGCTATAAGCTCTACTCGAATCTTGGCATCTGATTGAAGCATGACTTCGAAGGCATTCCTTTAGC
GAGGATTGGTTA-TGGCCCTGCCG
>Peromyscus maniculatus
AGGAC-----CTT--GAC-CCTG-G-AAATGGG-TTTCCT-TGGGAAATGATGCTGGAAGAGATGA-GTAGTTGA--TCA
GGTTTAAAGAGTGGTGA----TAAGGAC---AGAGGAAGATAAAAGT---CAGAAAAG----GAGAGTTTT-CT------
----------------------------AACCTTGTACTACTCATTTTTT----------GTTGTCTATTTGTTTTATTT

TT--GAGACAGAATTGTGCTATGTAGCCCAGGTTGGCCTAGAACTCATGATTCTACAGGCTCACCCTCCCAAGTGCTGGG
GCTACAG-TATGCACTACC-------GTA-TCCAACT---
--TGAACATCTTGTGTTGTGTTTT-GTTTTGTTTCCCTTG
GTTACCAAGAGACTGGGTAACCCACT---GACTCTGAAGTGGGCTATCTTATTCCATAGGCTTTTGCATCTTTCAAAGAC
AAAAGAGAAATGTATGTTTCCCCCCCCCCCC----------AGAGGTTCCAGATCAGTTTGGTGAGGGTTTCTCTCATTC
ACAATAGC------------ACTAAGAATTTAAACAATTAGATCATTTTTCCTCTAAAAGATTTTATGATAGCAA--GAA
TAGGTT-CCCTTTCACACTATAAGCTCCACTCAAGCCTTGGCATCTGATTGAAGCATGACTTTGATGGCATTCCTTTAGC
GAGGATTGGTTA-TGGCCCTGCCG
>Microtus agrestis
AGAA------CCTCAGACTCCTG-G--AAACGG-TCTCCTGGGG-------------------------AGT-GATACCA
GT-TTAAAGAGTGGTGA----TAAGGAA---AGGGGAAGATGAAGGT---CAGAAAAA----GACTTTTTT-CT------
----------------------------AACCTTAAACT-CTTATGTCTCTCTCTCTCTCTCTCTCTCTCTCTCTCNNNN
NN--NAGACAGAATTGTGCTA-CTAGCCCAGGTTGACCTAGAACTCATGATTCTACAGTCTCATCCTCCCAAGAGCTGGA
GCTTCAG-TATGCAGTACC-------ATA-CCCAGTT---
--TGAACATCTTGTGCTGTTTTGT----TTTGTTCCCTTG
GTTACCAAGAGACTGG-TAACCCACT---GCCTCTGAAGTAGACTTTCCCGTTCTGTAGGCATTTGCATCTCTCAAAGAC
AAAGGAGACA--TATATGGTTTATTTTTTC-----------AGAGGTTCCATATCCGTTTGGTGAGGATTTCTCTCATTC
ACAATACCCAGTTTCTGAGCACGAAGAATTTAAACATTTAGATCATTTTTCCTCTAAAAGATTTTATGATAGCAC--GAA
TAGGTT-CCCTTTCACACTATAAGCTCTGCTCGGGCCTTGGCATCTGATTGAAGCATGGCTTCAGTGGCATTCCTTTAGT
GAGGACTGGTTA-TGGCCCTGCTG
>Nannospalax galili
------------------------GATCA-GAT-TTTAAAGCTGAAAACTGGAGAAAATATCAT----------------
------------------GTTATCAGTC---AGAAAAAGGGGAAATTACCTAAGCTGA-ACA-TCTTTTTTTTTTAGAGA
AAAATTAAACAAAAACCAATGATCAAAGAT-GTTTAACT-CTTTTTT-GGGGTTT---TTTTTTTTTTTTTTTTT-TTA-
----AAGACAGGGTTGTGCTATGTAGCTCAGGCTAGCCTGGAGC-TAGCAT--TACAGCATTAGCTTCCTGAGTACAGGG
-TTACAG-TATGCACCACC-------ATG-CCCATCT---
--TGAACATCTTATTTTTT-------------CCCTGTTG
GTGAACAAGAGACTGG-CAACCCACT----ACTCTGAAATGGCCTATCCCATTCCATAGGCATTTGCATCTCTCAAAGAA
GAGAAAGAAAATAAATTT-TTTC------------------AAAGGTTCCAGATCATTTTGGTGAGGATTTCTCTCATTC
ATAATAGCCATTTTCTTAGCACTAAGAATTTAAACAATTAGATCAGTTTTCCTCTAAAAGATTTTATGATAACAA-GGAC
TGGGTT-CCCTTTCACACTATAAACT--ACTTGAGCCTTGGCATCTGATTTAAGCATGACTTTGATGGCATTCCTTTAGC
CAGTATTGGCTA-TGGCCCTGCAG
>Jaculus jaculus
AGA-------ATTAAAACACTTGGAAACATGAA-TTTCCTGTGTGGAATGATACAAAAAGGAATGA-ATACTTGAAAT-A
GTTTTTAGGGCTTAAAAAGAAATAATAT---GATAAAAGTCAGAATTTCTCAACAACA-TCAAACAA-TTT-CTG-----
----------------------------AC-CTTGAACA--TCATTCATCATTC---TTTTTTTCTTGCCTCTTTTGTAG
TTT-GAGGCAGGATTTCACCATGTAGCCCAGGCTGGCCTGGAAC------TCATGCAACCTCAACCTCCCAAGTGCTTTG
ATTACAGG--TGTGCA---------------CCAGCT---
--TGGAGATCTTG-GGTTT---------TTATTCCCTTTA
GTTAATAGGAAACTTG-TCACCCACT---GGCTCTCATGTGGCCTGCCCCCTTCCCTAGGCATTTGCATCTCTCACAGAG
GAGAGAGAAAATGATTTT-TTTC------------------AAAGGTTCCAGATCAGTTTGGGGATGATTTCTC-CATTC
ATAGTAGCCATTTTCTTAGCACTAGGAATTTAAACAATTAGATAGTTTTTCTTCTAAAAGATTTTATGATAACAA-GGAG
TGGGTTCCCCTTTCACAGTACAAACTCTAGTCCAGCTTTGGCATCTGATTTAAGCATCACTTTGATGGTGTTCCTTTAGC
TAGGATTGGCTG-TGTCCTTGCAA
>Castor fiber
AGAA------ATTAAGATCTTTGGAGAAAAGGA-TTTCCTATGGAAAATGATGCAAGAAGGGGTGGAATAGATGA--TCA
GTTTTTAATGCTGAAAG--------------GAGAAAAGTCAGAATTTCTCAAGTAAA-TGAGAAATTTTT-CT------
----------------------------ATCCT-GAACATCTTTTTTTGTTG----------TTGTTGGCTGTTT-GCGT
TTC-ATGTCAGGTTCTTGCTATGTAGCACAAGCTAGCCTTGAACTCCTGATCCTCCTACCTTAGCCTGCCAAGTACTGGG
ATGACAGGAATGTGCTGCC-------ACA-CCCAGCT---
--TGGACATCTTGGTTTTT---------TTTTTTTCTTTA
TTTAACATTACTCTGG-TGACCCACT---GGCTCTGGAGTGGCCTATCCCATTCCATAAACATTTGCGTCTCTC-AAGAG
GAGAGAAAA----ATTTT---TC------------------AAAGGTCCCAGATCAGTTTGGTGATGGTCTCTCTCATTC
ATCATAGCCATTTTCTTAGCACTAAGAATCTGAACAATTAGGTCATTTTTCTTCCATAAGATTTTGTGATAAAAA-GGAA
TGGGTC-CCCTTTCACACTACAAATTCTACTCGCATCTTGGCATCTGATTTAAGC---------ATGACATTCCTTTAGC
CAGAATTGGATA-CAGTCCTGCAA
>Dipodomys ordii
AGAA---------AAAAC-CTTAAAGAAATGGA-TTTTCTGTGGTAACTTATGCAAGAAAGGATG---------A--TCA
GATTTTACAGCTAAAAAGAAGAGAAAAT---GAAAAAAATCAGAATTTCTACAGAAAAATG-GAAAGATTT-CA------
----------------------------ACCCA-GAACATTTTTT---GATG---------TTTCT-AATTG----GTT-
-TCTGAGTCAGGTTCTCACTGGGTA-CCCATGCTGACTTTGAG---TGTATCATCTTACTTCAGCTGTGCAAGAACTGAG
ATTACAGGCATGTGCCACT-------ACA-CTTAGCT---
--TGATCATCTTTTTTTT----------TTTTTTTC---A
GTTAATAAAAGGCT-G-TGACC-AGT---GGCTCTGAAATAGGCTATCCTATTCCACAGGCATTT--------CCAAGAG
ACACAAAATAG-CATTTT---TC------------------AAAAGTTCCAGATGAGTTTGGTAATGGTTTCCCTCATTC
CTAGTAGCCATTTCCTTAGTACTAAGAATTTGAACACTTAGATCATTTTTCTTCTGAAAGATTTGGTGATAAAAAAGGAA
TGGGTTCCCCTTTCACGCTACAAATTCTGCTCAAGTCTCGCCATCTGATTTAAGTATGACTTTGATGACATTCCTTTACC
CAGAATTGGGTA-TGA-CCTGCAG
>Cavia porcellus
AGCAAAGG--ATTAAAACCTTTGGGGAAAAAAA-GTTCCCATG--AGA-GATG-AC--------G--ATTGATGA--TCA
GTCTTTAAAACTGT-----------------AAAAGAAGGCAAAATTTCTTGAGGAAA-TGAGAAACATTTTTTGC----
----------------------------ATC---GAACA---

--TCTTG--------------ATTTTTTCCATTA
ATTAATAAGAAGCTGA-TGACTTACT---TGTTCTGAAATGGCCTGACCCATTCCATAGGCATTTGTAACTCTCAAAGCA
A-----------TAAATGTTTA-------------------AGAGTTTTCAGATCACTTTGGTGATGGTCCCTCTTATCC
ACAACAGCCATTTCTTAAGCACTAAAGGCTTGAACAAGTAGATCATGTTTCCTCCCAAAGAGTTTCTGATAACAA-GGAA

TAGGTA-CCCTTTCACAAGACAATTTCTACTCAAGTCTTGGCATCTGATTTAAGCATGAATTTGATGGCATTCCTTTAGC
CTGAATTGGGTAAAGGTTCTGCAG
>Heterocephalus glaber
AGGAAAGA--ATTAAAACTTTTGAGGAAAAGAA-TTTCCCATGGGAAATGATACTAGCAATGGTGGGATTGGTGG--TCA
GTTTTTAAAACTGAAAAA-------------GAAAGAAGGCAGAATTTCTCAAGAAAG-TGAGAAATATTTTT-------
----------------------------ATA-TTGAACA---

--TCTTG-------------TTTGTTTTCCTTTA
GTTAACAGGAAGCTGA-TGACCTACTACTGGTTCTAAAATGGCCTATCCCATTCCATAGGCATTTGAACCTCTCAAAGCA
ATAACA------TAAATGTTTTT------------------AAAGTTTTCAGATCAGTTTGGTGATGGTCTGTCTCATCC
ATAACAGCTATTTCTTAAGCACTAATAATTTGGACAATTAGATCATTTTTCCTCCCAAAGAGTTTCTGATAACAA-GGAA
TGGGTC-TCCTTTCACACGACAGTTTTTACTCAAGTCTTGGCATCTGATTTAAGCATGACTTTGATGGCATTCCTTTAGC
-AGAATTGGGTA-TGATCCTGCAG
>Ictidomys tridecemlineatus
AGGAGTGGGAATTAAAATCCTTGGAGAAAAGGA-TTTCCTGTGGAAGAGGATACAAGAAATAGTGAGATAGGTGA--TCA
GTTCTTAAAGCCAAAAAG-------------GAGTAAGGACAGAATTTCTCAAGAAAA-TTAGAAATATTT-CT------
----------------------------AGC-TTTAACT---

--TCTTG---------------TTTTTTCCTTTA
CTTGACAGGAGTCCGA-TGACCTACT---GGCTCTAAAATGGCCTATTCCATTCCATAGGCATCTGTATCTCTCCAAGAG
GAGAAAGAAAA-TAAATGTTTTC------------------AAAGGTTCCAGATCAGTGTCATGATGGTTTCTCTCATTC
TTAACAGCCATTTTCTTAGCACTAAGAATTTGAACAATTAGATCATTTTTCCTCCAAAAGATTTTCTGATAACAA-GGAA
TGGGTT-CCCTTTCACACTACA--CTC-GCTC-----TTGGCATCTGATTGTAGCACGACTTTGATGACATTCCTTTAGC
CAGAATTGGGTC-TGGCCCTGCAA
>Marmota marmota
AGGACTGGGAATTAAAATCCTTGGAGAAAAGGA-TTTCCTGTGGAAGAGGATACAAGAAATAGTGAGATAGGTGA--TCA
GTTCTTAAAGCCAAAAAG-------------GAGTAAGGACAGAATTTCTCAAGAAAA-TTAGAAATATTT-CT------
----------------------------AGC-TTTAACT---

--TCTTG---------------TTTTTTCCTTTA
CTTAACAGGAGTCCGA-TGACCTACT---GGCTCTAAAATGTCCTATTCCATTCCATAGGCATCTGCATCTCTCCAAGAG
GAGAAAGAAAA-TAAATGTTTTC------------------AAAGGTTCCAGATCAGTGTCATGATGGTTTCTCTCATTC
TTAACAGCCATTTTCTTAGCACTAAGAATTTGAACAATTAGATCATTTTTCCTCCAAAAGATTTTCTGATAACAA-GGAA
TGGGTT-CCCTTTCACACTACA--CTC-GCTC-----TTGGCATCTGATTGAAGCACGACTTTGATGACATTCCTTTAGC
CAGAATTGGGTC-TGGCCCTGCAA
